# Genomic analysis of diet composition finds novel loci and associations with health and lifestyle

**DOI:** 10.1038/s41380-020-0697-5

**Published:** 2020-05-11

**Authors:** S. Fleur W. Meddens, Ronald de Vlaming, Peter Bowers, Casper A. P. Burik, Richard Karlsson Linnér, Chanwook Lee, Aysu Okbay, Patrick Turley, Cornelius A. Rietveld, Mark Alan Fontana, Mohsen Ghanbari, Fumiaki Imamura, George McMahon, Peter J. van der Most, Trudy Voortman, Kaitlin H. Wade, Emma L. Anderson, Kim V. E. Braun, Pauline M. Emmett, Tonũ Esko, Juan R. Gonzalez, Jessica C. Kiefte-de Jong, Claudia Langenberg, Jian’an Luan, Taulant Muka, Susan Ring, Fernando Rivadeneira, Harold Snieder, Frank J. A. van Rooij, Bruce H. R. Wolffenbuttel, 23andMe Research Team, EPIC- InterAct Consortium, Lifelines Cohort Study, George Davey Smith, Oscar H. Franco, Nita G. Forouhi, M. Arfan Ikram, Andre G. Uitterlinden, Jana V. van Vliet-Ostaptchouk, Nick J. Wareham, David Cesarini, K. Paige Harden, James J. Lee, Daniel J. Benjamin, Carson C. Chow, Philipp D. Koellinger

**Affiliations:** 1 Department of Economics, Vrije Universiteit Amsterdam, De Boelelaan 1105, 1081 HV Amsterdam, The Netherlands; 2 Department of Applied Economics, Erasmus School of Economics, Erasmus University Rotterdam, Burgemeester, Oudlaan 50, 3062 PA Rotterdam, The Netherlands; 3 Department of Economics, Harvard University, 1805 Cambridge St, Cambridge, MA 02138, USA; 4 Analytical and Translational Genetics Unit, Massachusetts General Hospital, Richard B. Simches Research building, 185 Cambridge St, CPZN-6818, Boston, MA 02114, USA; 5 Stanley Center for Psychiatric Genomics, The Broad Institute at Harvard and MIT, 75 Ames St, Cambridge, MA 02142, USA; 6 Behavioral and Health Genomics Center, Center for Economic and Social Research, University of Southern, California, 635 Downey Way, Los Angeles, CA 90089, USA; 7 Department of Epidemiology, Erasmus MC, University Medical Center, Wytemaweg 80, 3015 GE Rotterdam, The Netherlands; 8 Erasmus University Rotterdam Institute for Behavior and Biology, Erasmus School of Economics, Erasmus, University Rotterdam, Burgemeester Oudlaan 50, 3062 PA Rotterdam, The Netherlands; 9 Center for the Advancement of Value in Musculoskeletal Care, Hospital for Special Surgery, 535 East 70th Street, New York, NY 10021, USA; 10 Department of Healthcare Policy and Research, Weill Cornell Medical College, Cornell University, 402 East 67th Street, New York, NY 10065, USA; 11 Department of Genetics, School of Medicine, Mashhad University of Medical Sciences, Azadi Square, University Campus, 9177948564 Mashhad, Iran; 12 MRC Epidemiology Unit, University of Cambridge School of Clinical Medicine, Institute of Metabolic Science, Cambridge Biomedical Campus Cambridge, CB2 0QQ Cambridge, UK; 13 Integrative Epidemiology Unit, Population Health Sciences, Bristol Medical School, University of Bristol, Oakfield House, Oakfield Grove, BS8 2BN Bristol, UK; 14 Department of Epidemiology, University of Groningen, University Medical Center Groningen, Hanzeplein 1, 9713 GZ Groningen, The Netherlands; 15 Population Health Sciences, Bristol Medical School, University of Bristol, Oakfield House, Oakfield Grove, BS8, 2BN, Bristol, UK; 16 Estonian Genome Center, University of Tartu, Riia 23b, Tartu 51010, Estonia; 17 Barcelona Institute for Global Health (ISGlobal), Doctor Aiguader, 88, Barcelona 8003, Spain; 18 Universitat Pompeu Fabra (UPF), Ramon Trias Fargas 25-27, Barcelona 8005, Spain; 19 CIBER Epidemiología y Salud Pública (CIBERESP), Pabellón 11, Calle Monforte de Lemos, 3-5, Madrid 280229, Spain; 20 Leiden University College, Anna van Buerenplein 301, 2595 DG Den Haag, The Netherlands; 21 Department of Internal Medicine, Erasmus MC University Medical Center, Wytemaweg 80, 3015 GE Rotterdam, The Netherlands; 22 Department of Endocrinology, University of Groningen, University Medical Center Groningen, Hanzeplein 1, 9713 GZ Groningen, The Netherlands; 23 Genomics Coordination Center, Department of Genetics, University of Groningen, University Medical Center, Groningen, Hanzeplein 1, 9713 GZ Groningen, The Netherlands; 24 Department of Economics, New York University, 19 W. 4th Street, New York, NY 10012, USA; 25 Department of Psychology, University of Texas at Austin, 108 E. Dean Keeton Stop #A8000, Austin, TX 78704, USA; 26 Department of Psychology, University of Minnesota Twin Cities, 75 East River Parkway, Minneapolis, MN 55455, USA; 27 National Bureau of Economic Research, 1050 Massachusetts Ave, Cambridge, MA 02138, USA; 28 Department of Economics, University of Southern California, 635 Downey Way, Los Angeles, CA 90089, USA; 29 Laboratory of Biological Modeling, National Institute of Diabetes and Digestive and Kidney Diseases, National, Institutes of Health, Bethesda, MD 20892, USA

## Abstract

We conducted genome-wide association studies (GWAS) of relative intake from the macronutrients fat, protein, carbohydrates, and sugar in over 235,000 individuals of European ancestries. We identified 21 unique, approximately independent lead SNPs. Fourteen lead SNPs are uniquely associated with one macronutrient at genome-wide significance (*P* < 5 × 10^−8^), while five of the 21 lead SNPs reach suggestive significance (*P* < 1 × 10^−5^) for at least one other macronutrient. While the phenotypes are genetically correlated, each phenotype carries a partially unique genetic architecture. Relative protein intake exhibits the strongest relationships with poor health, including positive genetic associations with obesity, type 2 diabetes, and heart disease (*r_g_* ≈ 0.15–0.5). In contrast, relative carbohydrate and sugar intake have negative genetic correlations with waist circumference, waist-hip ratio, and neighborhood deprivation (∣*r_g_*∣ ≈ 0.1–0.3) and positive genetic correlations with physical activity (*r_g_* ≈ 0.1 and 0.2). Relative fat intake has no consistent pattern of genetic correlations with poor health but has a negative genetic correlation with educational attainment (*r_g_* ≈−0.1). Although our analyses do not allow us to draw causal conclusions, we find no evidence of negative health consequences associated with relative carbohydrate, sugar, or fat intake. However, our results are consistent with the hypothesis that relative protein intake plays a role in the etiology of metabolic dysfunction.

## Introduction

Understanding the relationships between nutrition, lifestyle, and health is among the highest priorities for public health [1]. Many aspects of dietary intake have been studied, but the health impacts of macronutrient composition (i.e. relative intake from fat, protein, and carbohydrate) have been especially controversial in the last few decades [2-4]. Despite a lack of robust empirical evidence from randomized trials on the long-term effects of macronutrient restriction on body weight and health [5-7], dietary recommendations have shifted from low-fat to low-sugar and, more recently, lower animal-protein diets [8-13]. Connections between diet and mental health are also increasingly recognized [14]. Genetic correlation analysis allows links between mental health and dietary intake to be estimated without the need to observe psychiatric measures and macronutrient intake in the same samples. These links can then corroborate existing ideas or fuel new hypotheses about the relationships between diet and mental health.

Previous work has found that diet composition is heritable (range *h*^2^ = 27–70%) [15-17] and may share genetic components with health and lifestyle [18]. The largest GWAS on relative intake from protein, fat, and carbohydrates (up to *N* = 91,114) to date has identified three robustly associated SNPs in or near *RARB*, *FTO*, and *FGF21*, each of which captures only a miniscule part of trait heritability (*R*^2^ < 0.06%) [19-21]. These results suggest that diet composition is a genetically complex phenotype that requires large GWAS sample sizes for robust genetic discovery. However, proper measurement of nutrient intake requires a long and detailed questionnaire [22]. Therefore, relatively few large genotyped cohorts have collected this information, which restricts available GWAS sample sizes.

Here, we perform the largest dietary intake GWAS to date, using the vast majority of currently available European-ancestry genotyped diet data. Power calculations determined a minimum required sample size of *N* = 141,000 (Supplementary Information 1.3). We nearly triple the GWAS sample size compared to earlier work [21] to *N* = 264,181 for relative intake of protein, carbohydrate, and fat, increasing the number of robustly associated independent loci from three to 18. Furthermore, we report the first GWAS results for relative sugar intake (*N* = 230,648), which is a subcomponent of our carbohydrate phenotype and captures relative intake of both naturally occurring and added sugars. The sugar GWAS identifies three additional, unique lead loci. In our largest dataset, the UK Biobank (*N* = 173,253) [23], we also report an auxiliary GWAS for saturated fat intake, a subcomponent of our fat phenotype, which we only use for genetic correlation analyses. We also report phenotypic associations between BMI and macronutrient subtypes (plant vs. animal-protein; saturated vs. unsaturated fat; natural vs. added sugars).

Biological annotation of our GWAS results indicates that the brain is the main driver of diet composition’s genetic signal. Furthermore, we find robust genetic and phenotypic associations between relative protein intake and poor health, but no clear pattern of associations of the other macronutrients with health. Finally, we probe the robustness of these results to possible confounds due to socioeconomic status and physical activity.

## Methods

This article is accompanied by a Supplementary Information, which describes further methodological details.

### Phenotype definitions, GWAS, quality control, and meta-analysis

We performed GWAS in European-ancestry individuals for four dietary composition phenotypes: relative intake of fat, carbohydrate, and sugar. As an auxiliary analysis, we performed GWAS for relative intake of saturated fat in the UKB. Discovery analyses were performed in UKB, while replication analyses were conducted in cohorts from the Netherlands (Lifelines, RSI/II/III), UK (ALSPAC, Fenland), USA (FHS, HRS, GARNET, HIPFX, WHIMS+), and the international consortia EPIC-InterAct and DietGen (Supplementary Information 1 and Supplementary Table 1.1). Since DietGen only analyzed fat, protein, and carbohydrate intake (measured by DietGen with intake as a percentage of total energy intake), our final sample sizes are *N_sugar_* = 235,391 and *N_fat_* = *N_protein_* = *N_carbohydrate_* = 268,922. A study flowchart is presented in Extended Data Fig. 2, which shows that we used the meta-analysis results of UKB + replication cohorts throughout our investigations except for the replication and sensitivity analyses and the auxiliary GWAS for saturated fat.

Cohorts measured previous-day (UKB) or habitual (all other cohorts) dietary intake with comprehensive food-item questionnaires (Supplementary Table 1.2). Phenotype definitions are described in Supplementary Information 2. With the exception of DietGen, all cohorts corrected macronutrient intakes for total energy intake allowing for non-linear effects, and GWAS was performed according to a prespecified analysis plan (Supplementary Information 2.6).

Cohort-level quality-control (QC) was performed in accordance with protocols developed by the GIANT consortium [24] and the Social Science Genetic Association Consortium (SSGAC, Supplementary Information 3.3). Filters for participants and SNPs varied by cohort and cohort sample size. SNP effects were summarized across cohorts using fixed-effects sample-size-weighted meta-analyses based on *Z*-statistics. For the family cohorts UKB and FHS, we used the median effective sample size as a weight (Supplementary Information 3.4).

### Replication

We assess the credibility of individual SNPs from our discovery GWAS by replicating the associations of its lead SNPs in our replication GWAS (Supplementary Information 4, Supplementary Information 4.1). Our replication analyses closely followed the procedure outlined in Supplementary Information section 1.8 of Okbay et al. [25]. We conducted one-sided binomial tests for both the sign concordance of the lead SNPs and the number of lead SNPs from our discovery GWAS that differ at the *P* < 0.05 threshold (both with and without Bonferroni correction) in the replication GWAS. In addition to conducting binomial tests, we simulated the expected rate of replication given the discovery GWAS results, the discovery sample size and the replication sample size, and we assessed whether these expected rates matched the observed replication rates. We used bivariate LD Score regression to examine the comparability between the summary statistics from our discovery cohort, the 14 replication cohorts, and DietGen (Supplementary Table 4.2). We also report the replication record of a rare variant in *DRAM1* discovered by Merino et al. [21] (Supplementary Table 4.3).

### Population stratification

LD Score regression was used to estimate inflation of the GWAS results due to population stratification (Supplementary Table 5.1). We adjusted the reported standard errors and *P*-values of meta-analyzed SNPs for bias due to population stratification by dividing them by the square root of the LD Score regression intercept. To identify approximately independent lead SNPs, we applied the clumping algorithm in PLINK (parameters *r*^2^ > 0.1, *P*-value < 5 × 10^−8^). Supplementary Tables 5.2 and 5.4 report the lead SNPs and the overlapping loci between phenotypes.

### Sensitivity analyses

We performed sensitivity analyses for the two SNPs that reached genome-wide significance in *APOE* (rs429358) and *ADH1B* (rs1229984, Supplementary Table 5.5). For the *APOE* SNP, we assessed its effect size and confidence interval in a subsample of the UKB aged below 60 years. For the *ADH1B* SNP, we assessed its effect size and confidence interval in a subsample of the UKB who report to be non-drinkers. We tested whether the confidence intervals of the effect sizes overlap with the confidence intervals in the meta-analyzed sample.

### Biological annotation

All bioinformatics analyses used the results of the combined meta-analysis (Supplementary Information 6). To annotate the top GWAS findings, we performed MAGMA [26] gene-based analysis to test 18,224 genes for association with diet composition (Bonferroni-corrected *P*-value threshold = 0.05/18,224). To gain preliminary insights into the likely functions of the significant MAGMA genes, we queried them in Gene Network. To gain insights into probable functional genomic categories and tissues, we estimated stratified LD Score regressions for the 52 functional genomic regions of the “baseline model”, the 10 broad tissue-level annotations from Finucane et al. [27], and the 53 fine tissue-level annotations from GTEx, with Bonferroni-corrected *P*-value thresholds = 0.05/53, 0.05/10, 0.05/52, respectively (Supplementary Tables 6.1-6.3). To annotate the lead GWAS SNPs, we queried whether they (or SNPs in LD with them) are associated with gene expression in relevant GTEx tissues, or in LD with protein-altering SNPs (Supplementary Tables 6.3-6.6).

### Estimation of genome-wide SNP heritability

We used GCTA-GREML [28] and LD Score regression [29] to estimate the SNP-based heritability of diet composition (Supplementary Information 8, Supplementary Table 8.1). We restricted the GCTA analysis to genotyped SNPs with MAF > 0.01 and a random subset of 30,000 UKB individuals in the UKB, and thereafter drop one individual in each pair of individuals with a cryptic relatedness exceeding 0.025, resulting in *N* = 28,635. For the LD Score regression analysis, we used the full meta-analysis results, HapMap3 SNPs with MAF > 0.01, and LD estimates from the 1000 Genomes project provided by Finucane et al. [27].

### Genetic correlations between macronutrients

We used bivariate LD Score regression to estimate the genetic correlations between macronutrients (Supplementary Information 7).

### Polygenic prediction

We assessed the accuracy of polygenic scores of diet composition in the HRS and RSI validation cohorts and used LDpred [30] to construct polygenic scores assuming an underlying infinitesimal model (Supplementary Information 9, Supplementary Table 9.1). Since these cohorts are included in the full meta-analysis, we conducted a new meta-analysis that excludes the holdout cohort to obtain the SNP weights. Analyses are restricted to HapMap3 SNPs with MAF > 0.05, and LD scores were calculated on the basis of the holdout cohort. Our measure of a score’s predictive power is the incremental adjusted *R*^2^ from adding the score to a regression of the phenotype on the covariates sex, birth-year, birth-year squared, and cubed, as well as the interactions between sex and the three birth-year variables, and the first ten principal components of the genetic relatedness matrix. We bootstrapped 95% percentile confidence intervals for the incremental *R*^2^ estimates with 1000 iterations.

### Genetic correlations

We used bivariate LD Score regression to estimate genetic correlations between: diet composition and various health and behavioral phenotypes (Supplementary Information 10, Supplementary Tables 10.2-10.4). We used the 1000 Genomes LD scores computed by Finucane et al. [27] and restricted analyses to HapMap3 SNPs with MAF > 0.01.

### Phenotypic associations

We examined the phenotypic associations between relative macronutrient intake and BMI in four large, independent cohorts from the UK and US (UKB, HRS, FHS, and WHI, with combined *N* = 173,165; Supplementary Information 11, Supplementary Table 11.1). In the HRS, FHS, and WHI, we were also able to distinguish animal vs. plant protein, natural vs. added sugars, and saturated vs. unsaturated fat. In the UKB, only the distinction between saturated vs. unsaturated fat was available (Supplementary Table 11.2). We estimated the standardized regression coefficients obtained from a multiple regression of BMI on the focal macronutrient, sex, age, educational attainment, household income (available for all cohorts except FHS), and the number of dietary measurements. In the UKB, we performed an additional regression that included a measure of physical activity. We restricted the samples to individuals also included in the GWAS. We used Fisher’s *Z*-transformation to perform fixed-effects, inverse-variance weighted meta-analysis of the standardized regression coefficients. Fisher’s *Z*-transformation was also used to obtain 95% confidence intervals.

## Results

### Phenotype definition

All cohorts used self-report questionnaires containing ≥70 food items. Average intakes were highly similar across cohorts (Supplementary Table 1.2). Using these self-reports, we calculated the relative contributions of fat, protein, carbohydrate, and sugar to total energy intake. When possible, we excluded individuals on calorie- or macronutrient-restricted diets (see Supplementary Table 1.3 for all exclusion criteria).

We do not study total energy intake because it is mainly determined by body size and physical activity [31], and because systematic underreporting of total energy intake is correlated with BMI [32]. We caution that selective underreporting of macronutrients could be problematic for the common approach we adopt of studying relative intake, but there is mixed evidence for this, and its consequences are poorly understood (Supplementary Information 2.4).

**Extended Data Figure 1 ∣:**
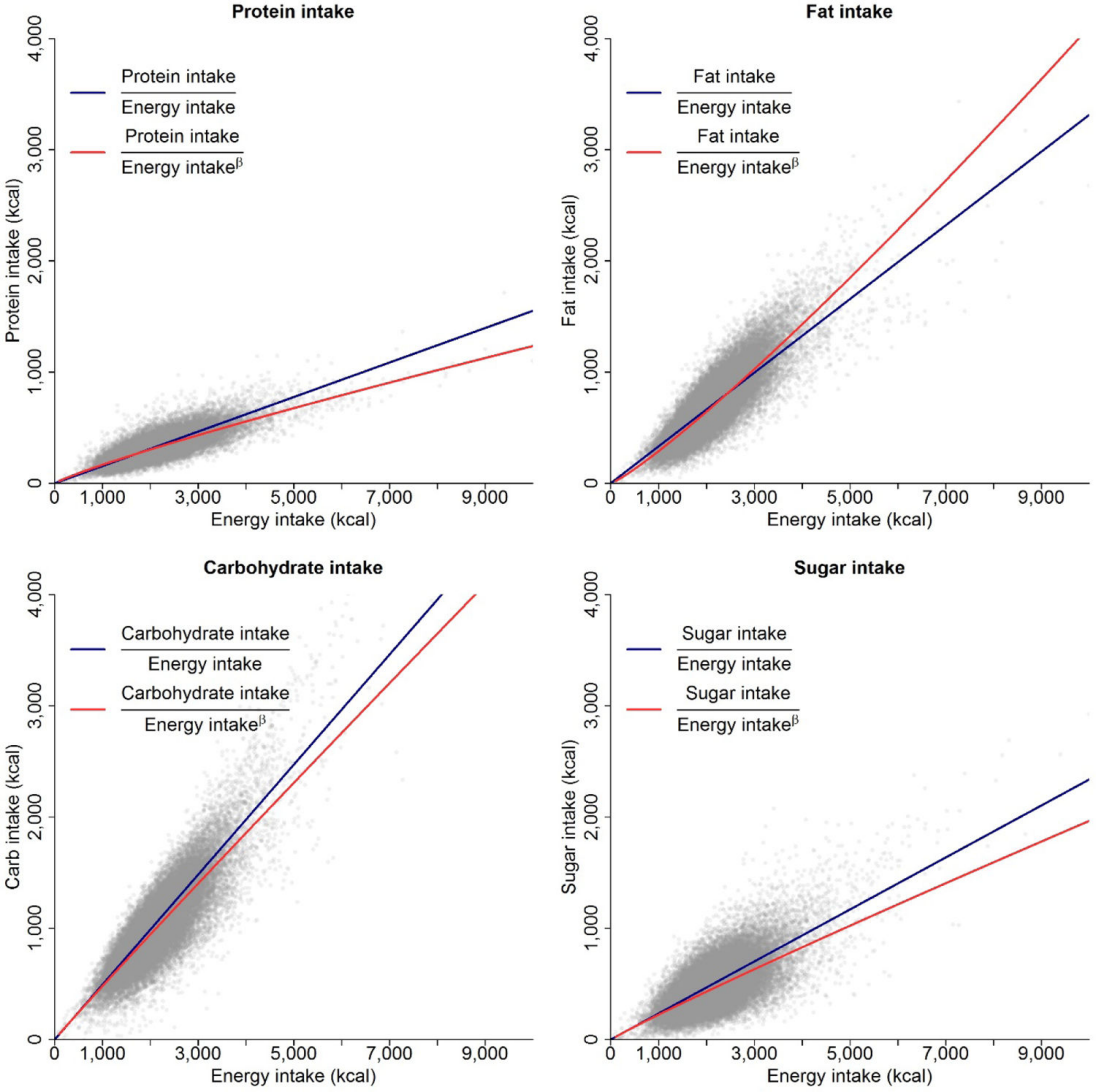
Non-linear phenotypic scaling between macronutrient intake and total energy intake. Scatterplots of macronutrient intake (*y*-axis) versus total energy intake (*x*-axis) in European-ancestry individuals from the first release of genetic data from the UK Biobank (*N* = 43,788). The blue and red lines denote the different lines of fit. The blue line represents the average “macronutrient density” (i.e., average energy intake from the focal macronutrient as observed in the full sample, across the different values of *x_i_*). The red line represents the regression line of the multiplicative regression model of macronutrient intake on energy intake, where $y_{i}=\hat{\alpha }*x^{\hat{\beta }}$.

Since macronutrient intake may not scale linearly with total energy intake, we developed and applied a method that adjusts for observed non-linear relationships (Supplementary Information 2.6-2.8, Extended Data Fig. 1). Consistent with the satiating properties of protein [33], we find that relative protein intake declines at higher levels of total energy intake, while relative fat intake increases, and relative sugar and carbohydrate intake remain roughly constant (Supplementary Table 2.3).

### Main results

**Extended Data Figure 2 ∣:**
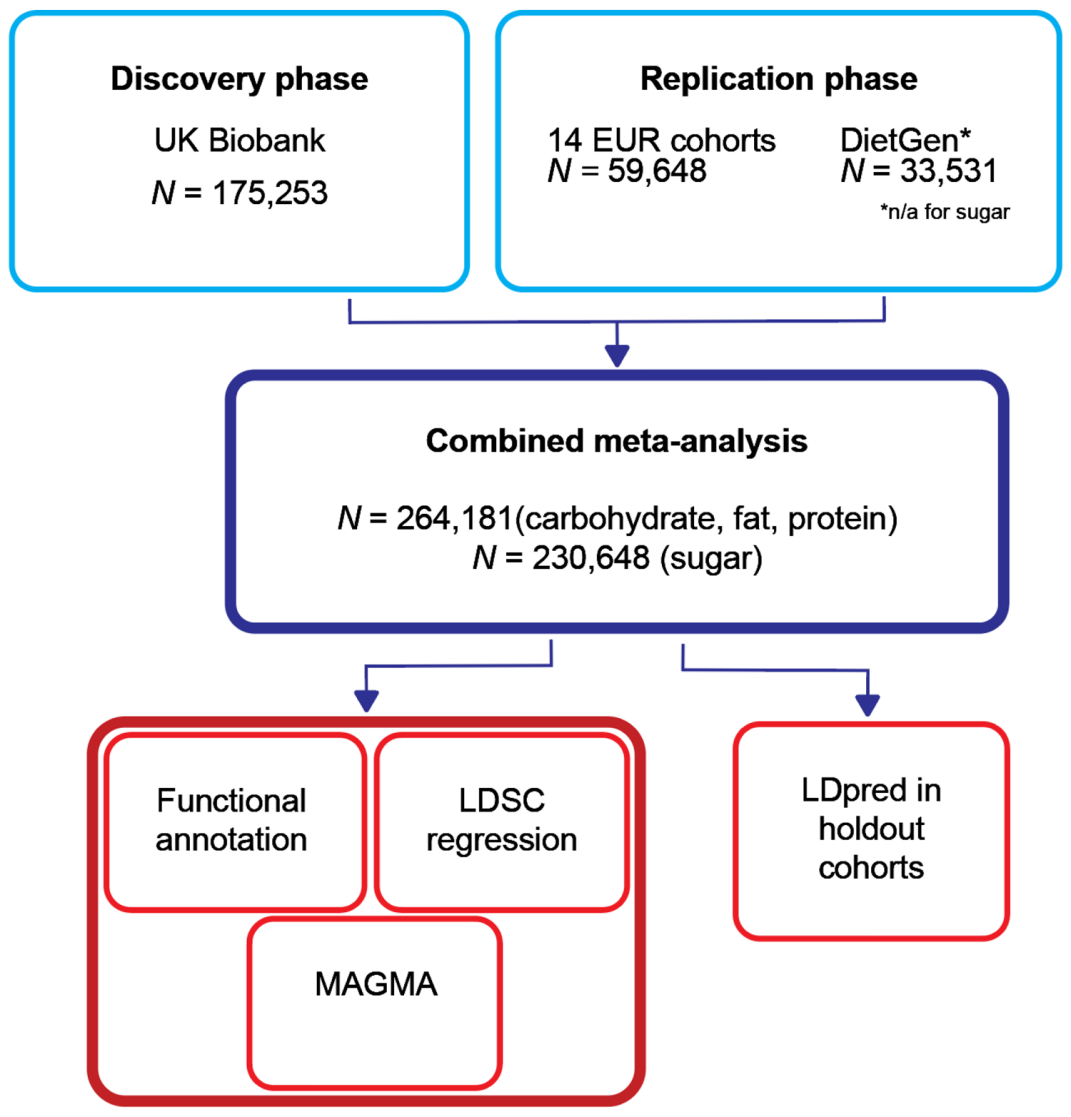
Flow chart. Schematic representation of the study design.

GWAS were performed in individuals of European ancestries from over 14 population cohorts. Informed consent was obtained by the cohorts for all participants included in the analyses. Association statistics underwent rigorous quality control according to SSGAC guidelines [25, 34, 35], which included sample-size-dependent quality-control filters, exclusion of SNPs with too small standard errors or too large explained phenotypic variance, and visualizations of summary statistics and allele frequencies (Supplementary Information 3.3, Supplementary Tables 3.1-3.5). Our discovery sample is the subset of the UKB with survey data on dietary intake (*N* = 175,253). The replication phase consists of a meta-analysis of GWAS summary statistics from 14 additional cohorts that followed our analysis plan (*N* = 60,138) together with summary statistics from DietGen [20] (for fat, protein, and carbohydrate, *n* = 33,531, flowchart in Extended Data Fig. 2). DietGen [20] assumed a linear scaling of macronutrients with total energy intake. Nonetheless, we included DietGen in our meta-analysis because the genetic correlations between DietGen and our other replication cohorts are not significantly different from one at *P* < 0.05 (Supplementary Table 4.1).

**Extended Data Figure 3 ∣:**
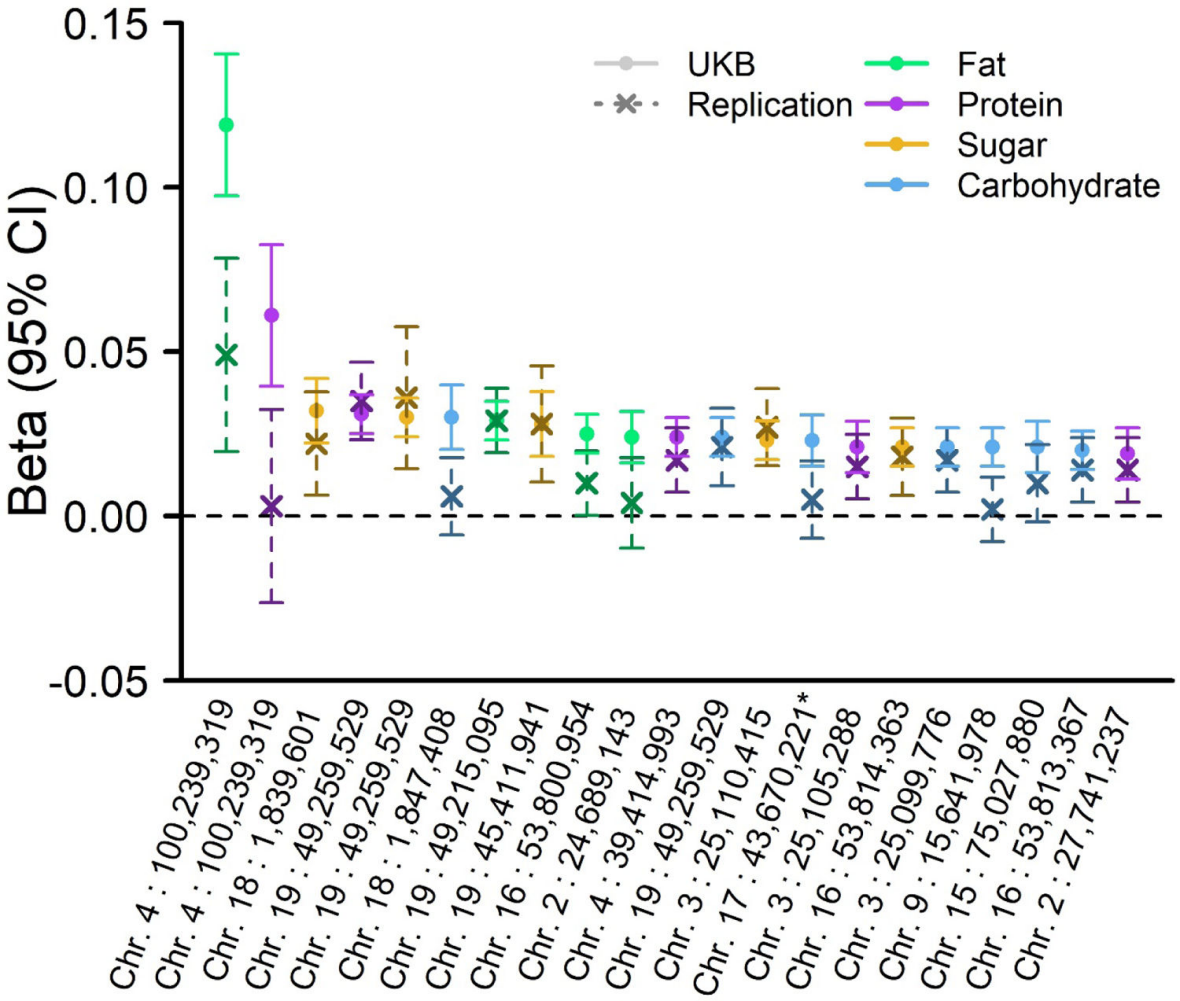
Replication of 21 lead SNPs from the discovery GWAS of diet composition. Estimated effect sizes (measured in standard deviations of the diet composition phenotype per effect allele) and 95% confidence intervals for 20 diet composition lead SNPs and 1 proxy-lead SNP (noted with an asterisk, *), in the discovery versus the combined replication phase. SNPs are listed from left to right in descending order of their beta in the discovery GWAS, and coded in such a way that the effect allele is always trait-increasing in the discovery phase. Of the 21 lead or proxy-lead SNPs, all have the anticipated sign in the replication phase, and 16 replicate at the 0.05, one-sided significance level. See Supplementary Information section 4 for additional details.

The discovery stage identified 21 approximately independent genome-wide-significant lead SNPs (see Supplementary Information 3.3.5 for a description of the clumping algorithm): 4 for fat, 5 for protein, 5 for sugar, and 7 for carbohydrate (Supplementary Table 4.2). These lead SNPs partially overlap across phenotypes and reside in 14 unique loci. In the replication stage, all 21 lead SNPs had the anticipated signs and comparable effect sizes (Extended Data Fig. 3), and 15 reach statistical significance at *P* < 0.05 (Supplementary Table 4.2). This empirical replication record matches or exceeds theoretical predictions that take into account the statistical winner’s curse, sampling variation, and statistical power [25] (Supplementary Table 4.1). In our data, the association between *DRAM1* and dietary intake reported by Merino et al. [21] does not replicate, with a discordant effect size compared to Merino et al. ($\hat{\beta }$ = −0.028, *SE* = 0.025 compared to Merino et al.’s $\hat{\beta }=0.122$, *SE* = 0.02 in phenotypic standard deviations per effect allele, Supplementary Table 4.3).

**Extended Data Figure 4 ∣:**
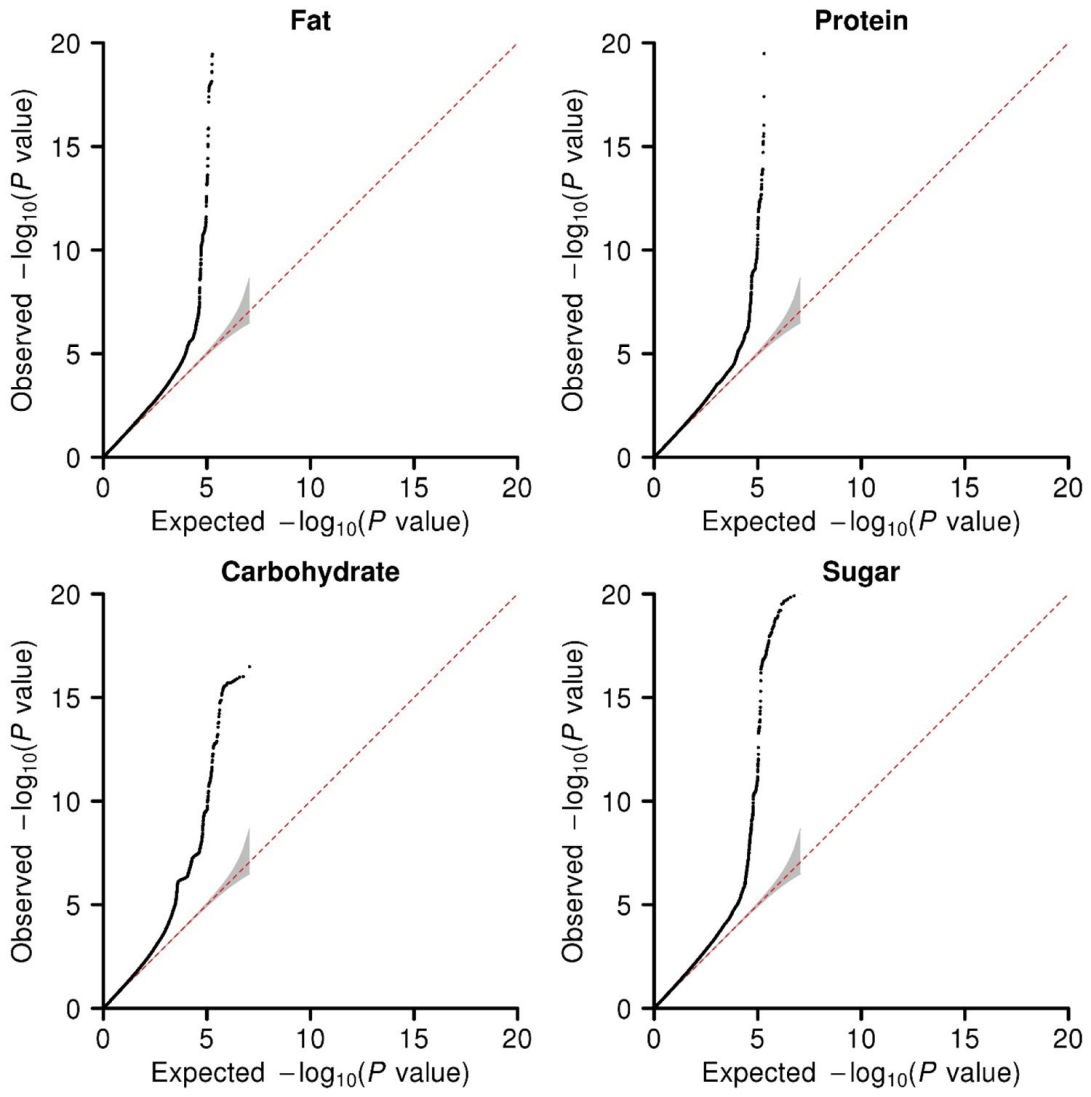
QQ-plot Quantile-Quantile (QQ) plot for the *P* values from the GWAS (for the full meta-analysis samples, inflated by the LDSC intercept) for the four different diet composition phenotypes, which compares the expected *P* values (based on the *P* value beta distribution) to the observed *P* values from the GWAS. Deviation from the expected values (represented with the red, slanted diagonal) represents polygenicity of the trait, and potentially bias due to (for instance) remnant population stratification. The shaded region represents the 95% confidence interval region.

**Extended Data Figure 5 ∣:**
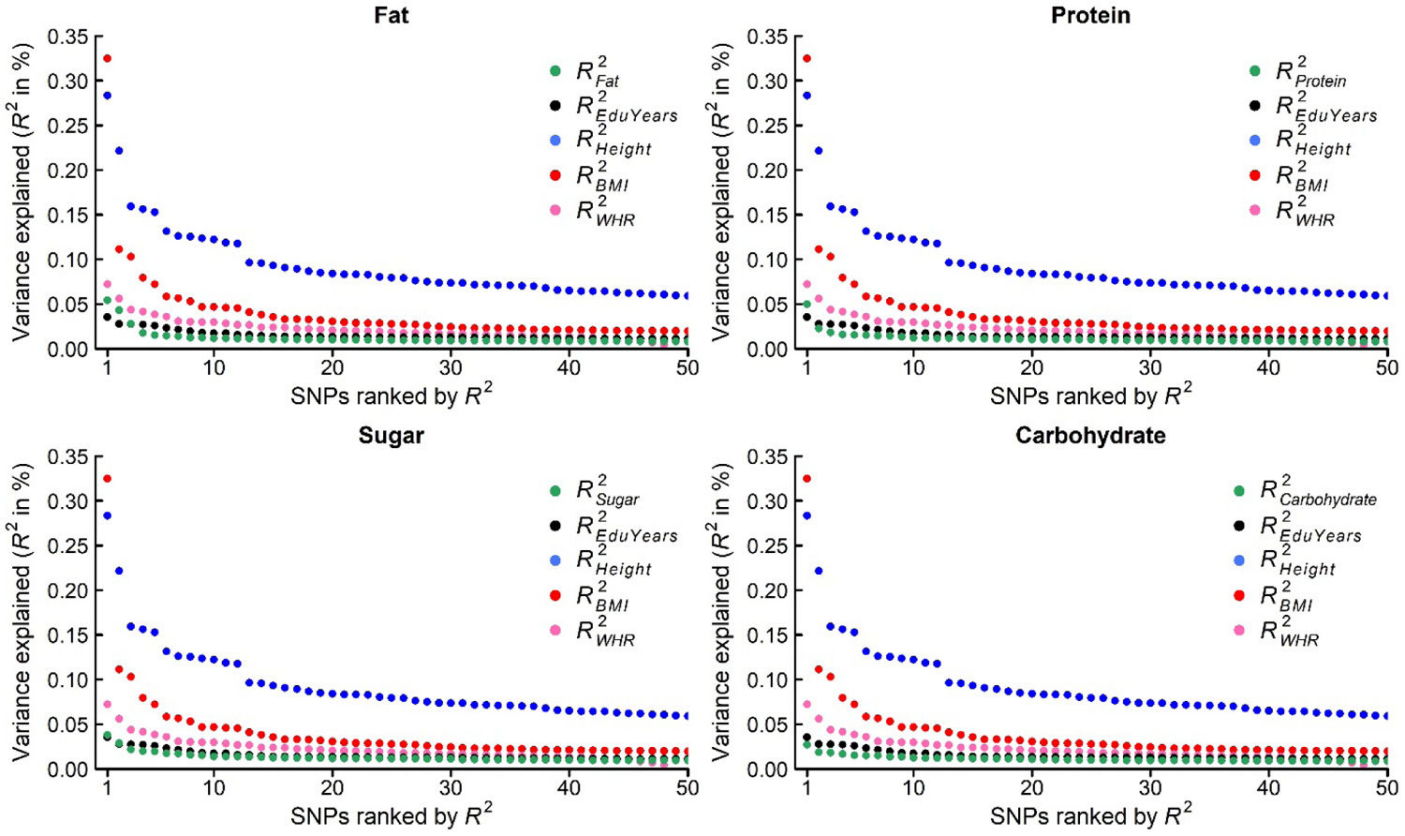
Distribution of effect sizes of the top 50 diet composition lead SNPs, compared with various phenotypes. Variance explained (*R*^2^), with the SNPs ranked by their explained phenotypic variance (*R*^2^). The diet composition effect sizes are compared against the top 50 previously reported for height and for body mass index (BMI), educational attainment (EduYears), and waist-to-hip ratio adjusted for BMI (WHR). The effect sizes for height, BMI, and WHR are based on the GIANT consortium’s publicly available results for pooled analyses restricted to European-ancestry individuals; the effect sizes for EduYears are from Okbay *et al*.

**Fig. 1:**
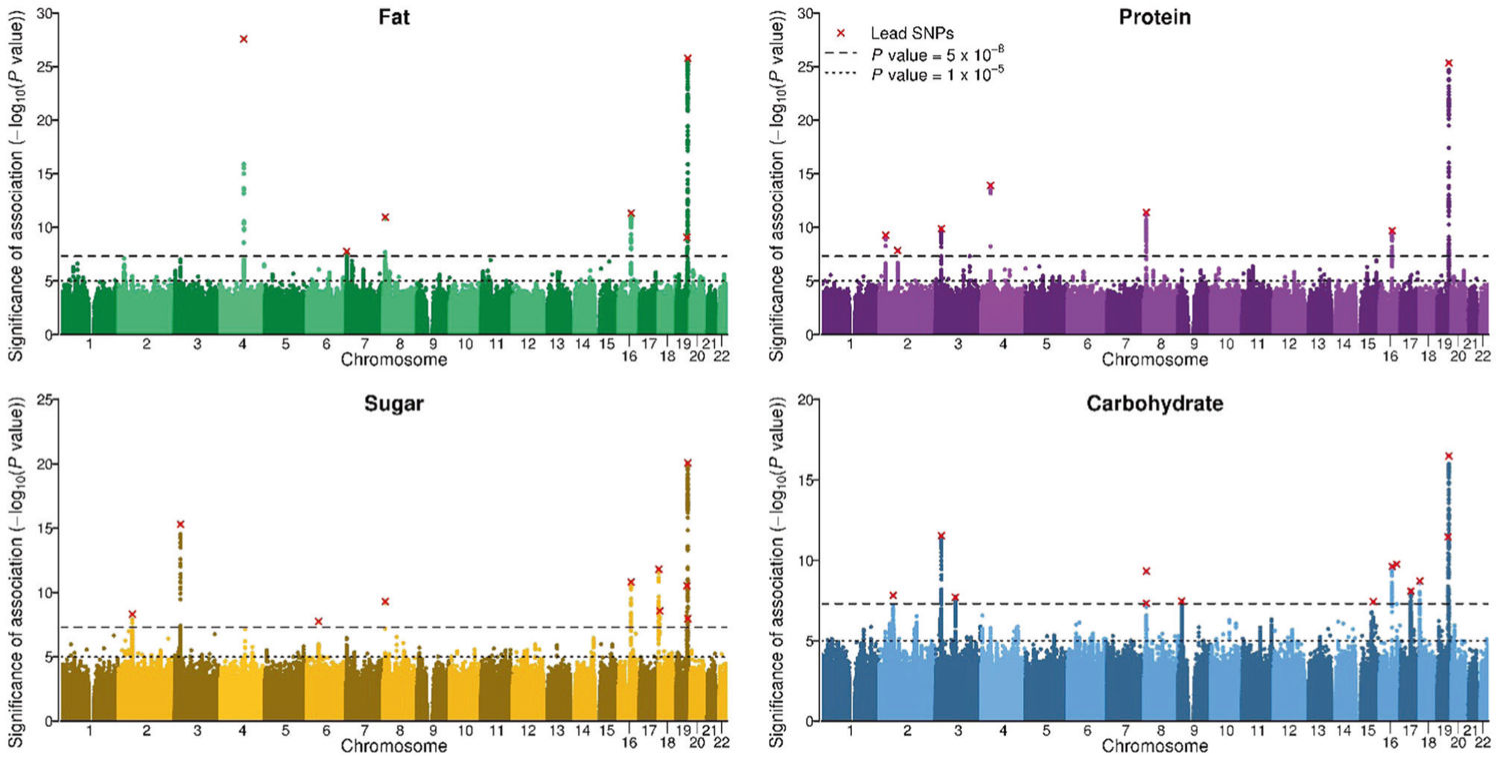
Manhattan plots. The *x*-axis is SNP chromosomal position; the *y*-axis is the SNP *P*-value on a −log_10_ scale; the horizontal dashed line marks the threshold for genome-wide (*P* = 5 × 10^−8^) and suggestive (*P* = 1 × 10^−5^) significance; and each approximately independent (pairwise *r^2^* < 0.1) genome-wide significant association (“lead SNP”) is marked by a red cross.

In order to maximize statistical power, all follow-up analyses below are based on the combined discovery and replication samples (*N* = 235,391–268,922, Supplementary Information 5). The quantile–quantile plots exhibit substantial inflation (λ_GC_ = 1.12–1.19, Extended Data Fig. 4). The estimated intercepts from LD Score (LDSC) regressions [29] suggest that the vast majority of this inflation is due to polygenic signal, and only a small share is attributable to population stratification (the maximum estimate, ~6%, is for fat and is not statistically distinguishable from 0% at *P* < 0.05; Supplementary Table 5.1). The number of approximately independent lead SNPs in the combined sample is 36 (pairwise *r^2^* < 0.01), including 6 for fat, 7 for protein, 10 for sugar, and 13 for carbohydrate (Table 1, Fig. 1). These 36 reside in 21 unique loci (Supplementary Table 5.4). Fourteen lead SNPs are uniquely associated with one macronutrient at genome-wide significance (*P* < × 10^−8^), while five of these reach suggestive significance (*P* < 1 × 10^−5^) for at least one other macronutrient. The SNP effect sizes range from 0.015 to 0.098 phenotypic standard deviations per allele. The phenotypic variance explained per SNP, expressed in terms of coefficient of determination (*R^2^*), ranged from 0.011% to 0.054%, the same order of magnitude as the *R^2^*’s of the most strongly associated lead SNPs for other genetically complex traits such as BMI and educational attainment (Extended Data Fig. 5).

**Extended Data Figure 6 ∣:**
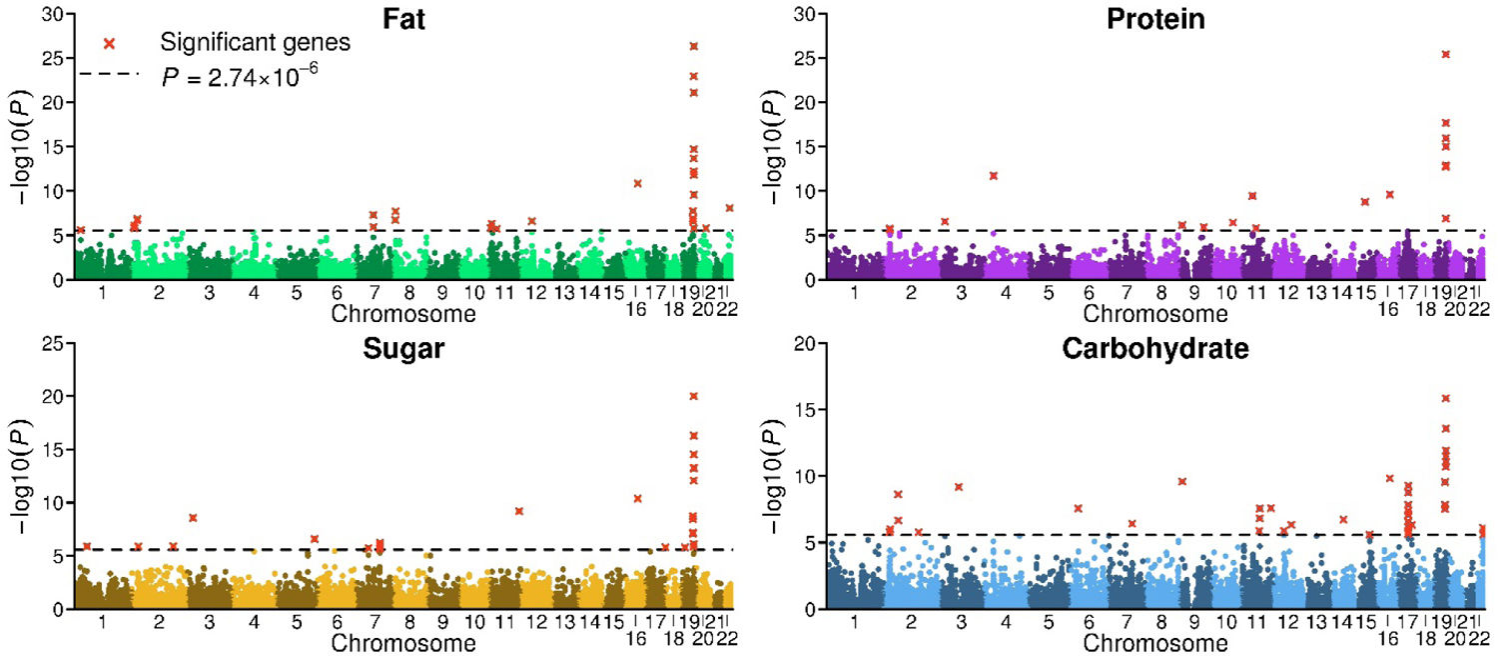
Manhattan plot of the MAGMA gene-based analysis of diet composition. The *x*-axis displays chromosomal position of the gene midpoint, and the *y*-axis displays −log_10_ *P* value of each gene. The dashed line marks the Bonferroni-corrected significance threshold, where the correction is for 18,224 genes. Significant genes are marked with a red cross.

MAGMA [26] gene-based analyses of our GWAS summary statistics identifies 81 unique genes (Extended Data Fig. 6 and Supplementary Table 5.5). While the majority of these genes are near our lead SNPs, MAGMA also identifies 33 genomic regions harboring 44 unique genes that are physically distant (>1 Mb) from our lead SNPs.

### Discussion of lead SNPs from combined meta-analysis

Seven of the 21 lead SNPs had not been (directly or via LD partners, *r*^2^ ≥ 0.6 and distance <250 kb) associated with any other traits in the NHGRI-EBI GWAS Catalog at the time of query (September 19, 2017) [36] (Supplementary Table 5.6). Each of these seven SNPs is located in or near genes that have not been studied in depth to date.

Five lead SNPs are located in or near genes that have well characterized biological functions in nutrient metabolism or homeostasis but have not previously been associated with dietary intake. First, we find that a missense variant in *APOE* (rs429358) is associated with fat, sugar, and carbohydrate, where the allele that decreases Alzheimer’s risk is associated with greater relative fat and lower relative sugar and carbohydrate intakes. In addition to its strong association with Alzheimer’s disease [37], *APOE* is known to be involved in fatty acid metabolism. We explored whether the associations in our data may be driven by sample selection. Specifically, older people with dementia may be systematically missing from the UKB, and unaffected elderly people may have different eating habits than younger people. To test for this possibility, we examined the subsample of UKB participants aged below 60, where such sample selection should be largely absent. We find that the association is indeed smaller in this subsample, but the 95% confidence interval of the effect size overlaps with that of the effect size in the subsample of UKB participants aged 60 and older (Supplementary Table 5.7).

Second, a well-known missense variant (rs1229984 in *ADH1B*) that limits alcohol metabolism is positively associated with fat intake. The association is weaker in a sample of UKB alcohol abstainers (*N* = 39,679; Supplementary Table 5.7), suggesting that it may be partially driven by substitution of fat for alcohol.

Third, one of the protein lead SNPs (rs13146907) is in *KLB*, which codes an essential cofactor to FGF21 [38, 39], which influences sweet and alcohol taste preference via the liver-brain-endocrine axis [40-42]. *KLB* is only associated with protein in our GWAS and MAGMA analyses, while *FGF21* is strongly associated with all four macronutrients in both the GWAS and MAGMA analyses. With MAGMA, we also identified *MLXIPL* (only for fat), a gene that codes a transcription factor to FGF21 [43]. This combination of findings suggests that different genes involved in the same pathway are important for directing intake of different macronutrients.

Fourth, an intergenic variant (rs2472297) that has been linked to higher caffeine consumption [44, 45] is associated with lower carbohydrate intake. There are various possible explanations, such as interrelated lifestyle choices pertaining to food and caffeinated drinks.

Fifth, an intronic variant in *GCKR* (rs780094), a carbohydrate-metabolism gene, is associated with protein. The lead SNP is in almost perfect LD (*r^2^* = 0.94) with a missense variant that has been associated with lipid levels [46] and type 2 diabetes [47].

### Bioinformatic analyses

Animal studies indicate that the brain and peripheral organs interact in directing macronutrient intake [48, 49]. A question that arises is whether the “periphery”, which digests and metabolizes macronutrients, plays a larger role than the brain, for instance by determining how the brain assigns reward values to macronutrients. (For example, this is partially the case with alcohol, where mutations that affect metabolic capacity render alcohol consumption unpleasant [50, 51].) While individual loci associated with dietary intake have been studied previously (e.g., [21, 52]), it is unknown in which tissues the polygenic signal is enriched. To address this question, we used stratified LDSC [27, 53] to identify in which tissues diet-composition-associated SNPs are likely to be expressed (Supplementary Information 6.1). We performed two stratified LDSC analyses, which partitioned SNP heritability according to (i) 10 broadly-defined tissues, which were ascertained with LDSC reference data from chromatin data [54] and (ii) 53 tissues (including 14 brain regions), as ascertained with LDSC reference data from sets of Specifically Expressed Genes in GTEx (known as LDSC-SEG) [53]. To correct for multiple testing across tissues, we used Bonferroni-adjusted significance thresholds for the number of tested tissues (*α* = 0.05/10 = 0.005 and *α* = 0.05/53 = 9.4 × 10^−4^, respectively).

**Fig. 2:**
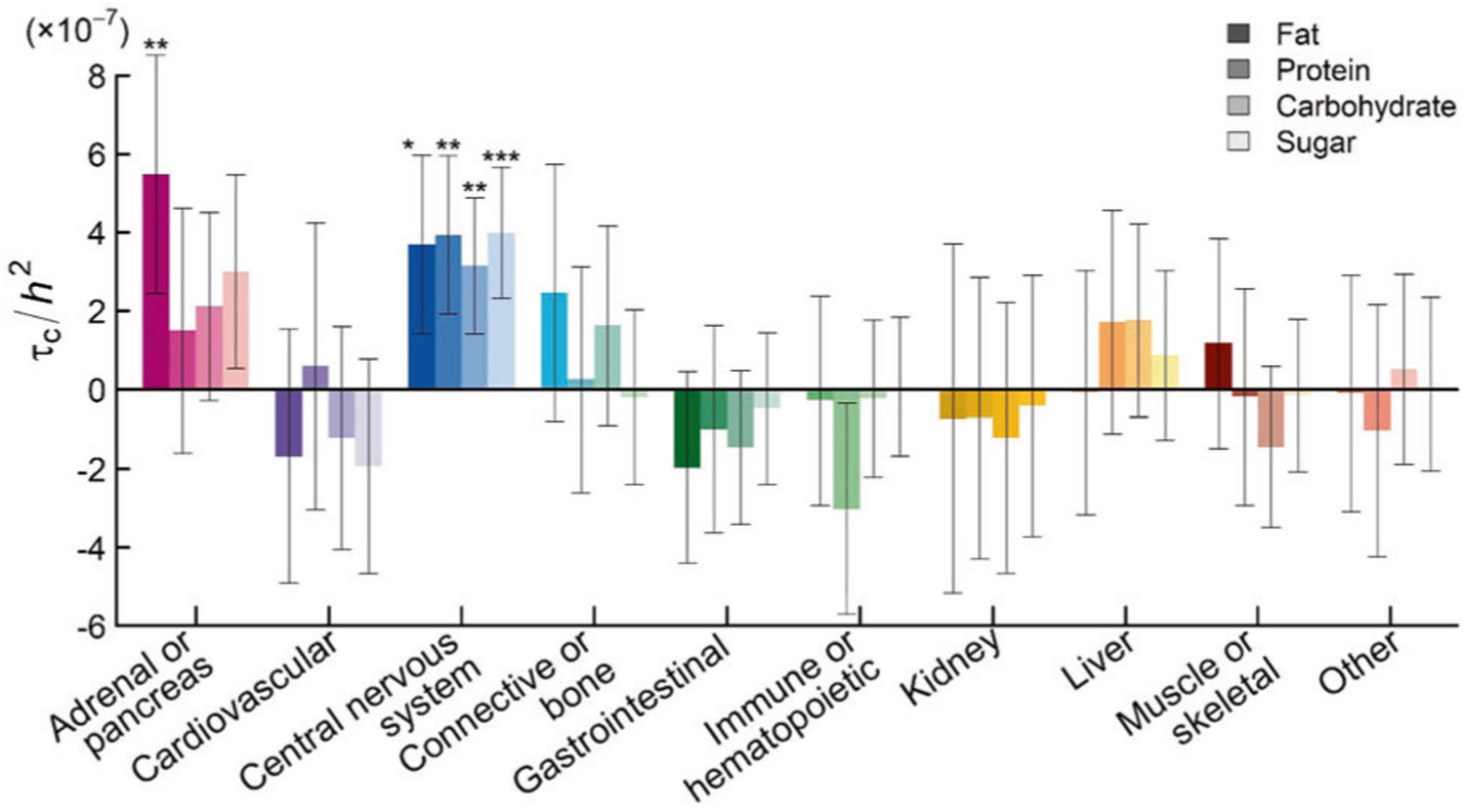
LD Score partitioning of heritability. Functional partitioning of the heritability of diet-composition phenotypes with stratified LD Score regression, where tissues were ascertained by Finucane et al. on the basis of chromatin data. The panel shows the partial regression coefficient (*τ_C_*) from the stratified regression, divided by the LD Score heritability of the diet-composition phenotype (*h*^2^). Error bars depict 95% confidence intervals. The phenotypes are ordered from left to right (fat, protein, sugar, and carbohydrate), from darker to lighter shades. Asterisks (*) denote significant deviation from zero after Bonferroni correction for 10 tissues: $*\;P<\frac{0.05}{10}$, $**P<\frac{0.01}{10}$, $***P<\frac{0.001}{10}$.

We find that the central nervous system explains the majority of the genetic signal for all macronutrients (for the regression coefficients; Fig. 2), with the proportions of explained heritability ranging from 44% (fat and sugar) to 55% (protein). Within the central nervous system, we find broad involvement of the brain, including (frontal) cortex (fat and sugar), the basal ganglia (fat), limbic system (fat and sugar), cerebellum (protein), and hypothalamus and substantia nigra for fat and protein (and sugar only suggestively after Bonferroni correction). The confidence intervals for the coefficients overlap across brain regions, so we cannot draw conclusions about the specificity of brain regions for intake of particular macronutrients.

For fat, genetic variation related to adrenals and/or pancreas tissue is estimated to explain 37% of the heritability. Because the adrenals play a role in lipid metabolism, and the pancreas is crucial for digestion, either tissue may plausibly affect fat intake. We caution, however, that in the LDSC-SEG analyses of 53 tissues, all non-brain regions had *P*-values above 0.05 even before Bonferroni adjustment.

To gain insight into the putative functions of the top associated loci, we queried the 81 genes identified by the MAGMA analyses in Gene Network [55], which predicts Reactome [56] functions for genes (Supplementary Information 6.2). In addition to neural functioning (e.g., axon guidance), we find that the MAGMA genes are predicted to be involved in growth factor signaling and the immune system (Supplementary Information 6.6). These results may imply a more pronounced role for peripheral gene functions than our stratified LDSC results, which mainly implicated the brain.

### Genetic correlations, heritability estimation, and polygenic prediction

We estimated pairwise genetic correlations between the macronutrients with bivariate LDSC [57]. All are statistically distinguishable from zero at *P* < 0.05 (except fat and protein), but also from one and negative one (Supplementary Information 7.1, Supplementary Information 7). (As we explain in Supplementary Information 2.8, negative phenotypic and genetic correlations are not mechanically induced by our phenotype definition.) Thus the macronutrients have overlapping but distinct genetic architectures, consistent with previous work from animal studies showing distinct biological mechanisms involved in macronutrient-specific appetites [48].

**Extended Data Figure 7 ∣:**
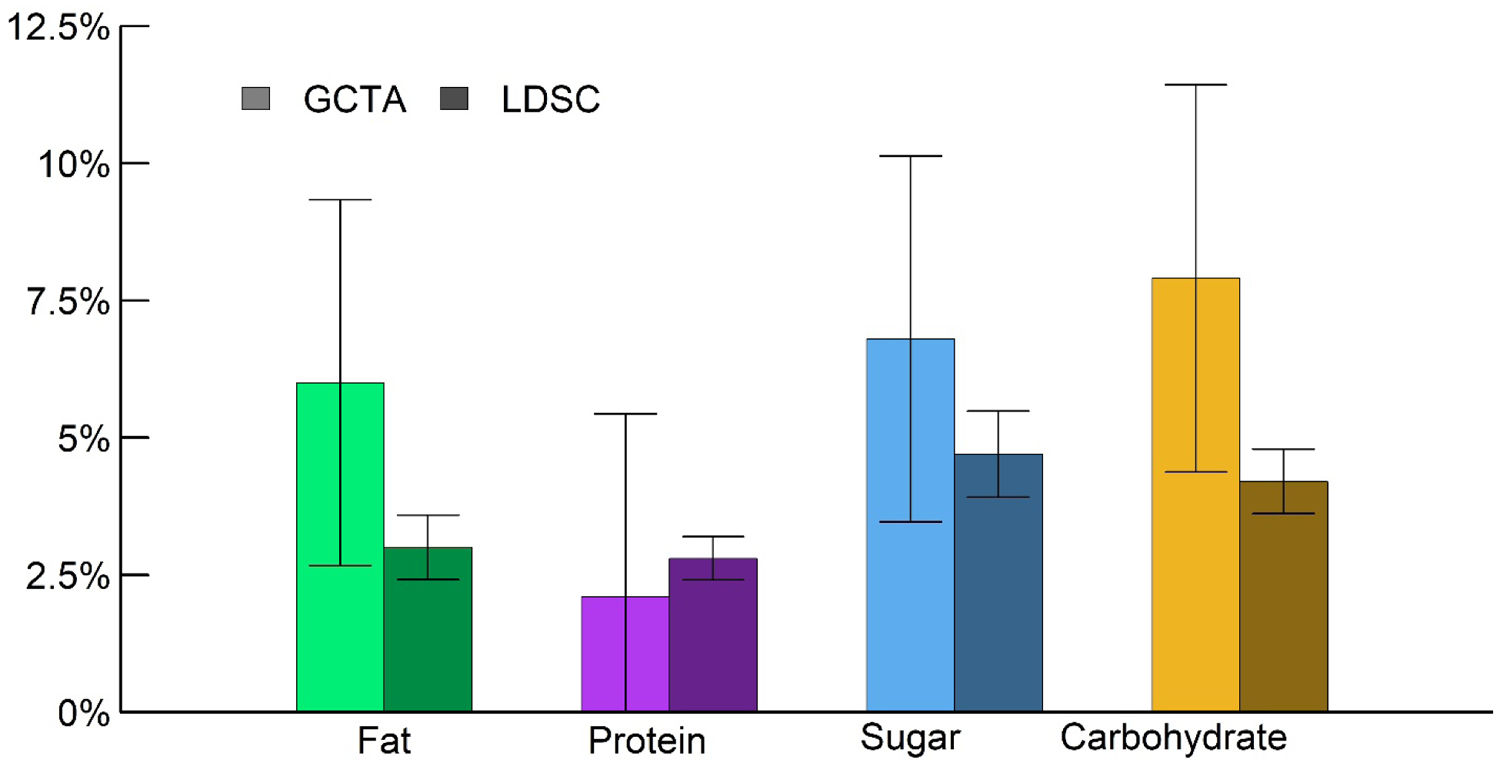
SNP-based heritability estimates of diet composition. SNP heritability was estimated with GCTA-GREML and LD Score regression. GREML was estimated using a random draw of 30,000 individuals from the UKB GWAS sample, from which we excluded cryptically related individuals, and using all genotyped SNPs with MAF > 0.01. LD Score heritability was estimated using HapMap3 SNPs with MAF > 0.01. See Supplementary Information 7 and Supplementary Table 7.1 for additional details.

We calculated GREML [28] estimates of SNP-based heritability using a random *N* = 30,000 subsample of conventionally unrelated UKB participants. The estimates range from 2.1% for protein to 7.9% for carbohydrate (Extended Data Fig. 7 and Supplementary Table 8.1). Our estimates are similar to previous estimates [20, 21]. These heritability estimates might be biased downward due to phenotypic measurement error (Supplementary Information 8.2) and are similar in magnitude to those from other complex (and also noisily measured) behavioral phenotypes, such as subjective wellbeing [34] and risk preferences [35].

**Extended Data Figure 8 ∣:**
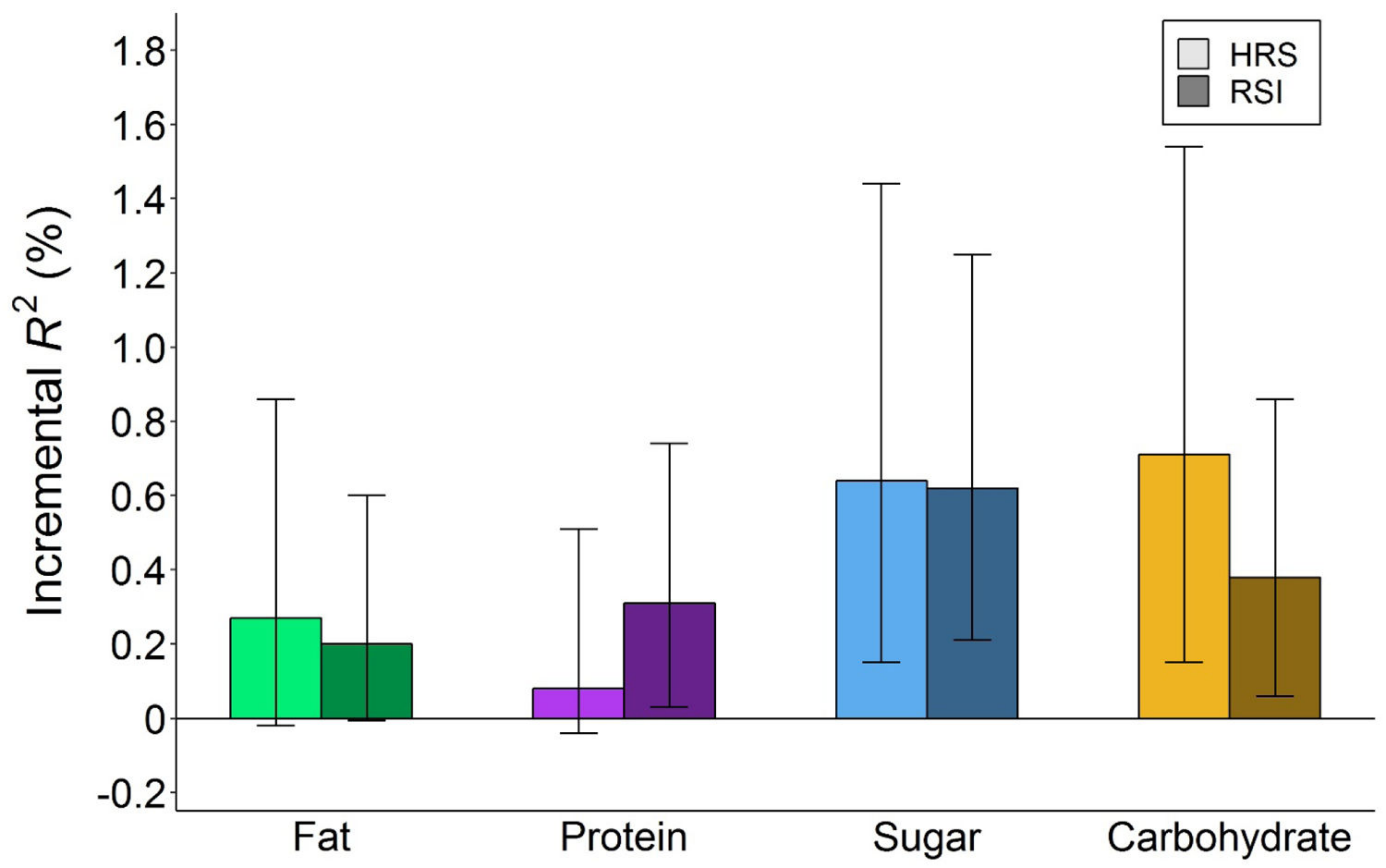
Out-of-sample prediction estimates of diet composition with polygenic scoring. Incremental *R^2^* is defined as the increase in *R^2^* from adding the polygenic score for the focal macronutrient to a regression of the macronutrient on control variables for sex, age, sex × age, and the top ten principal components of the genotype matrix. The polygenic scores were constructed using LDpred with Gaussian mixture weight of 1. The validation cohorts are the HRS and RSI cohorts. Supplementary Information section 8 and Supplementary Table 8.1 for additional details.

We constructed polygenic scores for the macronutrient intakes by applying LDpred [30] to our GWAS summary statistics. We assessed the scores’ out-of-sample predictive accuracy in two holdout cohorts: The Health and Retirement Study (*N* = 2,344) and the Rotterdam Study (*N* = 3,585). The scores predict the macronutrient intakes with incremental adjusted *R*^2^ ranging between 0.08% (*P* = 0.088) and 0.71% (*P* = 9.11 × 10^−7^; Supplementary Table 9.1, Extended Data Fig. 8).

### Relationships with health, lifestyle, and socioeconomic status

Using bivariate LDSC [57, 58], we estimated genetic correlations between our diet-composition phenotypes and 19 preselected relevant medical and lifestyle phenotypes for which well-powered GWAS results were available. We also included four additional phenotypes for which GWAS results became available after our study was underway, as well as Alzheimer’s disease, motivated by the association we found between *APOE* and macronutrient intakes, and nine phenotypes from the psychiatric domain. To control for multiple testing, we again used Bonferroni-adjusted *P*-value thresholds (*α* = 0.05/33).

**Extended Data Figure 9 ∣:**
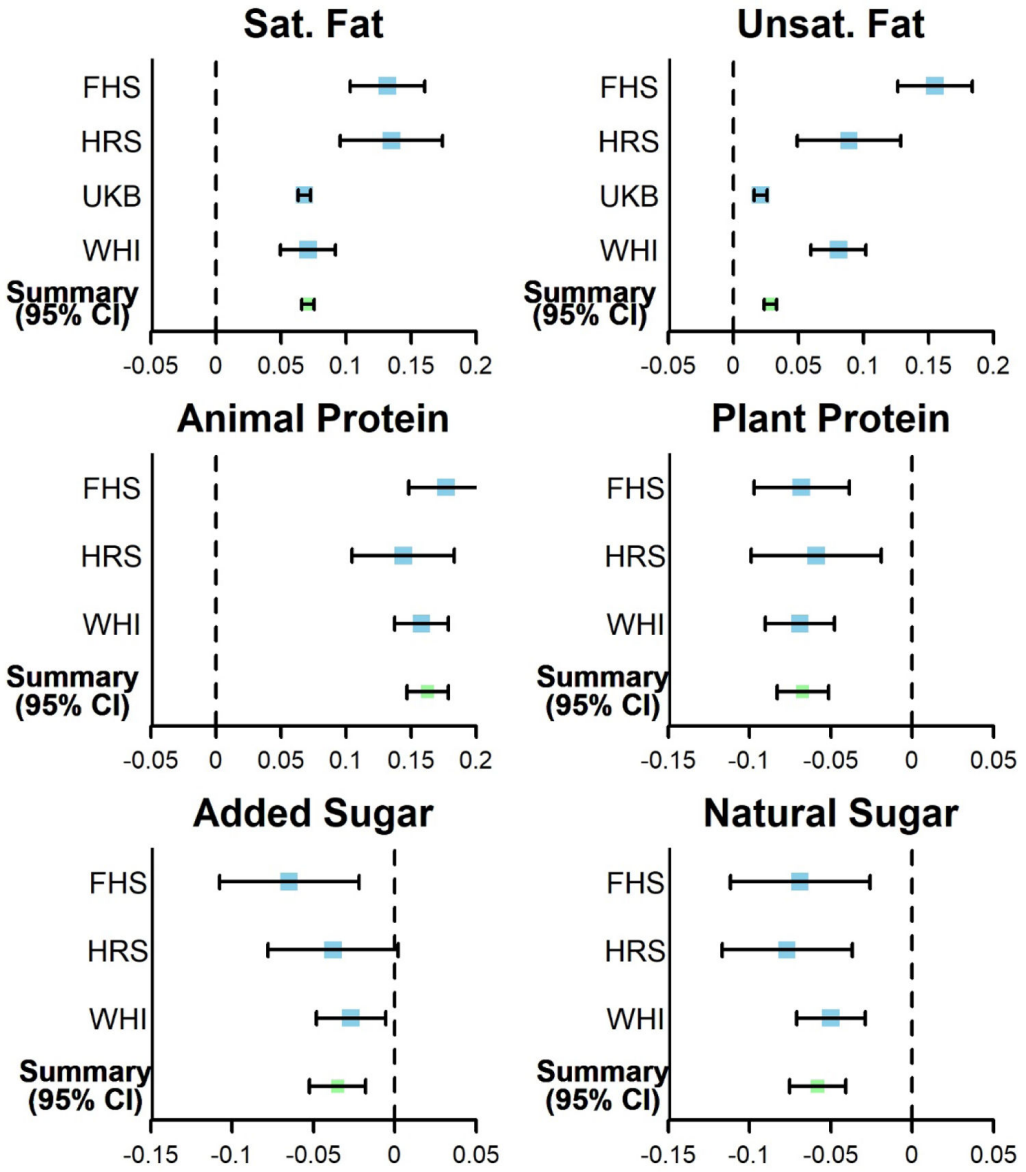
Phenotypic associations between macronutrient subtypes and Body Mass Index (BMI). Phenotypic associations are displayed in terms of standardized regression coefficients (with 95% confidence intervals). These coefficients were obtained from a regression of BMI on the focal macronutrient and several covariates (sex, age, educational attainment, and household income). FHS = Framingham Heart Study (*N* = 4,428), HRS = Health and Retirement Study (*N* = 2,385), UKB = UK Biobank (*N* = 157,852), WHI = Women’s Health Initiative (*N* = 8,500). The summary estimate was based on fixed-effects, inverse-variance-weighted meta-analysis.

**Fig. 3:**
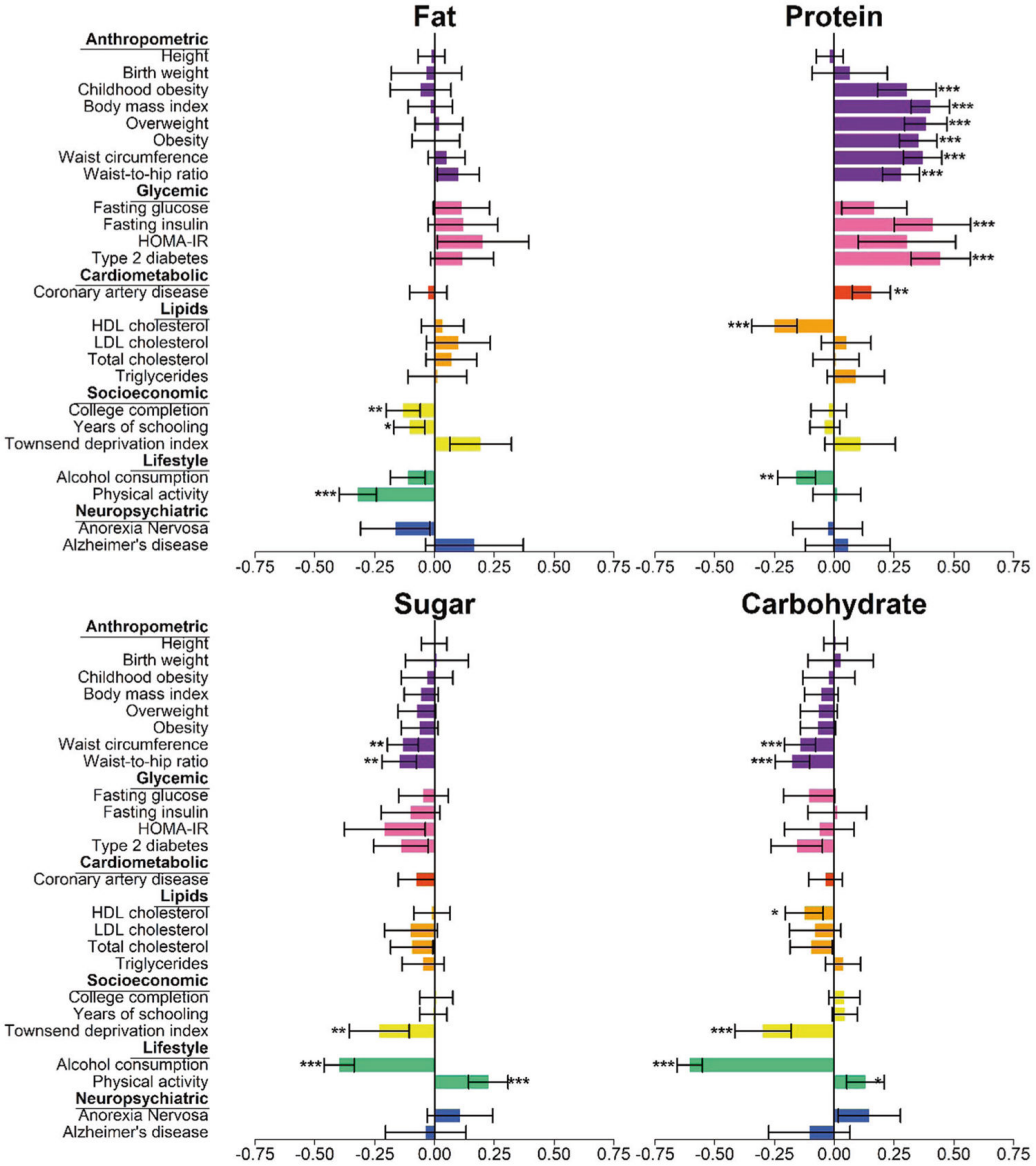
Genetic correlations. Genetic correlations were estimated with bivariate LD Score (LDSC) regression. Error bars show 95% confidence intervals, while asterisks denote Bonferroni-corrected *P*-value thresholds (*P/33 < 0.05, ** < 0.01, *** < 0.001), corrected for 33 traits. The colours represent the different functional domains.

**Fig. 4:**
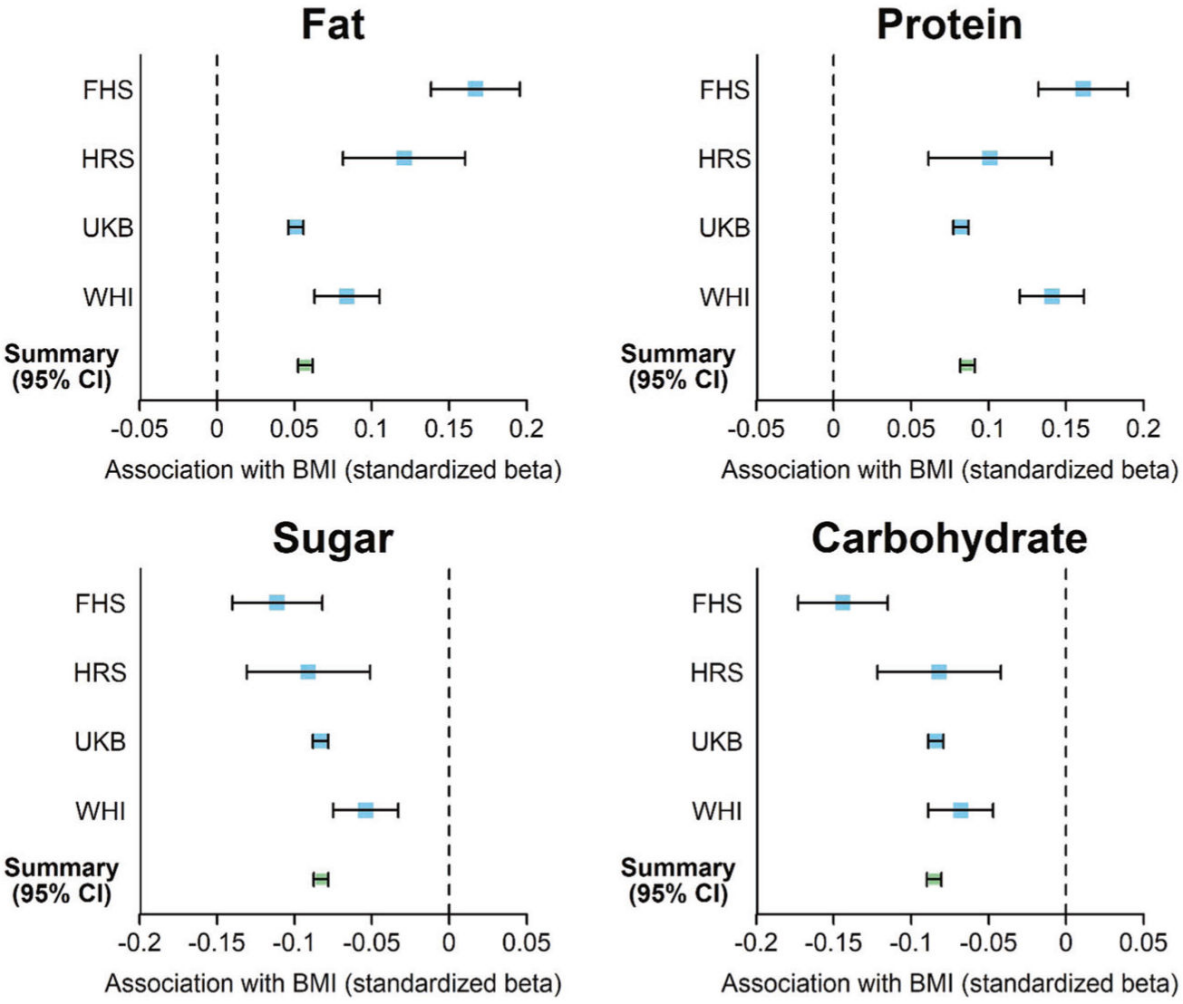
Phenotypic associations with body mass index. Phenotypic associations between diet composition and body mass index (BMI) in four independent cohorts, in terms of standardized regression coefficients (with 95% confidence intervals). These coefficients were obtained from a regression of BMI on the focal macronutrient and several covariates (sex, age, educational attainment, and household income). FHS Framingham Heart Study (*N* = 4,413), HRS Health and Retirement Study (*N* = 2,394), UKB UK Biobank (*N* = 158,046), WHI Women’s Health Initiative (*N* = 8,628). The summary estimate was based on fixed-effects, inverse-variance-weighted meta-analysis.

Protein exhibits the strongest genetic correlations with poor health outcomes, including obesity (*r_g_* = 0.35, *SE* = 0.04), type 2 diabetes (*r*_g_ = 0.45, *SE* = 0.06), fasting insulin (*r_g_* = 0.41, SE = 0.08), and coronary artery disease (*r_g_* = 0.16, *SE* = 0.04), as well as BMI (*r_g_* = 0.40, *SE* = 0.04) (Fig. 3, Supplementary Table 10.2). Fat, sugar, and carbohydrate has negative, non-significant genetic correlations with BMI (*r_g_* between −0.06 and −0.02). For comparison, we estimated phenotypic associations between diet composition and BMI in four independent cohorts (combined *N* = 173,353) and meta-analyzed the results (Fig. 4). Protein (standardized $\hat{\beta }=0.090$, 95% CI [0.085, 0.094]) and fat (standardized $\hat{\beta }=0.069$, 95% CI [0.059, 0.067]) are positively associated with BMI, while sugar and carbohydrate are negatively associated with BMI (standardized $\hat{\beta }$ = −0.082, 95% CI [−0.087, −0.078]; and −0.084, 95% CI [−0.088, −0.079] respectively, Supplementary Table 11.1). Thus, the genetic correlation between protein and BMI stands out as large relative to the phenotypic correlations. The phenotypic association between overall protein intake and BMI is probably driven by animal protein, which has a positive correlation with BMI (standardized $\hat{\beta }=0.16$, 95% CI [0.15, 0.18]), while plant protein has a negative correlation between BMI (standardized $\hat{\beta }$ = −0.07, 95% CI [−0.08, −0.05]). These protein subtypes were available in four population cohorts with a total *N* = 15,347. No such large differences are found between natural vs. added sugar and saturated vs. unsaturated fat (Supplementary Table 11.2, Extended Data Fig. 9).

Despite their relatively weak genetic correlations with BMI, sugar, and carbohydrate have negative genetic correlations with waist circumference (*r_g_* = −0.13, *SE* = 0.03, and *r_g_* = −0.14, *SE* = 0.03) and waist-hip ratio (*r_g_* = −0.15, *SE* = 0.04, and *r_g_* = −0.18, *SE* = 0.04) that are larger in magnitude and statistically distinguishable from zero at the 5% level. All the macronutrients have negative genetic correlations with alcohol consumption (*r_g_* between −0.61 and −0.11), as expected since alcohol is included in energy intake and our phenotype measures are shares of energy intake (Supplementary Information 2.8).

Next, we computed genetic correlations with indicators of socioeconomic status [25, 59, 60], which are heritable [59, 60] and known to be phenotypically associated with food access, dietary choices, and health [61-65]. We found that fat is negatively genetically correlated with educational attainment (*r_g_* = −0.13, *SE* = 0.04). Sugar and carbohydrate are negatively genetically correlated with the Townsend deprivation index (*r_g_* = −0.23, *SE* = 0.06 and −0.30, *SE* = 0.06), which is constructed from the rates of unemployment, non-ownership of cars and houses, and neighborhood overcrowding [60, 66], with higher scores indicating more severe socioeconomic deprivation. These genetic correlations might hint at environmental factors involved in macronutrient intake, although these relationships might also be caused by unmeasured, confounding factors.

Finally, we estimate the genetic correlations between diet composition and physical activity, which has widespread physical and mental health benefits [67, 68]. In these genetic correlation analyses, we used unpublished physical activity GWAS summary statistics from a sample of research participants from 23andMe (*N* = 269,189). The physical activity phenotype is a composite measure based on self-reported activities from leisure, occupation, and commuting. We find a negative genetic correlation of physical activity with fat (*r_g_* = −0.32, *SE* = 0.04) and a positive genetic correlation with sugar (*r_g_* = 0.23, *SE* = 0.04) and carbohydrate (*r_g_* = 0.13, *SE* = 0.04). The genetic correlation with protein is positive but not statistically distinguishable from zero at *P* < 0.05 (*r_g_* = 0.011). In the psychiatric domain, we find negative genetic correlations between saturated fat and schizophrenia (*r_g_* = −0.13, *SE* = 0.04) and between carbohydrate and ADHD (*r_g_* = −0.19, *SE* = 0.04). The negative genetic correlation with schizophrenia contrasts with its known positive phenotypic correlation, as patients with schizophrenia tend to consume higher amounts of saturated fat [69]. The negative genetic correlation with ADHD might be related to ADHD’s responsiveness to dietary intervention [70], or might be explained by socioeconomic status.

## Discussion

The genetic correlations we find between protein and obesity, waist-hip ratio, fasting insulin, type 2 diabetes, HDL cholesterol, and heart disease, together with the association we find between the BMI-increasing *FTO* allele and increased protein intake, point to an intriguing hypothesis: relative protein intake may play a role in the etiology of metabolic dysfunction. This hypothesis coincides with a growing (but often overlooked [71]) body of evidence that links protein intake to obesity and insulin resistance [72-80]. There is some related evidence from randomized trials with infants, which found a causal relationship between high-protein baby formula and infant body fat [81]. While the underlying biological mechanisms are unclear, high consumption of protein or certain types of amino acids (i.e., building blocks of protein) is known to induce insulin resistance [82-84], rapamycin signaling [77], and growth factor signaling [85], which might increase metabolic dysfunction and early mortality risk. Indeed, a recent phenotypic meta-analysis of prospective observational studies (pooled *N* = 154,344) found that low carbohydrate diets, which restrict carbohydrate in favor of increased animal protein or fat intake, were robustly associated with increased mortality [86].

We caution, however, that the strong and consistent links between protein and poor health outcomes might also be consistent with alternative explanations. Causation could run in the reverse direction: overweight individuals may have higher protein needs or use high-protein diets as a weight-loss strategy. The associations might also be caused by other, unmeasured variables such as unhealthy lifestyle factors or co-consumed ingredients. However, we find that the phenotypic association between protein and BMI is robust to controls for educational attainment and household income. Furthermore, the genetic correlation between protein and physical activity is statistically indistinguishable from zero. These findings weigh against socioeconomic status or physical activity being confounders of the positive genetic correlation between protein and BMI. In any case, the consistent associations that we find between protein intake and poor health warrant further attention.

For sugar, the phenotypic and genetic correlations we found with BMI and other health outcomes are consistent with observations from systematic reviews and meta-analyses of phenotypic relationships. These correlations may suggest that dietary sugar, beyond its energy content, does not have negative health effects [87-90], contrary to some popular beliefs (e.g., [91]). Another possibility is that exercise offsets negative metabolic effects of high sugar intake [92, 93]. Those with a higher predisposition to be physically active may tend to consume more sugar, as sugar is a metabolically convenient source of energy during exercise [94] and may enhance endurance [95]. If so, the positive genetic correlation between sugar and physical activity might partially explain the lack of genetic correlations between sugar and poor health.

For fat and carbohydrate, we also find no consistent pattern of genetic and phenotypic associations with poor metabolic health. Taken together, our results complement the findings of phenotypic analyses by the multinational EPIC-PANACEA consortium (pooled *N* = 373,803), which found that only calories from protein are associated with prospective weight gain [96]. While this finding was consistent across 10 countries, we caution that EPIC-PANACEA’s evidence, like ours, is limited by its reliance on self-reported eating habits.

Overall, our results show that the relative intake of each macronutrient has a distinct genetic architecture, and the pattern of genetic correlations might be suggestive of health implications beyond total calorie intake. Moreover, our genetic correlation and bioinformatics analyses suggest a number of novel hypotheses regarding the causes and consequences of dietary intake that can be explored in future work.

## Extended Data

## Supplementary Material

Supplementary Information

Supplmentary Table

## Figures and Tables

**Table 1:** Diet composition lead SNPs.

Top hit in locus for	SNPID	CHR	BP	Effect allele	Beta	*P*-value	Nearest gene
Protein	rs780094	2	27,741,237	t	0.018	5.58E-10	*GCKR*
Sugar	rs12713415	2	60,205,134	c	−0.019	4.88E-09	*AC007100.1*
Carbohydrate	rs10206338	2	60,209,981	a	−0.016	1.52E-08	*AC007100.1*
Protein	rs445551	2	79,697,982	a	0.019	1.49E-08	*CTNNA2*
Carbohydrate	rs10510554	3	25,099,776	t	0.019	2.94E-12	*AC133680.1*
Protein	rs1603978	3	25,108,236	a	0.019	1.35E-10	*AC092422.1*
Sugar	rs7619139	3	25,110,415	a	−0.024	4.98E-16	*AC092422.1*
Carbohydrate	rs10433500	3	85,546,798	a	0.016	1.96E-08	*CADM2*
Protein	rs13146907	4	39,425,248	a	−0.022	1.24E-14	*KLB*
Fat	rs1229984	4	100,239,319	t	0.098	2.64E-28	*ADH1B*
Sugar	rs13202107	6	51,395,463	a	−0.020	1.77E-08	*SNORD66*
Fat	rs57193069	7	1,862,417	a	−0.016	1.80E-08	*MAD1L1*
Carbohydrate	rs7012637	8	9,173,209	a	0.017	4.68E-10	*AC022784.6*
Fat	rs7012814	8	9,173,358	a	−0.019	1.12E-11	*AC022784.6*
Sugar	rs7012814	8	9,173,358	a	0.019	4.99E-10	*AC022784.6*
Carbohydrate	rs9987289	8	9,183,358	a	−0.026	4.64E-08	*AC022784.6*
Protein	rs1461729	8	9,187,242	a	0.032	4.09E-12	*AC022784.6*
Carbohydrate	rs10962121	9	15,702,704	t	−0.015	3.40E-08	*CCDC171*
Carbohydrate	rs2472297	15	75,027,880	t	−0.018	3.73E-08	*CYP1A1*
Protein	rs55872725	16	53,809,123	t	0.018	2.09E-10	*FTO*
Sugar	rs9972653	16	53,814,363	t	−0.020	1.53E-11	*FTO*
Fat	rs9927317	16	53,820,996	c	−0.024	4.77E-12	*FTO*
Carbohydrate	rs7190396	16	53,822,502	t	0.018	2.39E-10	*FTO*
Carbohydrate	rs1104608	16	73,912,588	c	0.018	1.74E-10	*AC087565.1*
Carbohydrate	rs36123991	17	44,359,663	t	0.021	8.24E-09	*ARL17B*
Sugar	rs8097672	18	1,839,601	a	0.030	1.54E-12	*AP005230.1*
Carbohydrate	rs8097672	18	1,839,601	a	0.023	1.95E-09	*AP005230.1*
Sugar	rs341228	18	6,395,336	t	0.019	2.72E-09	*L3MBTL4*
Fat	rs429358	19	45,411,941	t	0.024	8.65E-10	*APOE*
Sugar	rs429358	19	45,411,941	t	−0.028	2.97E-11	*APOE*
Carbohydrate	rs429358	19	45,411,941	t	−0.027	3.49E-12	*APOE*
Fat	rs33988101	19	49,218,111	t	−0.029	1.66E-26	*MAMSTR*
Sugar	rs838144	19	49,250,239	t	−0.028	8.53E-21	*IZUMO1*
Carbohydrate	rs838144	19	49,250,239	t	−0.023	3.26E-17	*IZUMO1*
Protein	rs838133	19	49,259,529	a	−0.032	4.52E-26	*FGF21*
Sugar	rs62132802	19	49,270,872	t	−0.020	1.07E-08	*FGF21*

GWAS summary statistics of the 36 diet composition lead SNPs. A total of 21 of these lead SNPs are approximately independent. Supplementary Table 5.1 reports the effect alleles and summary statistics across all four phenotypes for each individual lead SNP. MAF = minor allele frequency (weighted average across cohorts). Beta = increase in phenotypic standard deviations per effect allele. All *P*-values are calculated using standard errors that have been inflated by the estimated LDSC intercept.
