# Supplementary material for: Genomic analysis of diet composition finds novel loci and associations with health and lifestyle
Source: Mol Psychiatry. 2020 May 11;26(6):2056–69. doi: 10.1038/s41380-020-0697-5 (PMC7767645; doi:10.1038/s41380-020-0697-5)
Supplement: Supplementary file 1 — Supplementary information [file 41380_2020_697_MOESM1_ESM.pdf]

Supplementary Information

Genomic analysis of diet composition finds novel loci and associations with health and lifestyle

Correspondence to:
s.f.w.meddens@vu.nl, carsonc@niddk.nih.gov,
daniel.benjamin@gmail.com, p.d.koellinger@vu.nl

Contents

1 COHORTS 5

1.1 Overview 5
1.2 Discovery phase 5
1.3 Replication phase 6

2 PHENOTYPE DEFINITION 6

2.1 Overview 6
2.2 Background 7
2.3 Sugar intake 7
2.4 Note on misreporting 8
2.5 Dietary intake in the UKB 10
2.6 Phenotype definition 12
2.7 Comparison to regular macronutrient densities 14
2.8 Interpretation 15
2.9 Correction for dieting 17

3 GWAS, QUALITY CONTROL AND META-ANALYSIS METHODS 17

3.1 Genotyping and imputation 17
3.2 Association analyses 18
3.3 Quality control 22
3.4 Meta-analysis 24

4 REPLICATION 25

4.1 Genetic correlations between discovery and replication cohorts 25
4.2 Replication of GWAS hits from the discovery stage 27
4.3 Replication of *DRAM1* variant rs77694286 31

5 GWAS RESULTS FROM THE COMBINED META-ANALYSES 32

5.1 LD Score regression intercept test for population stratification bias 32
5.2 Summary of the GWAS findings 33
5.3 MAGMA gene-based analysis 37
5.4 Overlap between loci 38
5.5 Overlap with traits in the NHGRI-EBI GWAS catalog 39

6 BIOLOGICAL ANNOTATION OF GWAS FINDINGS 40

6.1 LD Score partitioning of heritability 41
6.2 Annotation of genome-wide significant genes and loci 44
6.3 Discussion of bioannotation results 48

7 GENETIC CORRELATIONS BETWEEN THE PHENOTYPES AND SEXES 49

7.1 Genetic correlations between macronutrients 49
7.2 Genetic correlations between sexes 50

8 ESTIMATION OF SNP-BASED HERITABILITY 50

8.1 Methods 51
8.2 Results and discussion 51

9 OUT-OF-SAMPLE PREDICTION 52

9.1 Method 52
9.2 Prediction model and prediction accuracy 53
9.3 Results 53
9.4 Discussion 54

10 GENETIC CORRELATIONS WITH HEALTH AND BEHAVIOR 55

10.1 Method 56
10.2 Association with psychiatric traits 56
10.3 Results 57
10.4 Discussion 59

11 PHENOTYPIC ASSOCIATIONS BETWEEN DIET COMPOSITION AND BMI, SES, AND ALCOHOL ABSTINENCE 61

11.1 Method 62
11.2 Results 64

12 CONTRIBUTIONS 67

12.1 Author contributions 67
12.2 Cohort contributions 67

13 ADDITIONAL ACKNOWLEDGMENTS 69

13.1 Author acknowledgements 69
13.2 Extended acknowledgements 70
13.3 Cohort acknowledgments 70

14 REFERENCES 73

List of abbreviations

24HDR	=	24-hour dietary recall
ARIC	=	Atherosclerosis Risk in Communities Study
BMI	=	body mass index
CI	=	confidence interval
eQTL	=	expression quantitative trait locus
FDR	=	false discovery rate
FFQ	=	food-frequency questionnaire
FHS	=	Framingham Heart Study
GCTA	=	genome-wide complex trait analysis
GREML	=	genomic-relatedness-matrix restricted maximum likelihood
GWA	=	genome-wide association
h^2	=	heritability
HRC	=	Haplotype Reference Consortium
HRS	=	Health and Retirement Study
HWE	=	Hardy-Weinberg equilibrium
Indel	=	insertion-deletion
Kb	=	kilobase
kCal	=	kilocalorie
kJ	=	kilojoule
MAF	=	minor allele frequency
Mb	=	megabase
OLS	=	ordinary least squares
PAL	=	physical-activity level
PC	=	principal component
PCA	=	principal component analysis
QC	=	quality control
RD	=	(calorie/macronutrient)-restricted diet
s.d.	=	standard deviation
S.E.	=	standard error
SNP	=	single-nucleotide polymorphism
SSGAC	=	Social Science Genetic Association Consortium
UKB	=	UK Biobank
WHI	=	Women's Health Initiative

1 Cohorts

1.1 Overview

Our discovery cohort is the UK Biobank (UKB). In our replication analyses (**Supplementary Information 4**), we test the credibility of the lead SNPs found in the discovery cohort. These replications—which we call our "replication phase"—are conducted as a meta-analysis of our 14 replication cohorts and (for PROTEIN, CARBOHYDRATE AND FAT) previously published summary statistics from DietGen.

For all other analyses, including polygenic prediction and bioannotation, we use the results from all cohorts meta-analyzed together: UKB, the 14 replication cohorts, and DietGen. We refer to these meta-analyses as our "combined meta-analyses". Our study design is summarized with a flow chart (**Extended Data Figure 2**).

An overview of all cohorts included in the combined meta-analysis is listed in **Supplementary Table 1.1**. A short overview of the dietary intake questionnaires, along with phenotypic summary statistics for cohort demographic variables and dietary intake estimates can be found in **Supplementary Table 1.2.** The cohort-specific exclusion criteria and genotyping and imputation filters can be found in **Supplementary Table 1.3**.

1.2 Discovery phase

Our discovery sample consists of individuals of European (white British, Scottish, Irish, or other European background) ancestry of the UKB. The UKB, which was established in 2006[1], is a large British general population cohort of individuals aged 45 to 69 years at recruitment. Individuals who were registered with the National Health Service and who lived in proximity to one of the 22 assessment centers were eligible for participation. In total, 9.2 million individuals received a mailed invitation, of whom $N = 503{,}325$ (response rate 5.4%) agreed to participate. All participants provided informed consent. When we were made aware of participants who withdrew consent, these participants were excluded from any new analyses.

The dietary-intake questionnaire in UKB was administered once at a UKB assessment center by use of a touchscreen. However, only 14.1% of participants completed this questionnaire at the assessment center, as it was added to the questionnaire battery later during the recruitment process. In addition, four invitations for an online version were sent to $N = 331{,}008$ UKB participants with a known email address. Across these internet surveys, an average 31.4% responded and completed the full survey. $N = 150{,}617$ completed at least one internet survey, which corresponds to 45.5% of individuals who were sent an invitation to participate. In total, 40% of the UKB sample ($N_{\text{24HDR}} = 211{,}063$ vs. $N_{\text{total}} = 502{,}643$) completed at least one dietary-intake questionnaire. Of these $N = 211{,}063$ individuals, $N = 175{,}253$ passed internal quality control (both at the genotypic and phenotypic level; see **Supplementary Table 3.3** for details) for GWAS.

1.3 Replication phase

The 14 replication cohorts consist of numerous cohorts from the Netherlands (Lifelines[2] and Rotterdam Study[3]), United Kingdom (ALSPAC[4,5], Fenland[6]), and United States (Framingham Heart Study[7], Health and Retirement Study[8], Women's Health Initiative[9]: GARNET, HIPFX, and WHIMS+), and from the international study consortium of EPIC-InterAct[10], which encompasses cohorts from France, Germany, Italy, Spain and Sweden. All participants in these cohorts are adults who provided informed consent. Recruitment of replication cohorts was based on a priori power calculations for replication of the polygenic score and individual SNPs in holdout cohorts. Our initial aim was to continue recruitment until we achieved the minimum sample size needed to replicate each phenotype's primary lead SNP with a one-sided alpha of 5% with at least 80% power. With the delayed second release of the UKB, however, we kept the recruitment of the replication phase open for longer, and we surpassed our original goal of $N \geq$ 141,000, which was needed to identify at least one locus at genome-wide significance with 80% power for each macronutrient (as detailed in our analysis plan https://osf.io/mt9kt/). In addition, we obtained access to the summary statistics from the CHARGE[11] and DietGen[12] consortia, which previously performed GWAS on percentage intake from protein, carbohydrate and fat. While this phenotype definition differs slightly from ours, we find large enough genetic correlations between DietGen and our replication phase, discovery phase, and combined replication and discovery phase to warrant meta-analysis (**Supplementary Information 4.1**). In the end, we decided to not include CHARGE in our replication phase, since the sample overlap between our replication cohorts and CHARGE cohorts turned out to be much larger than we expected.

Since the DietGen summary statistics only included HapMap2 SNPs, we impute the DietGen results to 1000 Genomes Phase 3 Version 2 EUR (information from 381 European individuals) using impG[13] software. In our quality control procedure for DietGen (**Supplementary Information 3.3**), we apply more stringent SNP filters compared to other cohorts of $N > 10{,}000$.

We also obtained access to the ARIC cohort via dbGaP (https://dbgap.ncbi.nlm.nih.gov). This cohort was not included in our meta-analysis since allele-frequency plots (comparing allele frequencies in the ARIC data with those in our reference panel) revealed a pervasive strand alignment issue that most likely arose from imputation, with probable large downstream effects.

2 Phenotype definition

2.1 Overview

In UKB and the 14 replication cohorts, we apply our novel, non-linear correction method for total reported energy intake. The DietGen summary statistics, however, were based on a correction (% of total energy intake) that assumes linear scaling between the macronutrients and total energy intake. Our novel correction method is described in detail below.

2.2 Background

We perform genome-wide association analyses for four dietary composition phenotypes, which together comprise our measure of diet composition:

1. Relative protein intake (PROTEIN)
2. Relative fat intake (FAT)
3. Relative carbohydrate intake (CARBOHYDRATE)
4. Relative sugar intake (SUGAR)

As an auxiliary analysis in the UKB, we also perform GWAS for saturated fat:

5. Relative saturated fat intake (SATURATED FAT)

These macronutrients[a] are corrected for total energy intake (**Supplementary Information 2.6**). This is to correct for *total* energy intake—which is hard to measure reliably[14,15], and which is mainly a function of body size and physical activity[16–18] (for further discussion see **Supplementary Information 2.7**). We are interested in studying the genetic architecture and genetic correlations of the relative intake of macronutrients. For instance, fat is the most energy-dense of all macronutrients, so fat intake may be associated with obesity only by way of its calorie count. However, if the association between fat and obesity remains after correction for total energy intake, this potentially implies that fat has health effects through other pathways – for instance through specific effects on atherosclerosis or endocrine signaling. We do not perform GWAS for alcohol (i.e., ethanol) intake even though it is another component of total energy intake, since it is a non-essential macronutrient with various complicated associations, and thus outside of the scope of this project.

2.3 Sugar intake

In all our GWAS cohorts (both discovery and replication), the total sugar intake variable used in phenotype construction is a subset of the total carbohydrate intake variable. Our definition of sugar intake constitutes intake from short-chain saccharides: mono- and disaccharides. The predominantly consumed monosaccharides are glucose, fructose, and galactose, and the predominantly consumed disaccharides are sucrose ("table sugar"; a disaccharide chain of glucose and fructose), and lactose ("milk sugar"; a disaccharide chain of glucose and galactose). Sugars naturally occur in fruits and dairy, are often added to food or drinks in the form of sucrose or fructose, and are sweet in taste. Non-sugar carbohydrates are longer chains of saccharides, known as oligo- and polysaccharides (e.g., starch and glycogen), which are less sweet in taste. Cellulose is also a polysaccharide but is an insoluble and non-digestible fiber, and therefore is a negligible component of energy intake.

The GWAS of carbohydrates includes intake from all saccharides, while the GWAS of sugar includes intake from mono- and disaccharides *only*. As noted above, this category includes not

---

[a] A macronutrient is a molecule that human organisms can derive energy from.

only added and refined sugar (found in for instance, sugar-sweetened beverages and candy), but also natural sugars, found mainly in fruit and dairy products.

An exception to this definition of the sugar phenotype was made for the two EPIC-InterAct cohorts, which only analyze sugars consumed as a single dietary intake of honey, syrup, jams, or other non-artificial sweeteners[19]. Participating cohorts of the InterAct conducted a dietary survey at baseline independently of each other, with cohort-specific dietary instruments, food frequency questionnaires or diet history[19,20]. Validity of dietary instruments was assessed in each cohort against 24-hour recalls, food diaries, or both[21]. Dietary variables were standardized across these cohorts as part of the multi-country epidemiological study of European Prospective Investigation into Cancer and Nutrition (EPIC). Food groups were harmonized across EPIC's participating cohorts and standardized by using EPIC-SOFT software, which managed country-specific descriptions such as meals, foods, ingredients, and cooking methods[19]. Consumption of refined sugars, one of the 39 food groups in the harmonized data, is used in the current analysis. In **Supplementary Information 4.1.4,** we report the genetic correlation between a meta-analysis of the two EPIC-InterAct cohorts and a meta-analysis of the remaining cohorts for SUGAR.

2.4 Note on misreporting

Misreporting of dietary intake is a well-known and pernicious problem in nutritional epidemiology. Misreporting is non-random, with overweight individuals tending to misreport relatively more kilocalories[15]. This BMI-correlated misreporting distorts correlations between self-reported estimates of total energy intake and weight gain[22]. This differential misreporting is said to give rise to the 'fundamental flaw of obesity research'[23]. For this reason, some researchers have called for abandoning self-reported total energy intake altogether[14], but at a minimum, correcting macronutrients for total consumed energy is considered crucial[24].

Correcting macronutrients for total reported energy is insufficient, however, when the extent of misreporting differs across macronutrients. However, it is difficult to estimate the extent of macronutrient-specific misreporting, as only protein intake has a valid and reliable biomarker (24-hour urinary nitrogen output)[25]. Measuring misreporting of the other macronutrients is possible when food intake of participants is monitored and scored externally, for instance in the setting of laboratory confinement[26,27] or with video-monitoring[28]. At the food-item level, snack foods[26,29] and foods that are considered unhealthy may be especially selectively underreported[30].

A meta-analysis of five US studies with biomarkers for total energy and protein intake found that higher BMI is correlated with *under*-reporting of *total* protein and energy intake, but with *over*-reporting of protein *density* (% protein of total energy intake, "protein density")[31]. This was also true in a Danish sample[32]. Hence, any associations between relative protein intake and BMI could be biased upward by this effect.

However, there are two reasons why protein-intake overreporting need not upwardly bias associations with BMI. First, Ankarfeldt et al.[33] analyzed the same Danish sample described above, and still found that *both* self-reported relative protein intake and an objective measure (i.e., urine-ascertained) of relative protein intake were associated with fat mass gain to a similar

extent. Hence, the association between self-reported protein intake and weight gain was not biased by over-reporting of relative protein intake. Using longitudinal data on macronutrient substitution (i.e., individual changes in macronutrient composition over time), they found that the effect of protein on weight gain was similar when protein was substituted over time for any other macronutrient (fat, carbohydrates, or alcohol). Hence, weight gain appeared to be driven by high protein intake, and not low carbohydrate or fat intake. This is the only study we could find that compared self-reported to objective protein intake in relation to weight gain or BMI. Second, the construction of relative protein intake (i.e., protein density) by these studies[31–33] tends to leave a negative correlation with total energy intake, as we show in **Supplementary Information 2.7**. This could lead to a downward bias in associations between protein density and BMI, as individuals with a higher protein density tend to consume fewer calories in total. This downward bias could cancel out the upward-biasing effect of overreporting. Thus, while we believe that macronutrient-specific misreporting might be an issue, it is unknown how it may affect associations with BMI and its related predictors and outcomes.

Some might argue that macronutrient-specific misreporting can be addressed by excluding individuals most likely to misreport total energy intake. The assumption here is that extreme underreporters of total energy are also more likely to overreport their protein intake. These individuals are also more likely to be overweight. Excluding these individuals should then leave a sample of plausible reporters. A common exclusion criterion is referred to as the Goldberg cut-off[34], which effectively excludes individuals with implausible physical activity levels. These physical activity levels are not reported by individuals but are implied by the ratio of the individual's predicted basal metabolic rate to the individual's total reported energy intake[22]. According to this approach, an individual should be excluded from all analyses when their implied physical activity level is extremely deviant (e.g., +/- 2 standard deviations removed from a population average). Thus, individuals with extremely high physical-activity levels are deemed probable over-reporters, while individuals at the other end are deemed probable under-reporters.

However, we believe that excluding individuals based on extreme reports of energy intake is undesirable for several reasons. First, this Goldberg exclusion creates a selection bias, whereby overweight individuals are disproportionately more likely to be removed from the sample[22,35], which can induce spurious associations. In the worst case, collider bias is introduced, where associations have the wrong direction of association. Second, the Goldberg exclusion does not take into account individual levels of physical activity and may thus erroneously exclude individuals who in reality are at the extreme ends of the physical activity level distribution. At the same time, it may erroneously retain individuals whose actual physical activity levels do differ from their implied physical activity levels, but whose implied physical activity levels still fall within the population confidence bounds.

Hence, we do not believe that performing additional analyses that exclude individuals according to a Goldberg(-like) cutoff are useful in our case. However, some cohorts did exclude individuals with extremely high or extremely low self-reported total energy intake (**Supplementary Table 1.3**), as a strategy to remove a small set of extreme outliers that might bias regression estimates.

2.5 Dietary intake in the UKB

2.5.1 Short description

The estimates of total macronutrient intakes we use were derived by UKB and expressed in grams. The energy values of the macronutrients are obtained using the conversion factor of 4 kcal/gram for protein, sugar and carbohydrate, and 9 kcal/gram for fat. We use kilocalories for convenience, although units are irrelevant as long as they are consistent. Dietary intakes were obtained by UKB on the basis of responses from a computerized 24-hour dietary recall (24HDR) questionnaire called "Oxford WebQ", which was designed using repeated testing to ensure comparability to interview-based measures[36,37]. The macronutrient and energy intakes were derived by UKB from the information recorded in McCance and Widdowson's "The Composition of Food" and its supplements*,* fifth edition[38], taking into account sex-specific portion sizes and other factors. An example of the Oxford WebQ can be found online[b].

In UKB, participants were successively presented with lists of food items designed to capture the eating habits of the British population and asked how many portions (if any) of each food item they ate in the previous day. Individuals were not allowed to proceed to the next list of food items if they had missing answers. The questionnaire contained over 200 food items.

Dietary intakes derived from 24HDR questionnaires exhibit more random variation than those derived from, for instance, the widely used food-frequency questionnaire (FFQ), in which participants are asked to report their intakes for *a typical day* in the last week or month. This random variation can be averaged out as more 24HDR questionnaires are completed. However, dietary intakes from FFQs may be more strongly biased by systematic underreporting of total energy intake, and misreporting of foods that are considered unhealthy[39,40]. Researchers sometimes attempt to quantify these measurement errors by comparing intakes from repeated 24HDR measurements to those from FFQs[41]. FFQs are used by all of our replication cohorts.

2.5.2 Correction for weekend intake

As noted earlier, a key difference between 24HDR- and FFQ-estimates of dietary intake is that the former method intends to measure intake during the previous day, while FFQ intends to measure habitual dietary intake (i.e., on a "typical" day). As such, 24HDR-data require a correction for day of the week that the assessment was performed for. In UKB, data for both weekend and weekdays are available: the first 24HDR assessment in UKB took place at the UKB assessment centers, which was open on Monday through Saturday (and on rare occasions on Sundays). The additional four email invitations were sent out on different days of the week in each cycle.

It is important to note that not all UKB participants completed the questionnaire for *both* a weekday and a weekend-day, which hampers the implementation of a simple weighting of weekend days (i.e., 2/7 weight for weekend days and 5/7 weight for weekdays). This inability to account for weekend data is potentially problematic, as dietary intake differs in the weekend,

---

[b] URL: http://www.ceu.ox.ac.uk/research/web-based-questionnaires.

with higher total energy and alcohol intake, and relatively higher protein and fat intake[42,43]. Hence, we may underestimate intakes for individuals who only have weekday reports.

Our approach is to apply an empirical "day-of-the-week" weight to the *total* intakes of energy, fat, protein, carbohydrate, and sugar. We take into account that UKB asked participants to report on their dietary intake for the preceding day. We regress the total intakes on dummies for all weekend- and weekdays, excluding Wednesday, which thus serve as the "reference" category in each regression. We do this separately for each of the five intake estimates (i.e., total calories; protein; fat; carbohydrates; and sugar), and for each of the five measurement rounds, giving rise to 5×5 = 25 regressions. Here, we find that Fridays are empirical "weekend days" in terms of dietary intake, while Monday, Tuesday and Thursday are regular weekdays in the sense that they do not differ from Wednesday. Individuals tended to consume more total energy on Friday, Saturday and Sunday. They also tended to eat less carbohydrates and sugar, and more protein and fat on these weekend days.

In each of these 25 regressions, we store the six regression coefficients (one for all days except our baseline day, Wednesday), and then compute the regression residuals. To illustrate, in the first measurement, the estimated coefficient of total energy intake on the day-of-the-week dummies is 359 for Saturday (i.e., $\hat{\beta}_{saturday}$). Hence, the average UKB participant consumed 359 kilocalories more on Saturday than on Wednesday. If an individual reported dietary intake for a Saturday in that measurement round, 359 kilocalories would thus be subtracted from that person's total estimated energy intake. Of note, we apply these regression-based corrections to each day of the week, and not just to the weekend days. After correcting the total intakes in each measurement round, we continue with our phenotype construction as usual: we compute the average corrected intakes across the five measurement rounds, and use these for the log-log regressions described in **Supplementary Information 2.6.**

Supplementary Table 2.3 shows the differences between the average total intake estimates and the corrected average total intake estimates in genotyped individuals of European ancestry from the first release of the UKB. While the differences are small, all are statistically significant in paired *t*-tests. However, we did find that these weekday-corrected phenotypes correlated almost perfectly with the non-corrected phenotypes (ranging between r = 0.991 and r = 0.998), indicating that a correction for weekend is not strictly necessary, but still useful to increase measurement precision, and, therefore, statistical power.

2.5.3 Test-retest reliability

As noted earlier (**Supplementary Information 2.5.1**), UKB participants have up to five 24HDR measurements. Due to random fluctuation in day-to-day eating habits and episodic consumption of certain foods or nutrients (e.g., fish, dark green vegetables)[44], imperfect correlation between measurement rounds is expected. Therefore, true "habitual" intake is usually envisioned as long-term average intake[44]. Random measurement noise will bring a downward bias to estimated coefficients that cannot be alleviated with larger sample sizes.

Among the number of individuals that are included in the GWAS with 24HDR data, N = 64,910 have 1 observation; N = 40,926 have 2 observations; N = 37,214 have 3 observations; N = 27,023

have 4 observations; and N = 5,180 have 5 observations. The repeated observations allow us to calculate test-retest reliability estimates for the four macronutrients. We use Cronbach's alpha (denoted with α) as a measure of consistency across measurements in time. We perform the log-log regressions (**Supplementary Information 2.6**) on the weekend-corrected total macronutrient intakes (**Supplementary Information 2.5.2**) for each measurement cycle, and apply listwise deletion of observations. This means that we retained all information on all available measurements, as the majority of participants did not have the full set of five observations.

The results are displayed in **Supplementary Table2.2**. Given the natural daily fluctuation in eating habits, we expect the macronutrient composition to not be perfectly stable across measurements. The lowest reliability is found for protein ($\alpha = 0.60$) and the highest reliability for sugar ($\alpha = 0.79$). Fat ($\alpha = 0.69$) and carbohydrate ($\alpha = 0.76$) are in the middle. The rank-order of reliability estimates corresponds to the rank-order of heritability estimates for the macronutrients, with relative protein intake being the least heritable, and relative sugar and carbohydrate intake being the most heritable (**Supplementary Information 8)**. The results show that self-reported consumption of relative sugar and carbohydrate intake is especially stable, while relative fat and protein intake might be more subject to random day-to-day fluctuations or recalled with less accuracy.

2.6 Phenotype definition

Because we are interested in the identification of genetic variants associated with diet *composition*, we seek to correct the macronutrient intakes for reported total energy intake. A standard approach is to divide macronutrient intake by total energy intake, resulting in intake fractions which are often referred to as "macronutrient densities"[18]. However, if relative macronutrient intake does not scale linearly with total energy intake, the construction of simple macronutrient proportions may not represent the optimal correction for total energy intake. The macronutrient intakes would then not be properly corrected for total energy intake, leaving residual correlations between macronutrient- and total energy-intake, which potentially differs by macronutrient. This correlation could bias any downstream associations related to diet composition differentially for the macronutrients. Since total energy intake is mainly a function of body size and physical activity[16–18], this can also bias associations with BMI and related health outcomes. Using a ratio as an outcome variable may induce spurious correlations between the ratio and the denominator. This is a well-known problem of crude ratios that was noted by Pearson over a century ago[45]. We alleviate this concern by correcting the denominator for an empirical "correction" factor, β, as detailed below.

Keeping these concerns in mind, our own phenotype definition of the energy-corrected macronutrient intakes is given by

$$corrected\ intake = \frac{energy\ from\ macronutrient}{total\ energy^{\beta}}$$

which could be described as an "adjusted macronutrient density". Here, the macronutrient- and total energy intake have to be measured in the same unit (e.g., kilocalories). β is a correction factor that is estimated with the procedure described below.

The simple macronutrient density (i.e., percentage intake) implicitly assumes β to be equal to one. However, it is possible to *estimate* the value of β with a multiplicative regression model. We estimate β separately for each macronutrient j, where the reported macronutrient intake (in kcal) is regressed on reported energy intake (in kcal). The estimation of β_j is achieved by means of a log-log regression, which transforms the multiplicative model into a simple linear regression model. More specifically, the multiplicative model for individual i and macronutrient j is given by the generic form below:

$$y_i = \alpha {x_i}^{\beta_j} \varepsilon_i$$

where y_i is the outcome, which we wish to correct for the effects of regressor x_i. We are interested in the residual ε_i of this model. Rearranging the terms, we have the following expression for the residual of interest (up to a scaling constant):

$$\frac{y_i}{{x_i}^{\beta_j}} = \alpha \varepsilon_i$$

In our genome-wide association analyses, the phenotypes are constructed according to the left-hand side of this equation, replacing β by its regression estimate, resulting from a log-log regression. That is, we estimate β by re-writing the multiplicative regression as a log-log regression, so it can be estimated using ordinary least squares (OLS):

$$\log(y_i) = \log(\alpha) + \beta_j \log(x_i) + \log(\varepsilon_i)$$

The coefficient β_j represents the percentage change in y_i that follows a one percent change in x_i. If this is smaller or larger than one, the relationship between x_i and y_i is non-linear.

The log-log regression model is often used in economics, where the coefficient is known as an 'elasticity'. An elasticity is a unitless measure of the extent to which one variable changes in response to a change in another variable.

The coefficient from the log-log regression is also sometimes referred to as the "attenuation" or "shrinkage" factor[46]. In health science, the log-log model underlies the construction of BMI – a measure of body weight corrected for body height (two variables that do not scale linearly). In the construction of BMI, body weight is corrected for squared body height (i.e., weight in kilograms, divided by height in meters squared). We confirm that a power of two constitutes the coefficient of a log-log regression of body weight (measured in kilograms) on body height (measured in meters) in the UKB (N = 444,648), with $\hat{\beta}$ = 1.9995 (95% confidence interval = [1.991, 2.008]). Hence, dividing weight by height-squared represents the proper empirical correction factor for height in the UKB, providing an estimate of body density that proxies for body fat composition.

All participating cohorts (with the exception of DietGen) estimate the coefficients from these log-log regressions and use the estimated coefficients to correct the total energy-intake term in the denominator. We report the coefficients obtained from those regressions for seven cohorts included in the meta-analysis (FHS, HRS, RSI/II/III, UKB, and WHI) in **Supplementary Table 2.3**, and we plot the scatterplots and lines of fit for European individuals included in the first

release of the UKB in **Extended Data Figure 1**. Across the seven cohorts, we also report the point estimates, which were obtained with fixed-effects inverse-variance-weighted meta-analysis, where the cohort weights were equal to $1/SE^2$.

In six out of seven cohorts, the coefficient for fat intake is larger than one (all comparisons in this paragraph are statistically distinguishable from one at the 5% level). In one cohort (RSII), the coefficient was smaller than one. The meta-analysis point estimate was $\hat{\beta}$ = 1.158 (95% CI [1.155, 1.161]). In all cohorts, the coefficient for protein intake is smaller than one, with a meta-analysis point estimate $\hat{\beta}$ = 0.817 (95% CI [0.814, 0.820]). The estimates for carbohydrate and sugar intake varied across cohorts. The meta-analyses imply coefficients smaller than one for both carbohydrate and sugar intake, with $\hat{\beta}$ = 0.955 (95% CI [0.952, 0.958]) and $\hat{\beta}$ = 0.921 (95% CI [0.916, 0.926]), for sugar and carbohydrate intake, respectively.

Thus, we find that the reporting of relative macronutrient intake was not constant across different levels of total energy intake. The reported relative consumption of protein, sugar, and carbohydrates tend to decrease at higher levels of reported total energy intake. For fat intake, the reported relative consumption tends to increase at higher levels of reported total energy intake. Thus, according to the self-reports, compared to low energy diets, high energy diets consist of a higher ratio of fat to protein.

2.7 Comparison to regular macronutrient densities

In this section, we show that "regular" macronutrient densities (i.e., percentage intake of macronutrient with respect to total energy intake) tend to leave a residual correlation with total energy intake.

Because high-energy diets tend to consist of relatively less protein and relatively more fat according to the self-reports (**Supplementary Information 2.6**), percentage protein intake is expected to have a negative correlation with total energy intake, while percentage fat intake is expected to have a positive correlation with total energy intake. These residual correlations have been described before, and have led to recommendations of regressing relative macronutrient intake on total energy intake, and using the residual from this analysis for further analyses[24]. (Since we observed non-linear correlations between macronutrient intake and total energy intake, we chose to use the back-transformed residual of a log-log regression as described in **Supplementary Information 2.6**)

In **Supplementary Table 2.4**, we compare how our phenotypes ("adjusted macronutrient densities") phenotypically correlate with total energy intake to how "regular macronutrient densities" phenotypically correlate with total energy intake. We study these correlations in four different cohorts: FHS, HRS, WHI, and UKB. We find sizeable correlations between total energy intake and percentage macronutrient intake, especially for protein intake (range: r = [-0.284 (UKB), -0.055 (WHI)]) and fat intake (range: r = [0.065 (HRS), 0.223 (WHI)]), where the correlations are always statistically distinguishable from zero with $P < 0.001$. As expected, the stronger a macronutrient deviated from a linear relationship with total energy (i.e., the deviation

of the betas from 1 as reported in **Supplementary Table 2.4**), the stronger the correlation with total energy intake.

In some cohorts, we also find correlations between our phenotypes and total energy intake that are statistically distinguishable from zero, but these are always smaller than $r < 0.05$. We believe these small correlation estimates are noise induced by the back-transformation of the regression residual, which is on a log scale as described in **Supplementary Information 2.6**. We conclude that our measure of macronutrient density is preferred to the regular measure of macronutrient density.

2.8 Interpretation

Studying energy-adjusted macronutrient intakes instead of total nutrient intakes has been called the "primary focus of nutritional epidemiology"[24]. Researchers generally do not study total unadjusted nutrient intakes because they are mainly a function of body size and physical activity, with taller, larger, and more physically active individuals requiring higher energy consumption to maintain their metabolic equilibrium[17,18,24]. Hence, associations between total nutrient intakes and health outcomes are uninformative[47], especially if these health outcomes are related to body size and physical activity. In addition, total energy intakes are notoriously under-reported[15]. For these reasons, a proper correction for total energy intake is desired[24,47].

The inherent drawback of correcting the macronutrients for energy intake is that it can and likely does induce correlations between them: if an individual consumes more protein but holds total energy intake constant, he or she *must* consume relatively less energy from the other macronutrients. Hence, relative intake of one macronutrient indirectly represents a substitution effect of the other macronutrients[24].

However, we must point out that in our case induced correlations between macronutrients do not necessarily have to occur. To see this, consider first the simple case of two quantities P_1 and P_2, which add up to unity such that $P_1 + P_2 = 1$. Since we can write $P_2 = 1 - P_1$, then the correlation between P_1 and P_2 is $C(P_1, P_2) = C(P_1, 1 - P_1) = -C(P_1, P_1) = -1$ and thus P_1 and P_2 are necessarily negatively correlated. However, if we have more than two phenotypes and $P_1 + P_2 + \cdots + P_n = 1$, then the correlation between P_1 and P_n is $C(P_1, P_n) = C(P_1, 1 - P_1 - P_2 - \cdots - P_{n-1}$, and this holds for all combinations by permuting the indices. We thus see that these conditions can be satisfied for non-negative correlations between some pairs of phenotypes. The only necessity is that each macronutrient must be negatively correlated with at least one other macronutrient.

In our case a missing piece of the total energy equation is alcohol, which is the final contributor to energy intake in European populations. Hence, the shares of protein, carbohydrate, and fat do not add up to unity. It is then theoretically possible that correlations between the macronutrients fat, protein and carbohydrate are all positive or zero but negatively correlated with relative alcohol intake. Finally, we note that SUGAR is a subset of CARBOHYDRATE and that SATURATED FAT is a subset of FAT, which imposes a positive correlation within these pairs.

We do identify genetic loci that are *not* shared (i.e., do not reach at least a level of suggestive genome-wide significance, with $P > 1\times10^{-5}$) by other macronutrients (**Supplementary Table 5.4**). We could speculate that these loci regulate macronutrient-specific biological processes. However, loci that share association signal across multiple macronutrients may also still have direct biological effects on intake of only one of the macronutrients. For instance, loci with especially large effect sizes or small standard errors (e.g., because of more common allele frequency) might be associated with the other macronutrients, simply because the macronutrients are substituted for each other. This tends to be true in our case; associated loci with the lowest *P* values and highest explained phenotypic variances tend to share signal across macronutrients, while loci that are uniquely associated with one macronutrient tend to have larger *P* values and smaller explained phenotypic variances.

We can illustrate this scenario with the following example. Assume that a locus specifically affects intake of sugar through modulation of sweet-taste preference. For simplicity, assume that sweet-taste preference is solely determined by this locus (i.e., is monogenic). If a person with the "sweet tooth" genotype then consumes more sugar than an individual without said genotype and holds intake of the other macronutrients constant (i.e., consumes the same total energy as the person without said genotype), we would measure a higher relative intake of sugar and a lower relative intake of the other macronutrients in this person's dietary report. If sweet-toothed individuals spread their substitution across macronutrients, meaning that they do not systematically swap sugar for one other specific macronutrient compared to non-sweet-toothed individuals (i.e., swap sugar for a combination of other carbohydrates, fat, protein, and alcohol), the genotype will be associated mainly with sugar and to a lesser extent with the other macronutrients . However, if all sweet-toothed individuals would systematically swap sugar with fat, the locus would be equally associated with intake of both sugar and fat, and we could equally well describe the locus as a fatty-taste-preference locus.

We could imagine a population of three sweet-toothed individuals (person A, B, and C) placing an order in a restaurant, where all order a sugary dessert. A fourth individual (person D) without the sweet-tooth genotype does not order a dessert, but consumes the same total number of calories as the three sweet-toothed individuals. To arrive at the same number of calories as person D, sweet-toothed person A might skip the hamburger (consume relatively less fat and protein); person B might skip the beer (consume relatively less alcohol), while person C might skip the fries (consume relatively less fat and non-sugar carbohydrates). This would describe a scenario where the increase in sugar is compensated with a pattern of substitutions across other macronutrients.

If the sweet-tooth genotype has a large effect on sugar intake, the reduction in the other macronutrients will subsequently also be larger. The association test of the sweet-tooth genotype might then be well-powered to become associated with the non-sugar macronutrients, with the direction of effect being in the opposite direction to the effect for sugar.

From correlational GWAS results, it might then be impossible to tell which macronutrient is directly regulated by the associated locus. This question would require experimental follow-up studies. For instance, *FGF21* has been found to specifically regulate sugar and alcohol intake,

and to not respond to intake of bitter and fatty substances[48]. High *FGF21* levels inhibit the desire to consume sugar[48,49]. However, *FGF21* is also upregulated in response to protein restriction, triggering an adaptive metabolic response to starvation[50]. Hence, *FGF21* might have a true pleiotropic role in both taste-preference regulation and adaptive metabolic response to protein-restriction. In GWAS, the *FGF21* locus is associated with all macronutrients. These findings illustrate the complex mechanisms that might underlie macronutrient intake, and underline the need for experimental follow-up research to elucidate GWAS associations.

2.9 Correction for dieting

In this study, we are interested in identification of genetic variants associated with habitual diet composition, and how these variants in turn relate to health and behavior. Importantly, we are not interested in genetic variants associated with diet composition in individuals on calorie- or macronutrient-restricted diets (RDs). For these individuals, reversed causal effects (i.e., health affecting diet and diet composition) are likely, and RDs are especially popular amongst overweight individuals. We therefore exclude individuals on any RDs when this information was available, and asked cohorts to do the same when this information was available, as detailed in our pre-registered analysis plan. The exclusion-criteria column in **Supplementary Table 1.3** lists whether cohorts were able to exclude individuals on RDs.

3 GWAS, quality control and meta-analysis methods

3.1 Genotyping and imputation

Details of the cohort-specific sample sizes, genotyping and (pre-)imputation parameters, and filters can be found in **Supplementary Table 1.3**.

In the discovery and replication cohorts analyzed in-house (FHS, HRS, RS, UKB, WHI), we preclude SNPs with low INFO-scores and low MAF from the GWAS in order to decrease computational effort. Overall, the preclusion filters are in rough accordance with the sample size dependent, post-GWAS QC-filters implemented by Karlsson Linnér et al[51]. For instance, in the UKB we do not perform GWAS on SNPs with MAF < 0.001 or INFO < 0.6 – which is in accordance with the preselected MAF ≥ 0.001 SNP filter for cohorts of $N \geq 10{,}000$, and slightly more lenient than the INFO ≥ 0.7 SNP filter ultimately applied after the GWAS. We also residualize the GWAS phenotype on the covariates in a phenotypic regression, and use this residualized phenotype in the GWAS, instead of adding covariates to the GWAS regression (**Supplementary Information 3.2.1**). This further reduced GWAS runtime.

At the participant-level, pre-GWAS exclusions vary by cohort, and were informed by: a low individual genotyping rate; sex mismatch; cryptic relatedness (with the exception of UKB and FHS, where related individuals were retained in the analysis and analyzed with mixed linear models as described in **Supplementary Information 3.2.2**); non-European ancestry; and (in some cohorts) aberrant or unexplained heterozygosity or chromosomal abnormalities or withdrawn informed consent (see **Supplementary Table 1.3** for details).

3.2 Association analyses

All analyses were performed at the cohort level according to a pre-specified and publicly archived analysis plan (available at https://osf.io/mt9kt/). The analysis plan specified that genetic discovery would be conducted in the UKB, and replication would be carried out in a meta-analysis of all other cohorts.

Within-cohort association analyses were performed on the 22 autosomes and exclusively in individuals of European ancestry. For the majority of cohorts, OLS regression was applied to unrelated individuals. Mixed linear models were applied to the discovery cohort and one of the replication cohorts (UKB and FHS, respectively) for related individuals, as described in **Supplementary Information 0**.

The OLS association model was

$$Y_i = \beta_0 + \beta_1 SNP_i + \boldsymbol{PC_i}\boldsymbol{\gamma} + \boldsymbol{X_i}\boldsymbol{\alpha} + \boldsymbol{C_i}\boldsymbol{\theta} + \epsilon_i,$$

where Y_i is the unstandardized outcome, SNP_i is the number of effect alleles [0, 1, 2] of the SNP; $\boldsymbol{PC_i}$ is a vector of principal components of the variance-covariance matrix of the genotypic data (estimated on the basis of a QC'd selection of SNPs and individuals, and after the removal of long-range LD regions[52]); and $\boldsymbol{X_i}$ is a vector of demographic control variables. These are sex, age, age^2, sex × age, and sex × age^2 and the number of dietary-intake observations (when applicable). $\boldsymbol{C_i}$ is a vector of cohort-specific technical control variables. The exact set of covariates for each cohort can be found in **Supplementary Table 3.1**. In practice, we first residualized Y_i on all technical and demographic covariates, and then use this residualized phenotype in the GWAS; this was done for all cohorts analyzed by the Social Science Genetic Association Consortium (SSGAC; meaning UKB, and the replication cohorts FHS, HRS, RS, and WHI). In **Supplementary Information 3.2.1**, we show that this procedure does not inflate the false positive rate.

The final maximum SNP-based sample sizes for GWAS discovery in the UKB are N = 264,181 for FAT, PROTEIN and CARBOHYDRATE, and N = 230,648 for SUGAR. Replication is performed in a meta-analysis of 14 cohorts and summary statistics from the DietGen consortium. Prediction is performed with the coefficients from the full meta-analysis, but excluding the holdout cohort (RSI and HRS respectively). All other follow-up analyses are performed with the coefficients from the full meta-analysis (i.e., UKB, 14 replications cohorts, and DietGen).

3.2.1 GWAS on a residualized phenotype, without further controls in the GWAS stage, is conservative

As noted earlier, the GWAS in the cohorts UKB, FHS, HRS, RS, and WHI are performed on a phenotype that was residualized on the covariates. Here, we describe the statistical repercussions of this approach.

Ideally, if one wants to eliminate the effects of a set of covariates on the outcome of interest prior to a GWAS, one should residualize both the regressors and outcome with respect to those covariates, as demanded by the Frisch–Waugh–Lovell (FWL) theorem. By only residualizing the

phenotype with respect to the covariates without residualizing the SNP data with respect to those same covariates, we effectively introduce a form of attenuation bias: we leave some noise in the genotypes—noise in the sense that there may be variation in the genotypes that is correlated with the covariates and that cannot be associated with the phenotype anymore after having residualized the phenotype with respect to those covariates.

This attenuation bias of sorts shrinks our estimated SNP effects slightly towards zero—we say slightly, as we only expect considerable shrinkage for genotypes that correlate strongly with the covariates, but we expect only very few SNPs to have such a strong correlation. In addition, this attenuation bias, together with the fact that there effectively is noise left in the genotypes (as explained above), means that the GWAS has a slightly poorer fit than the proper approach stipulated by the FWL theorem. Hence, the residual variance may be slightly overestimated (except for the case of small *N* and a large number of covariates, where an incorrect number for the true degrees of freedom may bias the estimate of residual variance downwards—but this case is irrelevant in a typical GWAS setting, with large *N*).

Combining the relevant pieces, it is now easy to show that, not only are the estimated SNP effects shrunken towards zero, but so are the *t*-test statistics. Consequently, our residualized approach is a computationally efficient approach that is conservative, yet sacrifices only a minuscule amount of statistical power, as we show below.

Importantly, we do require all relevant variables to be mean-centered. That is, the outcome should be corrected for the linear effects of the covariates as well as an intercept (if not included as a covariate directly), and the GWAS itself should still control for an intercept, even if other covariates are not included.

Now, for a GWAS with covariates in matrix $\mathbf{Z}$ with effects $\boldsymbol{\gamma}$, we show that residualization of the phenotype, $\mathbf{y}$, with respect to the covariates, $\mathbf{Z}$, without including those covariates in the GWAS stage, can only bias the GWAS estimate of a SNP's effect, $\hat{\beta}$, and its *t*-test statistic towards smaller magnitude.

For ease of notation, let us consider only one SNP, $\mathbf{x}$ (so no SNP index is required). The model specification is given by

$$\mathbf{y} = \mathbf{x}\beta + \mathbf{Z}\boldsymbol{\gamma} + \boldsymbol{\varepsilon},$$

where $\boldsymbol{\varepsilon}$ is the error term.

By the Frisch–Waugh–Lovell theorem, the OLS estimate of the SNP effect is now given by

$$\hat{\beta} = (\mathbf{x}'\mathbf{M_Z}\mathbf{x})^{-1}\mathbf{x}'\mathbf{M_Z}\mathbf{y}, \text{ where } \mathbf{M_Z} = \mathbf{I} - \mathbf{Z}(\mathbf{Z}'\mathbf{Z})^{-1}\mathbf{Z}'$$

where the latter is a projection matrix, that removes the linear effects of $\mathbf{Z}$ to whatever vector it is applied to (in this case, $\mathbf{x}$ and $\mathbf{y}$).

Instead of including these covariates in the GWAS, we partial out the effects of $\mathbf{Z}$ on $\mathbf{y}$ (i.e., we take $\mathbf{y}^* = \mathbf{M_Z}\mathbf{y}$) and then regress $\mathbf{y}^*$ on $\mathbf{x}$. This implies that our residual-based GWAS estimate of β is given by

$$\widehat{\beta^*} = (\mathbf{x}'\mathbf{x})^{-1}\mathbf{x}'\mathbf{y}^* = (\mathbf{x}'\mathbf{x})^{-1}\mathbf{x}'\mathbf{M_Z}\mathbf{y}.$$

Noting that $\mathbf{M_Z}$ is symmetric and positive semidefinite, it holds that $(\mathbf{x}'\mathbf{M_Z}\mathbf{x}) \geq \mathbf{0}$. Moreover, as $\mathbf{M_Z}$ eliminates some of the variation in $\mathbf{x}$, it also holds that $(\mathbf{x}'\mathbf{x}) \geq (\mathbf{x}'\mathbf{M_Z}\mathbf{x})$. Consequently

$$\begin{gathered}(\mathbf{x}'\mathbf{x}) \geq (\mathbf{x}'\mathbf{M_Z}\mathbf{x}) \geq \mathbf{0} \\ \Leftrightarrow 0 \leq (\mathbf{x}'\mathbf{x})^{-1} \leq (\mathbf{x}'\mathbf{M_Z}\mathbf{x})^{-1} \\ \Leftrightarrow 0 \leq (\mathbf{x}'\mathbf{x})^{-1}|\mathbf{x}'\mathbf{M_Z}\mathbf{y}| \leq (\mathbf{x}'\mathbf{M_Z}\mathbf{x})^{-1}|\mathbf{x}'\mathbf{M_Z}\mathbf{y}| \\ \Rightarrow 0 \leq \left|(\mathbf{x}'\mathbf{x})^{-1}\mathbf{x}'\mathbf{M_Z}\mathbf{y}\right| \leq \left|(\mathbf{x}'\mathbf{M_Z}\mathbf{x})^{-1}\mathbf{x}'\mathbf{M_Z}\mathbf{y}\right| \\ \Leftrightarrow 0 \leq \left|\widehat{\beta^*}\right| \leq \left|\hat{\beta}\right|.\end{gathered}$$

Hence, the OLS estimate from the regression of the residualized phenotype on the original SNP has a magnitude that is smaller than or equal to the magnitude of OLS estimate of the regression of the original phenotype on the SNP, controlling for the covariates in that regression. Moreover, as the sign of the two respective estimators is determined only by $\mathbf{x}'\mathbf{M_Z}\mathbf{y}$, it holds that sign($\widehat{\beta^*}$) = sign($\hat{\beta}$). Hence, residualization merely reduces the magnitude of the estimator without changing the sign.

Regarding *t*-test statistics used for our inferences, it holds that

$$t = (\mathbf{x}'\mathbf{M_Z}\mathbf{x})^{1/2}\hat{\beta}/\hat{\sigma} \text{ and } t^* = (\mathbf{x}'\mathbf{x})^{1/2}\widehat{\beta^*}/\widehat{\sigma^*}$$

Hence,

$$t = (\mathbf{x}'\mathbf{M_Z}\mathbf{x})^{-1/2}\mathbf{x}'\mathbf{M_Z}\mathbf{y}/\hat{\sigma} \text{ and } t^* = (\mathbf{x}'\mathbf{x})^{-1/2}\mathbf{x}'\mathbf{M_Z}\mathbf{y}/\widehat{\sigma^*},$$

where $\hat{\sigma}$ and $\widehat{\sigma^*}$ are the estimators of standard deviation of $\boldsymbol{\varepsilon}$, described next.

If $\mathbf{x}$ and $\mathbf{Z}$ were orthogonal (i.e. $\mathbf{Z}'\mathbf{x} = \mathbf{0}$), the regression residuals, $\mathbf{e}$, resulting from the regression of $\mathbf{y}$ on $\mathbf{x}$ and $\mathbf{Z}$ jointly would be identical to the residuals, $\mathbf{e}^*$, from the regression of $\mathbf{y}^*$ on $\mathbf{x}$. Then, using the usual unbiased estimator,

$$\widehat{\sigma^*} = \frac{1}{\sqrt{N-1}}\sqrt{\mathbf{e}'\mathbf{e}} \quad \text{and} \quad \hat{\sigma} = \frac{1}{\sqrt{N-C-1}}\sqrt{\mathbf{e}'\mathbf{e}},$$

where C is the number of regressors in $\mathbf{Z}$ and N the number of observations. Therefore,

$$\widehat{\sigma^*} = \sqrt{\frac{N-C-1}{N-1}}\hat{\sigma}.$$

Hence, provided $N \gg C$ (which should be the case) and orthogonal genotypes and covariates, $\widehat{\sigma^*} \approx \hat{\sigma}$. In case, however, there is some correlation between $\mathbf{x}$ and $\mathbf{Z}$, we have that $\sqrt{\mathbf{e}'\mathbf{e}} \leq \sqrt{\mathbf{e}^{*\prime}\mathbf{e}^*}$, in which case (again provided $N \gg C$), by good approximation, it follows that $\widehat{\sigma^*} \geq \hat{\sigma}$.

In the same way we showed that $\left|\widehat{\beta^*}\right| \leq \left|\hat{\beta}\right|$, we can show that

$$\left|(\mathbf{x}'\mathbf{x})^{-1/2}\mathbf{x}'\mathbf{M_Z}\mathbf{y}\right| \leq \left|(\mathbf{x}'\mathbf{M_Z}\mathbf{x})^{-1/2}\mathbf{x}'\mathbf{M_Z}\mathbf{y}\right|.$$

Hence, $\left|(\mathbf{x}'\mathbf{x})^{-1/2}\mathbf{x}'\mathbf{M_Z}\mathbf{y}/\widehat{\sigma^*}\right| \leq \left|(\mathbf{x}'\mathbf{M_Z}\mathbf{x})^{-1/2}\mathbf{x}'\mathbf{M_Z}\mathbf{y}/\hat{\sigma}\right|$ (approximately) and therefore $|t^*| \leq |t|$ (approximately). Moreover, as with the estimates of β, we have that sign(t^*) = sign(t).

Consequently, the GWAS t-test statistic for a SNP, using a phenotype that has been residualized with respect to the covariates prior to the GWAS, without inclusion of those same covariates in the GWAS itself, provides a credible, conservative lower bound to the GWAS t-test statistic including the covariates.

We note that even when **x** and **Z** are completely orthogonal—in which case test statistics are inflated under our approach—for typical values of N and C, the inflation is negligible, as can be seen in **Supplementary Table 3.2**. For example, even in a sample of 50,000 observations in a study with 100 covariates, the inflation in test statistics of any given SNP is at most 0.10%. Now, considering the P value of a SNP that is not correlated with any of the covariates and has a P value around the level of genome-wide significance in this sample: if the estimated SNP effect is just genome-wide significant here (i.e., $|t| = 5.453$, with P value = 4.976×10^{-8} under 49,899 degrees of freedom), the maximum possible inflation would only increase $|t|$ by 0.10% and decrease the P value by 1.65×10^{-9} (i.e., we then have $|t^*| = 5.459$ with P value = 4.811×10^{-8} under 49,999 degrees of freedom).

To summarize, our approach has a strong tendency to be conservative in terms of the inferences we make. The more strongly a SNP is correlated with the covariates, the more conservative our inferences will be. Moreover, even for the SNPs for which our approach inflates the magnitude of the test statistics, this inflation is negligible for realistic sample sizes and number of covariates.

3.2.2 Linear mixed model association analysis

As noted earlier, we perform GWAS in FHS and UKB with linear mixed models (LMMs). We do this to leverage information from the substantial numbers of (cryptically) related individuals in these cohorts. An additional benefit is that LMM analysis also performs a better correction for population stratification than can be achieved with the use of a limited number of PCs[53]. However, we still residualize the phenotypes on 10 and 20 PCs, respectively, prior to performing GWAS.

Both cohorts calculated their own set of PCs on the basis of a selection of high quality, independent SNPs, and unrelated individuals. For UKB, the procedure is described in the Supplementary Materials of Bycroft et al.[54]. For both UKB and FHS, we calculate the median "effective sample size" (as described in **Supplementary Information 3.4.1**) across the SNPs that were retained after quality control. This median effective sample size forms the meta-analysis weight in our N-weighted meta-analysis of SNP effects (**Supplementary Information 3.4**).

UKB

In UKB, we performed GWAS with BOLT-LMM software v2.2[53]. In the genetic variance component, we included 723,483 directly genotyped bi-allelic SNPs with MAF > 0.001, and SNP call rate > 0.9. In accordance with the protocol used in Karlsson Linnér et al.[51], we only included individuals of self-reported European ancestry (i.e., British, Irish, or any other European background) whose PC score on the first PC of the genotype matrix was less than 0, as this identified a cluster of individuals of European ancestry. This cutoff was based on visual

inspection of the histogram of the first PC, which clearly demarcates individuals of European ancestry from the other ancestries. Both in this quality control procedure and in the GWAS, we make use of the PCs that were calculated by UKB[54] on the basis of the full UKB sample (which included individuals from all ancestries) with fastpca[55] software. Other participant filters are reported in **Supplementary Table 1.3**.

FHS

In FHS, we performed linear mixed model association with the use of GCTA[56]. Here, we implemented MLMA-LOCO[57], or "Mixed Linear Model Association – Leave One Chromosome Out". This procedure removes the chromosome on which the focal SNP is located from the genetic relatedness matrix. The analysis excluded SNPs with MAF < 0.01, and individuals with with genotyping call rate < 0.95.

3.2.3 Auxiliary GWAS

In addition to the sex-pooled GWAS of the four macronutrients, we also perform sex-stratified GWAS of the four macronutrients in the UKB and a sex-pooled GWAS for relative saturated-fat intake ("SATURATED FAT") in the UKB. For the sex-stratified GWAS, we perform LDSC genetic correlation analyses to assess whether the genetic architecture of diet composition is sex-specific. For SATURATED FAT, we assess the genetic correlations with the other macronutrients as well as genetic correlations with other traits.

3.3 Quality control

3.3.1 Reference panel

The reference panel used throughout this project for SNP-based quality control and LD calculations is based on the v1.1 release from the Haplotype Reference Consortium[58] (HRC, downloaded from the European Genome-phenome Archive on August 1, 2017), with genomic positions aligned to Genome Reference Human genome build 37 (GRCh37). Details on how this reference panel was constructed can be found in Karlsson Linnér et al.[51]. In brief, an internal quality control procedure removed any non-autosomal SNPs, SNPs with duplicate SNP positions, multi-allelic SNPs, or strand inconsistencies with the UK10K[59] haplotype reference panel. Subsequently, one individual of a cryptic pair (genetic relatedness > 0.025) was removed, as implemented with the use of PLINK 1.9[60,61]. Finally, the total sample size in this reference panel is $N = 17{,}774$, containing 38,889,224 bi-allelic autosomal SNPs that passed internal QC.

3.3.2 Cohort-level quality control

Post-GWAS, pre-meta-analysis quality control (QC) at the cohort-specific summary statistics level was performed with EasyQC software[62]. In accordance with QC-protocols from Winkler et al.[62] and the SSGAC[63,64], genetic variants were removed if they met any of the following criteria:

1. Missing values for: the effect or non-effect allele, MAF, *N,* beta, standard error, INFO-score, allele frequency, or the imputed/genotyped indicator;
2. Nonsensical values (e.g., P value < 0 or MAF > 1, imputation-quality score > 1; negative or infinite betas or standard errors).

3. More than one coded allele (indicative of a structural variant) or reported structural variants (e.g., indels);
4. Low minor allele frequency (MAF);
5. Low SNP call rate;
6. Low imputation-quality INFO-score;
7. Low *P* value for the Hardy-Weinberg Equilibrium test (if available);
8. Absence (or mismatched) alleles with respect to our HRC reference panel;
9. Explained variance for the SNP in the phenotype (R^2) of higher than 5%, based on the SNP's MAF and phenotype's standard deviation;
10. Standard error for the SNP that was 1.4 times higher than expected based on the SNP's MAF and phenotype's standard deviation.

A number of these parameters vary by cohort as a function of cohort sample size; the cohort-specific details can be found in **Supplementary Table 1.3**. **Supplementary Table 3.3** lists, for each cohort, the number of SNPs that entered the quality control procedure and the number dropped at each step.

For the DietGen consortium summary statistics, the filters 9 and 10 could not be applied because we do not have access to the phenotypes' pooled standard deviations. In the UKB, we also drop all SNPs not imputed to the HRC panel, as these were not aligned properly to their genomic coordinates in the second release of the UKB of July 2017[c].

After filtering out low-quality SNPs, we visually inspected diagnostic plots generated by EasyQC to flag potentially remaining QC issues. These were generated separately for each cohort.

Of interest are:

1. Q-Q plots, to inspect excessive inflation ("early liftoff") of *P* values due to remnant population stratification;
2. Allele frequency plots, which compare the observed allele frequency in the cohort to the allele frequency in our HRC reference panel. Large or systematic discrepancies could indicate, for instance, strand issues (e.g., 'allele flips') or inadequate filtering of ancestry outliers. Note that we used this plot to detect systematic errors, but we did not actually remove any large MAF outliers with respect to the reference panel.

The other EasyQC plots (*SE*-*N* plots, *P*-*Z* plots) were also used to detect potential internal analytical errors. Finally, we also generated a plot that compared the expected SNP effect standard errors (for a random sample of 10,000 SNPs, based on the reported phenotype's standard deviation) to the reported SNP effect standard errors. This plot was used to assure that the reported phenotypic standard deviation was correct.

---

[c] https://biobank.ctsu.ox.ac.uk/crystal/label.cgi?id=100319

3.3.3 Post meta-analysis QC

After meta-analysis was performed, we remove SNPs that were available in less than 50% of the phenotype-specific total maximum sample size.

3.3.4 Locus definition

Since the human genome is characterized by widespread LD between SNPs, significantly associated SNPs generally tend to cluster with other significantly associated SNPs within the same genomic region. When one of these regions carries a SNP that reaches genome-wide significance, we call this region the associated *locus.* The SNP with the lowest *P* value in that locus is deemed the "lead SNP". (The lead SNP is not necessarily the causal variant in the locus; it may just be the most accurately genotyped or imputed variant in that particular region.)

In order to establish the number of independently associated loci for the traits in this study, we use the "clumping" algorithm in PLINK[61], which ascertains the set of SNPs that are in LD with the lead SNP (as based on calculations in our HRC reference panel). PLINK first takes the SNPs that reach genome-wide significance, and then "clumps" SNPs in their genomic region into its locus. Inclusion of SNPs into a "clump" (i.e., independent genomic region) is based on an LD threshold (in our case, $r^2 = 0.1$, which is the squared correlation of the lead SNP with the SNPs in its vicinity), association with the phenotype at some *P* value threshold (in our case, $P < 0.01$, which is mainly implemented to remove SNPs from the clumps to drastically decrease the algorithm's runtime), and genomic distance. In our case, we set the genomic distance parameter to be 100,000,000 kilobases (kb), effectively making the LD- and *P* value-thresholds the only binding parameters.

3.4 Meta-analysis

3.4.1 Meta-analysis scheme

Fixed effects meta-analysis of SNP effects (i.e., SNP *Z*-scores) is performed with METAL[65] software, using a sample size weighting scheme. Sample size weighting is performed at the level of the SNP's *Z*-statistics across cohorts, where the SNP's overall *Z*-score for SNP *i* across cohorts *J* can be defined as

$$Z_i = \frac{\sum_j^J Z_{i,j} w_j}{\sqrt{\sum_j^J w_j^2}},$$

where the weight for cohort *j* is defined as

$$w_j = \sqrt{N_j}.$$

For the two cohorts that used a mixed linear model (in which related individuals were included), we calculate the effective sample size for SNP *i* by rearranging the formula for the SNP standard error:

$$N_{i,j} = \frac{SD_Y^2}{SE_{i,j}^2 \times 2 \times MAF_{i,j} \times (1 - MAF_{i,j})},$$

where SD_Y^2 is the variance in phenotype Y and $SE_{i,j}$ is the unstandardized standard error for SNP i in cohort *j*. For each cohort's meta-analysis weight, we use the median effective sample size across quality-controlled SNPs *J*.

Throughout this study, we measure the SNP effect size by the semi-standardized beta ($\hat{\beta}_i$, described next) and the explained variance in the phenotype by the incremental adjusted R^2. We calculate the incremental adjusted R^2 by first regressing the phenotype on a set of covariates (described in more detail in **Supplementary Information 9.1**), and then estimating the same regression with the phenotype's polygenic score as an additional covariate. The difference in adjusted R^2 between the two models is the incremental adjusted R^2 which thus denotes the additional explained phenotypic variance from the genotype after accounting for covariates that are included in the GWAS (or residualized from the phenotype). The semi-standardized beta is standardized with respect to the phenotype but *not* with respect to the genotype; it is the effect size in standard deviations of the phenotype per effect allele. The semi-standardized beta is calculated using the approximation (as derived in the Supplementary Note from Rietveld et al.[66], page 5)

$$\hat{\beta}_i = Z_i \times SE_i,$$

where the Z-score for SNP i is estimated in the meta-analysis, and the semi-standardized standard error for SNP i is calculated as

$$SE_i = \left(\sqrt{2 \times N_i \times MAF_i \times (1 - MAF)}\right)^{-1}.$$

The incremental R^2 for SNP i is then calculated using the approximation

$$R_i^2 = 2 \times MAF_i \times (1 - MAF_i) \times \widehat{\beta_i}^2.$$

To correct the inflation of SNP estimates for population stratification, we supply METAL[65] with the intercepts obtained from LD Score regression[67]. METAL then deflates the SNP's *Z*-statistics with the square root of the LDSC intercept, giving rise to a genomic-controlled *Z*-statistic for SNP *i:*

$$Z_{i_{GC}} = \frac{Z_i}{\sqrt{LDSC_{intercept}}}$$

4 Replication

4.1 Genetic correlations between discovery and replication cohorts

We use bivariate LD Score regression (method described in **Supplementary Information 10**) to examine the comparability between the summary statistics from our discovery cohort, the 14 replication cohorts, and DietGen (where the latter two make up our combined replication phase). The results are reported in **Supplementary Table 4.1**.

4.1.1 Discovery vs. 14 replication cohorts

We find the genetic correlation between our discovery and 14 replication cohorts is statistically significantly greater than 0 for all phenotypes. Further, we fail to reject the hypothesis that the genetic correlation is unity for FAT ($\hat{r}_g$= 0.997), CARBOHYDRATE ($\hat{r}_g$= 1.14[d]) and SUGAR ($\hat{r}_g$= 0.997). We find a genetic correlation of less than 1 (but greater than 0) for PROTEIN ($\hat{r}_g$= 0.686), which implies that the predictive power of the protein polygenic score will be lower than expected based on theoretical calculations that assume a perfect genetic correlation.

4.1.2 Discovery and 14 replication cohorts vs. DietGen

We also examine the genetic correlations between DietGen and (1) the 14 replication cohorts and (2) the discovery cohort and 14 replication cohorts meta-analyzed together. We find strong positive genetic correlations for all phenotypes: FAT ($\hat{r}_g$= 0.78, *SE* = 0.18), PROTEIN ($\hat{r}_g$= 0.88, *SE* = 0.19) and CARBOHYDRATE ($\hat{r}_g$= 0.71, *SE* = 0.12). Further, we fail to reject the null hypothesis (at the 1% significant level) that this genetic correlation is different from 1 for all phenotypes, although CARBOHYDRATE is significantly different from 1 at the 5% level. When we compare the DietGen results to only our 14 replication cohorts meta-analyzed together (i.e., without the discovery phase) we find strong genetic correlations that are statistically indistinguishable from unity (*P* value > 0.25), with $\hat{r}_g$= 1.21 (*SE* = 0.31) for FAT, $\hat{r}_g$= 0.75 (*SE* = 0.22) for PROTEIN, and $\hat{r}_g$= 0.82 (*SE* = 0.20) for CARBOHYDRATE. We conclude that our results are broadly consistent with the most similar previous study.

4.1.3 Discovery vs. full replication phase

We also report the genetic correlations between the discovery and full replication phase (where the "full replication phase" consists of the 14 cohorts and DietGen). Here, we find an especially high correlation for CARBOHYDRATE ($\hat{r}_g$= 0.98, *SE* = 0.10) which is statistically indistinguishable from unity and smaller correlations for FAT ($\hat{r}_g$= 0.79, *SE* = 0.09) and PROTEIN ($\hat{r}_g$= 0.77, *SE* = 0.10), which are significantly different from 1 at the 5% level.

4.1.4 EPIC-InterAct vs. full meta-analysis

Finally, we report the genetic correlation between the two EPIC-InterAct cohorts meta-analyzed (combined *N* = 12,722) and all other remaining cohorts meta-analyzed (combined *N* = 222,669) for SUGAR, since the two EPIC-InterAct cohorts analyzed added sugars instead of total sugars (**Supplementary Information 2.3**). We find that the genetic correlation does not differ significantly from 0 nor 1 ($\hat{r}_g$= 0.71, *SE* = 0.47), indicating that this genetic correlation analysis is underpowered at the current EPIC-InterAct sample size. However, we note that we find one additional GWAS hit for SUGAR after including the two EPIC-InterAct cohorts, reflecting the small increase in power for genetic discovery with the addition of EPIC-InterAct.

---

[d] Bivariate LDSC regression does not restrict the estimated genetic correlation to the interval [-1,1].

4.1.5 Discussion

The results show that the 14 replications cohorts correlate very highly with the discovery cohort for FAT, CARBOHYDRATE and SUGAR. For PROTEIN, the correlation is significantly different from 1, suggesting decreased power to replicate individual SNPs in these cohorts. When we add DietGen to the 14 cohorts (giving rise to our full replication phase), we see that the genetic correlation increases for PROTEIN, slightly decreases for CARBOHYDRATE, and decreases moderately for FAT. This indicates that with the addition of the DietGen cohort, we especially gain power for genetic discovery and replication for PROTEIN and CARBOHYDRATE, but less so for FAT.

Aside from differences in population characteristics, differences between the discovery and 14 replication cohorts may be driven by the fact that the discovery cohort used a different diet questionnaire method than the replication cohorts (24HDR vs. FFQ, see **Supplementary Information 2.5.1**), while differences between the discovery cohort (and the 14 replication cohorts) and DietGen may be driven by differing phenotype definitions (see **Supplementary Information 2.6).** Even given these imperfect genetic correlations at the 5% level, we stress that meta-analyzing with highly genetically (yet imperfect) correlated data still gives us an advantage in terms of statistical power for genetic discovery. This power tradeoff between quality (in terms of harmonized phenotypes) and quantity (in terms of sample size) has been described in detail in previous work[63,68].

4.2 Replication of GWAS hits from the discovery stage

In this section, we discuss the GWAS findings from the discovery stage, and we assess the credibility of the results from our discovery GWAS by replicating the associations of its lead SNPs in our replication GWAS of the same phenotypes. As planned at the inception of this study (and pre-registered in our analysis plan on Open Science Framework, https://osf.io/mt9kt/), we use the UK Biobank as our discovery cohort and all the other cohorts as replication samples that we then meta-analyze together. We begin by describing the methods for replication analyses, followed by the replication results and how they compare to expected replication rates. In the final subsection, we demonstrate that we do not lose much statistical power by splitting the total sample into the UKB as discovery and the rest as replication, compared to the optimal split calculated from simulations.

4.2.1 Methods

General methods

Our replication analyses closely follow the procedure outlined in Supplementary Information section 1.8 of Okbay et al.[64]. We use binomial tests to assess whether the independent lead SNPs from our discovery GWAS replicate in an independent replication GWAS. Under the null hypothesis that each of the lead SNPs is null in both the discovery and replication sample, we would expect 50% of the SNPs to have concordant signs and 5% to be statistically significantly different at the 5% level. Hence, it follows that the number of SNPs that have concordant signs or that are above a certain significance threshold is distributed as Binomial (M, π), where M is

the total number of independent lead SNPs and π is the expected fraction of sign-concordant or significant SNPs. We conduct one-sided binomial tests for both the sign concordance of the lead SNPs and the number of lead SNPs from our discovery GWAS that are statistically significantly different at the 5% level (both with and without Bonferroni correction) in the replication GWAS. The Bonferroni-corrected *P* value threshold is determined separately for each phenotype as 0.05 divided by the number of lead SNPs from the discovery GWAS for that phenotype.

We use the UKB data as our discovery sample. While most of the independent lead SNPs identified in the discovery GWAS are directly available in the replication GWAS, we find that one of the lead SNPs for CARBOHYDRATE was missing in the replication sample. For this missing lead SNP, we replace it with a suitable proxy that satisfied the following conditions: i) the SNP is available in both the discovery GWAS and the replication GWAS, ii) the SNP is within 500 kb of the original lead SNP in the discovery sample, and iii) the SNP has the lowest *P* value in the discovery GWAS among those that satisfy i) and ii). Specifically, we replace lead SNP rs7502280 with rs2435204 ($r^2 = 0.487$).

Calculation of expected replication records

In addition to conducting binomial tests, we calculate the *expected* rate of replication (given the discovery GWAS results, the discovery sample size and the replication sample size) using simulations, and we assess whether these expected rates matched the observed replication rates. The results from these analyses might be more informative than the binomial tests, since the null hypothesis in the binomial tests posits zero association in the replication sample (which is unlikely, given the high genetic correlation between the discovery and replication results, as shown in **Supplementary Table 4.2**). Moreover, since the number of lead SNPs in the discovery sample is relatively small, the binomial tests do not have much power to reject the null hypothesis.

Following the procedure outlined in Okbay et al.[64], we first conduct a Bayesian Winner's Curse correction to obtain estimates of the posterior distribution of the SNPs' true effect sizes, given their GWAS estimates. Given this estimate of the posterior distribution, we then conduct simulations to compute the expected sign concordance and replication record. In each of our 10,000 simulations, we draw a true effect size (β_j) for each SNP j from the estimated posterior distribution and added Gaussian noise to generate discovery and replication GWAS estimates for each SNP as follows:

$$\hat{\beta}_{Discovery,j} = \beta_j + \epsilon_j \hat{\sigma}_{Discovery,j}$$

$$\hat{\beta}_{Replication,j} = \beta_j + \delta_j \hat{\sigma}_{Replication,j},$$

where ϵ_j and δ_j are independently drawn from a standard normal distribution, and the standard deviations of the estimation error can be approximated as $\hat{\sigma}_{Discovery,j} \approx 1/\sqrt{N_{Discovery}}$ and $\hat{\sigma}_{Replication,j} \approx 1/\sqrt{N_{Replication}}$. Then, we record the number of SNPs that are concordant in sign and also the number of SNPs that replicate with the same sign at the 5% level (both with and without Bonferroni correction). Finally, we obtain the expected sign concordance and the expected replication record by averaging these numbers across the 10,000 simulations.

Results

Supplementary Table 4.2 summarizes the results, and **Extended Data Figure 3** compares the effect sizes (and 95% confidence intervals) of the lead SNPs that reached genome-wide significance in the discovery phase to their effect sizes observed in the replication phase.

In this discovery GWAS of our four phenotypes – FAT, PROTEIN, SUGAR, and CARBOHYDRATE – we found 4, 5, 5 and 7 independent lead SNPs, respectively. The independent lead SNPs were obtained by clumping the GWAS summary statistics using the Haplotype Reference Consortium (HRC) reference panel with the thresholds defined in **Supplementary Information 3.3.4.** After taking overlapping loci between phenotypes into account, we identify 12 unique loci discovered in the UKB.

Overall, three of our four macronutrients – PROTEIN, SUGAR, and CARBOHYDRATE – reject the null hypothesis of (random) 50% sign concordance, while all four macronutrients reject the null hypothesis of no association even after phenotype-specific Bonferroni correction for the number of associated lead SNPs in the discovery stage. In addition, the observed number of SNPs that match in signs and the observed number of SNPs that are statistically significant are close to their theoretical predictions using the simulation procedure described above.

FAT. Among the four independent lead SNPs identified from the discovery GWAS, all have matching signs, three of them are significantly different from zero at the 5% level, and two of them are significant at the Bonferroni-corrected 5% level in the replication sample. Using a Binomial test with distribution (4, π) where π is the expected fraction of sign-concordant or significant SNPs, we can construct *P* values associated with these results as described in section 4.1.1. The *P* value associated with the sign concordance is then 0.063, the *P* value associated with the number of SNPs significant at the 5% level is <0.001, and the *P* value associated with the number of SNPs significant at the Bonferroni-corrected 5% level is <0.001. Our calculation for expected replication indicates that 3.7 SNPs (95% CI 3.0 to 4.0) are expected to have concordant signs, 2.0 SNPs (95% CI 0 to 4.0) are expected to be significant at the 5% level and 1.3 SNPs (95% CI 0 to 3.0) are expected to be significant at the Bonferroni-corrected 5% level. Comparing these to the observed numbers—4, 3, and 2, respectively—shows that the replication record of our lead SNPs is close to what we would expect, given our discovery GWAS estimates and discovery and replication sample sizes.

PROTEIN. All of the five lead SNPs from the discovery GWAS have concordant signs, and four of them are significant at both the 5% level and the Bonferroni-corrected 5% level in the replication sample. The *P* value associated with the sign concordance is 0.031, the *P* value associated with the number of SNPs significant at the 5% level is <0.001, and the *P* value associated with the number of SNPs significant at the Bonferroni-corrected 5% level is <0.001. Our calculation for expected replication indicates that 4.4 SNPs (95% CI 3.0 to 5.0) are expected to have matching signs, 1.9 SNPs (95% CI 0 to 4.0) are expected to be significant at the 5% level and 1.0 SNP (95% CI 0 to 3.0) is expected to be significant at the Bonferroni-corrected 5% level. The observed numbers (i.e. 5, 4, and 4, respectively) surpass these expectations and show that the replication record was stronger than predicted.

SUGAR. All of the five lead SNPs from the discovery GWAS have concordant signs and are significant at the 5% level and the Bonferroni-corrected 5% level in the replication sample. The *P* value associated with the sign concordance is 0.031, the *P* value associated with the number of SNPs significant at the 5% level is <0.001, and the *P* value associated with the number of SNPs significant at the Bonferroni-corrected 5% level is <0.001. Our calculation for expected replication indicates that we are expected to see 4.3 SNPs (95% CI 3.0 to 5.0) that match in signs, 1.3 SNPs (95% CI 0 to 3.0) that are significant at the 5% level and 0.5 SNPs (95% CI 0 to 2.0) that are significant at the Bonferroni-corrected 5% level. The observed numbers (i.e. 5, 5, and 5, respectively) are all above these expected numbers.

CARBOHYDRATE. Among the seven lead (or proxy-lead) SNPs, we found that all seven of them have concordant signs, three of them are significant at both the 5% level and the Bonferroni-corrected 5% level in the replication sample. The *P* value associated with the sign concordance is 0.008, the *P* value associated with the number of SNPs significant at the Bonferroni-corrected 5% level is 0.004, and the *P* value associated with the number of SNPs significant at the Bonferroni-corrected 5% level is <0.001. Our calculation for expected replication indicates that 6.8 SNPs (95% CI 6.0 to 7.0) are expected to have concordant signs, 4.4 SNPs (95% CI 2.0 to 7.0) are expected to be significant at the 5% level and 2.6 SNPs (95% CI 0 to 5.0) are expected to be significant at the Bonferroni-corrected 5% level. The observed numbers (i.e. 7, 3 and 3, respectively) show that the replication record of our lead SNPs is close to expectation.

Ex post analysis of statistical power for replication

At the inception of this study, we decided to use the UKB as the discovery sample and all additional recruited cohorts as the replication sample. Despite some potential loss in statistical power, we believed that doing so would make our study more transparent and not dependent on how we split the total sample for discovery and replication. In this subsection, we calculate the optimal split and gauge our loss in statistical power through simple simulations that are similar in nature to the simulations used for calculating the expected replication record described above.

We first obtain estimates of the distribution of the SNPs' true effect sizes, conditioning on the fraction of total sample size used as replication sample and using some key summary statistics from GWAS results of all cohorts meta-analyzed. We assume a "spike-and-slab" mixture distribution of effect sizes, where a fraction of SNPs $(1-\pi)$ is causal, and a fraction of SNPs (π) is not:

$$\beta_{j,std} \sim \begin{cases} 0 & \text{with probability } \pi \\ N(0,\tau^2) & \text{with probability } 1-\pi. \end{cases}$$

After estimating π from the GWAS summary statistics of total sample, we used the mean χ^2 statistics of all SNPs from the total GWAS to infer the mean χ^2 statistics of only the associated SNPs from the following relationship:

$$1 * \pi + \overline{\chi^2}_{associated} * (1-\pi) = \overline{\chi^2}_{all\ SNPs},$$

where we use the fact that the mean χ^2 statistics of the null SNPs is 1. Then, conditioning on the fraction of total sample size used for replication, we obtain an estimate of mean χ^2 statistics that

we would have gotten from the discovery GWAS, $\overline{\chi^2}_{Discovery}$, by scaling $\overline{\chi^2}_{associated} - 1$ by the fraction of total sample size used for discovery. Lastly, we approximate the variance of the causal SNPs (τ^2) by $\frac{\overline{\chi^2}_{Discovery}-1}{N_{Discovery}}$. The rest of our simulations proceed in the same manner as those used for calculating the expected replication record. We vary the fractions of total sample size used for replication from 5% to 40% in 5 percentage-point increments and repeat the simulation 10,000 times for each replication fraction. The optimal fraction of replication sample is determined as the fraction that maximizes the expected number of SNPs that are significant at the 5% level with Bonferroni correction in the replication sample. The Bonferroni-corrected *P* value threshold is determined as 0.05 divided by the number of genome-wide significant SNPs in simulated discovery GWAS at each iteration of the simulations.

Our simulation results indicate that the optimal replication sample fractions are 30%, 25%, 25% and 20% for FAT, PROTEIN, SUGAR and CARBOHYDRATE, respectively, whereas the actual split as we had planned (i.e., UKB for discovery and all other cohorts for replication) corresponds to 33%, 33%, 33% and 26%, respectively. When we compare the expected numbers of SNPs that are significant at the Bonferroni-corrected 5% level under the optimal and actual splits, they are 0.37 and 0.32 (for FAT), 0.37 and 0.33 (for PROTEIN), 0.18 and 0.18 (for SUGAR) and 1.50 and 1.46 (for CARBOHYDRATE). Though the expected numbers are slightly lower under the actual split, the differences are very small and all within the magnitude of 0.01. The simulation results provide evidence that our loss in statistical power due to our choice of how to split cohorts into discovery and replication samples is minimal.

4.3 Replication of *DRAM1* variant rs77694286

A previous GWAS of macronutrient intake performed by Merino et al.[69] identified a rare variant in *DRAM1* (rs77692486, $MAF_{1000GEUR} = 0.01$). However, this SNP only reached genome-wide significance (for PROTEIN) in their discovery phase, with the effect allele having a discordant sign (with *P* = 0.16) in their UKB replication phase. Here, we report the replication record for this SNP in our own meta-analyses. The sample overlap between our GWAS and Merino et al.'s GWAS is described in **Supplementary Table 4.3**. It is impossible to estimate the exact extent of overlap, because even for the same cohorts the sample sizes differed – likely because of expanded genotyping efforts and/or differences in QC protocols. The overlapping cohorts for the rs77694286 analysis are Fenland; RS; and UKB. Merino et al. also used EPIC-Norfolk data. Since EPIC-Norfolk participants overlap with EPIC-InterAct, there is also possible partial overlap there (EPIC-InterAct is one of our included cohorts). The estimated maximum sample overlap between Merino et al.'s non-UKB cohorts and our non-UKB cohorts for rs77694286 is *N* = 28,927 (EPIC-InterAct, Fenland, and RS) / *N* = 123,659 (Merino et al.'s non-UKB cohorts) = 22.9%. Despite this overlap, rs77694286 does not come even close to genome-wide significance, as detailed below. In our own meta-analysis, we removed rs77692486 from most individual cohorts during individual cohort quality control (QC), mostly because it has a too low MAF according to our sample-size-specific MAF criteria. Prior to QC, the SNP is available in 12 cohorts, with valid and non-missing summary statistics in all 12. Here, we meta-analyze the

SNPs across these cohorts for PROTEIN, and additionally across all cohorts minus UKB, since the effect was non-significant in UKB in the Merino et al. analysis. The results are displayed in **Supplementary Table 4.3**. We also calculate the corresponding (semi-standardized) effect sizes from Merino et al., and display them in the same table for comparison. In our meta-analysis that includes UKB ($N = 212{,}527$), the SNP does not nearly reach genome-wide statistical significance and the effect size was discordant (semi-standardized $\hat{\beta} = -0.028$ for allele G, $P = 0.271$) with respect to the effect size discovered by Merino et al. (semi-standardized $\hat{\beta} = 0.121$ for allele G, $P = 1.9 \times 10^{-9}$). In the meta-analysis that excludes UKB, the effect is concordant with respect to Merino et al. (semi-standardized $\hat{\beta} = 0.028$ for allele G, $P = 0.560$; Merino et al. semi-standardized $\hat{\beta} = 0.054$ for allele G, $P = 7.5 \times 10^{-5}$) but indistinguishable from zero.

We conclude that the rare variant rs77692486 does not currently appear to be a robust association for relative protein intake. Other independent large samples are needed to further study this association. Our results strengthen the notion that the interpretation of rare variant associations for complex traits with relatively small GWAS sample sizes requires extra caution.

5 GWAS results from the combined meta-analyses

5.1 LD Score regression intercept test for population stratification bias

An important concern in GWAS studies is the potential confounding bias in SNP effects resulting from uncorrected-for population stratification. Population stratification refers to differences in genetic ancestry among participants included in the study. When differences in genetic ancestry (and thus, SNP allele frequencies) correlate with phenotypic differences, GWAS estimates can be biased. A salient example is a hypothetical study on chopstick use in a population that includes both East Asian and non-East Asian participants[70]. This study will find spurious associations for SNPs that differ in allele frequency between the East Asian and non-East Asian participants, which could then be wrongly identified as "chopsticks genes".

Although we only include individuals of European ancestry in the current study, subtle differences between the different included European populations might still bias SNP effects, resulting in an overall inflation of the genome-wide SNP test statistics. To counteract this problem, all included cohorts added at least 5 principal components of the genetic relatedness matrix as covariates in their GWAS. However, this might not be completely effective. To estimate the inflation of genome-wide SNP statistics caused by remaining population stratification, we compare the intercepts obtained from LD Score regression[67] to the mean χ^2 statistics. (At the meta-analysis stage, we also use these intercepts to deflate the SNP test statistics; for details see **Supplementary Information 3.4**) The ratio $\frac{Intercept-1}{\overline{X^2}-1}$ describes the share of the inflation in the mean χ^2-statistic ($\overline{X^2}$) that is due to population stratification.

We find that the intercepts from the LD Score regressions (performed for the meta-analyzed results of all cohorts) are close to unity (all < 1.009, **Supplementary Table 5.1**), indicating that the results of our GWAS are not likely influenced by issues related to population stratification.

When comparing these intercepts to the mean χ^2-statistics, we find that the share of inflation in the mean χ^2 that can be explained by population stratification is 5.63% (*SE* = 3.9%) for FAT, 2.6% (*SE* = 4.6%) for PROTEIN, 1.6% (*SE* = 3.4%) for SUGAR, and 3.69% (*SE* = 3.0%) for CARBOHYDRATE. Note that none of these estimates can be statistically distinguished from 0%.

These results indicate that the contribution of population stratification to the inflation of GWAS test statistics is likely to be small. This in turn suggests that a true polygenic signal is likely to be the main driver of the observed inflation of the chi-square statistics.

5.2 Summary of the GWAS findings

In the GWAS of the four dietary intake phenotypes, we detect 36 genome-wide significant loci: six independent loci for FAT; seven independent loci for PROTEIN; 10 independent loci for SUGAR; and 13 independent loci for CARBOHYDRATE (**Main Table 1** and **Supplementary Table 5.2**). We list the imputation quality of SNPs that reach genome-wide significance across cohorts in **Supplementary Table 5.3**. Several of these loci overlap across phenotypes – in some cases, even the lead SNP itself is identical across phenotypes (**Supplementary Table 5.4**). After taking this overlap into account, we identify 21 loci that are independently associated loci across phenotypes. These 21 loci include two loci harboring independent lead SNPs ($r^2 < 0.1$) that are in close vicinity (distance ≈ 1 Mb).

The SNP effect sizes range from 0.015 to 0.098 phenotypic standard deviations per effect allele, and the explained phenotypic variance (incremental R^2, i.e., coefficient of determination) ranges from 0.011% to 0.054%. The explained phenotypic variances for the top 50 independent lead SNPs for each of our four phenotypes are displayed in **Extended Data Figure 5**, where they are compared to the top 50 independent lead SNPs from other phenotypes (years of education, body mass index, waist-hip ratio adjusted for BMI, and height). Note that these sets of lead SNPs are phenotype-specific (i.e., *not* the same set of 50 SNPs across phenotypes). Here, we see that the explained variances for the diet composition lead SNPs are generally lower than those for BMI and height, slightly smaller than those for waist-hip ratio, and most comparable to those for years of education. (We note that effect sizes of SNPs do not necessarily scale with biological relevance. For instance, beneficial hypercholesteremia and schizophrenia drugs target genes whose common genetic variants have small effect sizes[71,72]. Small effect sizes are not unexpected, as most mutations with large effect sizes tend to be filtered out by evolutionary processes[73]. For dietary intake, knockout mouse studies of *FGF21* show a marked effect on sugar intake[48,49], although the GWAS effect sizes in humans are rather small in this locus. Hence, drugs that target *FGF21* (which are currently on trial for obesity and type 2 diabetes[74]) could have a substantial effect on sugar intake, even though GWAS effect sizes are small.)

We now highlight some notable specific findings (ordered according to chromosome and genomic location):

(1) The lead SNP for PROTEIN located intronically in *GCKR* is in very high LD with a *GCKR* missense variant ($r^2 = 0.935$) that is strongly associated with blood glucose levels[75,76], triglycerides, cholesterol[77–82], and type 2 diabetes[83]. *GCKR* codes for glucokinase regulatory

protein, which is in a family of enzymes vital for carbohydrate metabolism. It is unclear why it is associated with protein intake only. Since the *GCKR* lead SNP does not even reach (suggestive) significance for the other macronutrients (**Supplementary Table 5.4**), it seems unlikely that the association between protein and *GCKR* is driven by a protein—carbohydrate/fat/sugar substitution effect. Future research is therefore needed to study GCKR's possible role in protein taste-preference or protein metabolism.

(2) We identify an intronic variant in *KLB* (located on chromosome 4, associated with PROTEIN), which codes for klotho beta, and interacts with *FGF21* (located on chromosome 19)*,* the sweet and alcohol taste preference gene[84]. *KLB* SNPs were already identified for diet composition in previous GWAS[11,12]. FGF21 function depends on the availability of KLB; KLB acts as an essential co-receptor to FGF21[85,86]. Recent research into the effects of FGF21 function used brain-region-specific deletions of KLB to gain insight into the FGF21 tissues of action[49]. *KLB* has also been associated with alcohol consumption in previous GWAS[87]. Interestingly, the lead SNP was only associated with PROTEIN in our study, with *P* values > 10^{-5} for the other three diet composition phenotypes. In addition, MAGMA analyses point to another gene that interacts with *FGF21*: *MLXIPL*, which codes for a protein also known as Carbohydrate Response Element Binding Protein (chREBP) and is associated with FAT (**Supplementary Table 5.6**). MLXIPL acts as a transcription factor to FGF21. *FGF21, KLB,* and *MLXIPL* are located on different chromosomes (19, 4, and 7, respectively). Our results may imply that different genes of the same biological pathway might regulate different macronutrient preferences.

(3) The polymorphic missense variant in *ADH1B*, which codes for the alcohol dehydrogenase 1B enzyme (MAF = 0.029), is associated with FAT. The T-allele of this variant decreases the enzyme's ability to metabolize alcohol. The direction of effect is discordant; the allele that is associated with increased dietary fat intake is associated with a decreased conversion rate of alcohol into acetaldehyde, thereby increasing alcohol "sensitivity" and limiting an individual's capacity to consume alcohol. ADH1B also has the capacity to metabolize lipid products such as hydroxysteroids (e.g., cholesterol) and lipid peroxidation products, which might also explain its link to dietary FAT intake. Accordingly, *ADH1B* is not only highly expressed in the liver, but also in adipose tissues (subcutaneous and visceral, and in the breast) according to the GTEx[88] v7 portal. We find that the association between *ADH1B* and FAT virtually disappears in a sample of UKB individuals (*N* = 79,192, **Supplementary Table 5.7**) who reportedly do not consume alcohol. That is, the SNP *P* value reduces to *P* = 0.037, the semi-standardized beta decreases from 0.118 (95% CI [0.097, 0.139]) in the full UKB to 0.044 (95% CI [0.003, 0.086]) in the sample of alcohol abstainers. This implies that individuals who derive relatively more energy from alcohol derive relatively less energy from fat, which may be a consequence of our phenotype construction (see **Supplementary Information 2.8** for detailed explanation). In **Supplementary Information 11.2.3**, we also explore the possibility that the diets of former drinkers differ in composition from current or never drinkers, after taking calories from alcohol into account.

(4) An intergenic variant located between *CYP1A1* and *CYP1A* (MAF = 0.26) is associated with CARBOHYDRATE intake. These two genes, which are exclusively expressed in the liver according

to GTEx[88] v7 portal, are involved in caffeine metabolism[89,90]. The largest GWAS of coffee consumption to date also identified our CARBOHYDRATE lead SNP in this locus as its top association[90]. The T-allele of this variant is associated with decreased caffeine consumption. With respect to CARBOHYDRATE, the effect is discordant, implying that the allele that is associated with increased dietary carbohydrate intake is associated with decreased caffeine metabolism and consumption. While this finding might imply a biological role for *CYP1A* genes in carbohydrate metabolism, the results might also hint at shared lifestyle choices pertaining to food and caffeinated drinks.

(5) Two independent loci (tagging lead SNPs $r^2 < 0.1$) located in the middle of a large polymorphic inversion region on chromosome 8, for which the estimated breakpoints are 7,897,515 – 11,787,032[63,91] (GRch37), are associated with all four macronutrients. The base-pair locations for the four lead SNPs in this region are 9,173,209 (rs7012637); 9,173,358 (rs7012814); 9,183,358 (rs9987289) and 9,187,242 (rs1461729), and all are mapped to intronic regions of the same long, non-coding RNA (lincRNA) gene (*AC022784.6*), of which the biological function is unknown. However, a database of lincRNA genes indicates that this particular gene is highly constrained throughout evolution, with mutations in this site likely being deleterious[69].

The four lead SNPs in this locus (or SNPs in high LD with them; see **Supplementary Information 6.2.1**) have previously been associated with several blood phenotypes, including levels of cholesterol[79,92] and levels of glucose in pregnant women[75]. The true LD estimates in such a complex inversion region may be difficult to estimate[93], making it hard to conclude that this region actually carries two independently associated (and hence, potentially independently causal) variants for dietary intake. Other variants in this inversion region (which are not in LD with our lead SNPs) have previously been associated with neuroticism[63].

(6) We identify four variants in the intronic regions of *FTO* that are associated respectively with FAT, PROTEIN, SUGAR, and CARBOHYDRATE; each phenotype has its own individual lead SNP, all of which are in high LD with each other ($r^2 > 0.92$, MAF > 0.40. *FTO* is strongly associated with body mass index (BMI) and related anthropometric phenotypes[94,95], as well as type 2 diabetes[96], age at menarche[97], and breast cancer[98]. The causal BMI gene in the genomic region appears to be *IRX3*, which is regulated by variants in *FTO*[99,100]. A previous dietary intake GWAS has already identified GWAS hits in the *FTO* region[11]. With respect to BMI, the effects are concordant for FAT and PROTEIN, and discordant for SUGAR and CARBOHYDRATE, implying that the allele that increases BMI is associated with increased dietary fat and protein consumption, and decreased dietary sugar and carbohydrate consumption. These directions mirror the overall genetic correlations between BMI and dietary intake (**Supplementary Information 10**).

(7) We identify a genomic locus associated with CARBOHYDRATE that is located near a well-known large inversion polymorphism on chromosome 17[101], which notably includes the genes *MAPT* (microtubule-associated protein tau) and *CRHR1* (corticotropin releasing hormone receptor 1). *MAPT* is known to be involved in neurodegenerative disorders such as frontotemporal dementia, Alzheimer's disease and Parkinson's disease (disorders sometimes

summarized under the term "tauopathies"[102–105]). *CRHR1* may affect corticosteroid response in asthma patients[106,107]. The inversion polymorphism has also been associated with neuroticism[63].

(8) We identify a well-known polymorphic missense variant in *APOE* (MAF = 0.152) that is associated with FAT, SUGAR, and CARBOHYDRATE), which codes for apolipoprotein E, a protein that carries fatty acids through the bloodstream, and is involved in fatty acid metabolism. *APOE* is highly expressed in the liver and adrenals but also in the brain (it is the brain's principal cholesterol carrier[108]), where it is associated with the neurodegenerative processes related to Alzheimer's[109,110] and Lewy body disease[111]. The C-allele of the *APOE* missense mutation strongly predisposes to cognitive decline and Alzheimer's disease. We find that the effect is concordant with respect to SUGAR and CARBOHYDRATE and discordant with respect to FAT, implying that the allele that increases Alzheimer's risk is associated with increased dietary sugar and carbohydrate consumption, and decreased dietary fat consumption. However, the effects of this variant are greatly reduced in a sample of UKB individuals aged younger than 60 (N = 79,192), with P values > 0.01 and effect sizes generally only half as large (see **Supplementary Table 5.7**). This could imply that the *APOE* association is driven by a selection bias based on age (and therefore, Alzheimer's risk or status) of participants, since dietary intake also correlates marginally with age at the phenotype level. However, the 95% CIs for the effect sizes still largely overlap, making it hard to conclude whether the *APOE* associations are biased or indicative of biological relevance. The overall genetic correlations between dietary intake and Alzheimer's disease are small and non-significant, however (see **Supplementary Table 10.2**).

(9) Several variants located in a region spanned by Chr. 19: 49.21 – 49.27 Mb, which harbors genes that have been associated with sweet and alcohol taste preference in experimental mice and monkey studies[48,49]. Previous GWAS of dietary macronutrient intake had already identified variants in this locus[11,12]. Here, we find two independent loci in the *FGF21* gene, with additional lead SNPs in *MAMSTR* and *IZUMO1.* The two lead SNPs in *FGF21* gene are independently associated according to our locus definition ($r^2 < 0.1$, **Supplementary Table 5.4**). They are the SNPs rs838133, associated with FAT, PROTEIN, SUGAR and CARBOHYDRATE, and rs62132802, associated with SUGAR at genome wide significance, and with FAT, PROTEIN and CARBOHYDRATE at suggestive significance (with $P < 4.7 \times 10^{-7}$). Different genes in this locus (*MAMSTR, IZUMO1* and *FGF21*) harbor three different lead SNPs associated with FAT, SUGAR/CARBOHYDRATE, and PROTEIN. However, these three lead SNPs reach genome-wide significance for all phenotypes and are all in relatively strong LD with each other (r^2 = 0.803, 0.617, and 0.763), which might imply that the different lead SNPs tag the same biological effect for all phenotypes, despite their differing locations across genes. Thus, any conclusions that *MAMSTR* is the causal gene for FAT, *IZUMO1* is the causal gene for SUGAR/CARBOHYDRATE, and *FGF21* is the causal gene for PROTEIN are not justified by our results.

Seven of the remaining independently associated loci have not been associated with any other phenotypes in previously published and catalogued GWAS studies (**Supplementary Information 5.5**), and are located in or near genes that have not been studied in depth to date (these include the protein-coding genes *CTNNA2, SNORD66, MAD1L1,* and *L3MBTL4*). The other three loci have been associated with BMI, information processing speed, and alcohol

consumption (**Supplementary Information 6**). Across the 21 independently associated loci, seven lead SNPs are in (or when intergenic, nearest to) lincRNA genes that have no protein product and no published study on their biological function.

5.3 MAGMA gene-based analysis

We perform MAGMA gene-based analysis to assess the number of genes that reached “genome-wide significance” based on our meta-analysis results, where the *P* value threshold to declare genome-wide significance here is based on the number of *genes* tested (rather than the number of independent SNPs tested). This greatly reduces the multiple testing burden and could reveal statistically associated genetic loci that do not harbor statistically significant SNPs due to insufficient statistical power.

All the SNPs from our summary statistics that are located between the transcription start- and stop-sites of a gene annotated to that gene, based on NCBI 37.3.13 gene definitions. The implementation of this gene annotation is a limitation of MAGMA, as genes are also regulated by genetic variants that lie outside of the gene itself. MAGMA first calculates a per-gene test statistic (based on SNP summary statistic data) as the mean of the GWAS $-\log_{10} P$ values for all the SNPs between the transcription start and stop sites of a gene. MAGMA then calculates a *P* value for the resulting gene test statistic, using a procedure that takes into account LD between SNPs[112]. We use our main reference panel (described in **Supplementary Information section 3.3.1**) for LD estimations. MAGMA does not correct for LD *between* genes, which can result in several genes in the same genomic locus reaching statistical significance because they are in LD with each other. Bonferroni correction is applied to account for multiple testing, counting each gene as an “independent” test. Since genes are often (partially) in LD with each other, this is a conservative correction for multiple testing.

5.3.1 MAGMA results

The results are reported in **Supplementary Table 5.5** and **Extended Figure 6.** After Bonferroni correction, we discover 28 significant genes for FAT, 18 significant genes for PROTEIN, 27 significant genes for SUGAR, and 41 significant genes for CARBOHYDRATE. After considering overlapping genes between the phenotypes, we can report 81 uniquely associated “MAGMA genes.”

Curiously, all phenotypes have the exact same top two genes (located in the sweet taste preference locus on Chr. 19: 49 Mb): *RASIP1,* followed by *MAMSTR.* None of the lead SNPs for any of the diet composition phenotypes are located in *RASIP1,* but for all phenotypes, *RASIP1* is by the far the most significant MAGMA-gene in this locus. We do not know if this is indicative of true biological relevance or merely a statistical artifact. Although MAGMA corrects for gene length, the test for *RASIP1* is based on the larger number of SNPs, while *FGF21* has the smallest number of SNPs; it may be that the effect of *RASIP1* is simply measured with more precision. The sweet taste preference gene *FGF21* is the 6th most strongly associated gene for FAT, the 3rd for PROTEIN, the 4th for SUGAR, and the 5th for CARBOHYDRATE. While three additional genes (*FUT1, FUT2* and *IZUMO1*) in that locus are significant for all phenotypes, a seventh gene located there (*NTN5*) is only significant for FAT and SUGAR (and by far not associated with

PROTEIN and CARBOHYDRATE). Other significant genes in this locus are *BCAT2* (for PROTEIN) and *FAM83E* and *SPHK2* (for FAT).

MAGMA also identifies genes in loci that do not (yet) have a GWAS hit for any of the four phenotypes. This means that none of these genes are near (>1 Mb) any of the GWAS lead SNPs. For FAT, MAGMA identifies eight new loci; for PROTEIN, four; for CARBOHYDRATE, 15; and for SUGAR, ten. Taking overlap between phenotypes into account, MAGMA identifies a total of 32 new loci. A notable identified gene for dietary intake is *MLXIPL*, which codes for a protein that is known as Carbohydrate Response Element Binding Protein (chREBP). MAGMA identified this gene for FAT only. *MLXIPL* acts as a transcription factor to *FGF21. FGF21, KLB,* and *MLXIPL* are located on different chromosomes (19, 4, and 7, respectively), reflecting the fact that genes that interact with each other do not need to be located on the same chromosome. Our results may imply that different genes of the same pathway might regulate different macronutrient preferences.

5.4 Overlap between loci

As already noted in our summary of GWAS results (**Supplementary Information 5.2**), we find that several GWAS loci areassociated with more than one macronutrient. This is expected given the non-zero phenotypic and genetic correlations between macronutrients, as described above in **Supplementary Information 7**. In **Supplementary Information 2.8,** we also provide more context on the interpretation of these shared loci.

In this section, we check all diet composition lead SNPs for association more than one macronutrient. The results are summarized in **Supplementary Table 5.4**, which displays the lead SNPs' *P* values, effect sizes, and explained variances for all four macronutrients, as well as previous GWAS Catalog associations for SNPs in this locus. We also report the LD estimates (r^2) for the independent lead SNPs that are located near each other (≈ 1 Mb).

Among the 21 independently associated loci, we find that nine are represented by lead SNPs that are exclusively associated with one macronutrient, with none of the lead SNPs reaching suggestive significance ($P > 1 \times 10^{-5}$) for any other macronutrient. Six loci contain at least one lead SNP that reaches genome-wide significance for more than one (but not all four) macronutrients, while two loci contain at least one lead SNP that reaches genome-wide significance for all four phenotypes (notably, the loci characterized by *FTO* and *FGF21,* respectively). Four of the 21 loci's lead SNPs only reach suggestive significance (P value $< 10^{-5}$) for at least one additional macronutrient.

Finally, we report the LD estimates for the two genomic loci that contain more than one independent lead SNP according to our $r^2 < 0.1$ threshold. That is, we find two independent lead SNPs for CARBOHYDRATE in a locus spanned by Chr. 8: 9.17-18 Mb. These two independent lead SNPs (rs7012637 and rs9987289) are in very weak LD with each other ($r^2 = 0.074$). The other set of closely colocated independent lead SNPs are associated with SUGAR and are found in a region spanned by Chr. 19: 49.21-27 Mb. These two lead SNPs (rs838144 and rs6213802) are also in very weak LD with each other ($r^2 = 0.078$). However, we note that these two loci (on

chromosome 8 and 19) are located in two large inversion polymorphisms that are potentially characterized by complex (and hard to measure) LD patterns. Existence of the first polymorphism was confirmed previously[63,113], whereas the chromosome 19 inversion polymorphism is based on preliminary and unpublished findings from the Estonian biobank (Esko and Gonzalez, unpublished work). This could imply that the "independent loci" found in these inversions are not actually independent and instead tag the same causal biological effect.

5.5 Overlap with traits in the NHGRI-EBI GWAS catalog

We assess if any of the lead SNPs and their "LD partners" ($r^2 > 0.6$, see **Supplementary Information 6.2.1**) has been associated with any other trait by querying them in the NHGRI-EBI GWAS Catalog[114] (https://www.ebi.ac.uk/gwas/, download date September 19, 2017). This is a database that catalogues and harmonizes information from SNP associations in published and peer-reviewed GWAS. Here, we only record SNP associations that reached genome-wide significance (P value $< 5 \times 10^{-8}$) in their original GWAS. While some of the findings were described in the summary of the GWAS (**Supplementary Information 5.2** and **Supplementary Table 5.4**), the full results are displayed in **Supplementary Table 5.6.** We shortly summarize them below.

5.5.1 Results

For FAT, we find 255 overlaps in the GWAS catalog with 44 unique SNPs, representing five individual loci (where a locus is defined by its lead SNP). Only one FAT locus did not surface in the GWAS Catalog with its lead SNP or any of its LD partners. As noted earlier, we find overlaps with alcohol consumption (and alcohol consumption related cancers, as driven by the *ADH1B* association), obesity (and related anthropometric constructs and diseases, as driven by the *FTO* association), fibrinogen levels, Alzheimer's disease and related brain-, blood-, and cognitive-phenotypes (as driven by the *APOE* association, which is also associated with body fat and diabetes), autoimmune diseases (especially inflammatory bowel disease), vitamin levels, and urinary and blood metabolite levels (as driven by the sweet taste preference associated *FGF21* locus). As mentioned earlier, we also find overlap with the two SNPs previously[11,12] identified for dietary macronutrient intake.

For PROTEIN, we find 274 overlaps in the GWAS Catalog with 43 unique SNPs, representing five individual loci. Two PROTEIN loci did not surface in the GWAS Catalog with its lead SNP or any of its LD partners. As noted earlier, we find overlaps with circulating lipids, C-reactive protein, lactate, uric acid, glucose, insulin, albumin, calcium, liver enzymes, and creatinine; blood cell phenotypes; inflammatory bowel disease; resting heart rate; gout; height; kidney disease; and waist circumference (driven by the *GCKR* associated locus and the chromosome 8 inversion polymorphism locus), alcohol consumption (driven by the *KLB* associated locus), obesity (and related anthropometric constructs and diseases, driven by the *FTO* locus), and urinary metabolites, homocysteine levels, and skin disease (as driven by the sweet taste preference associated *FGF21* locus). As mentioned earlier, we also find overlap with the two SNPs previously identified for dietary macronutrient intake.

For SUGAR, we find in the GWAS Catalog 233 overlaps with 39 unique SNPs, which are driven by five individual loci. This means that five SUGAR loci did not appear in the GWAS Catalog. As noted earlier, we find overlap at obesity (and related anthropometric constructs and diseases, driven by the *RARB* locus and the *FTO* locus), Alzheimer's disease and related brain-, blood-, and cognitive-phenotypes (as driven by the *APOE* association, which is also associated with body fat and diabetes). As mentioned earlier, we also find overlap with the two SNPs previously identified for dietary macronutrient intake.

For CARBOHYDRATE, we find 302 overlaps with 70 unique SNPs, representing 10 individual loci. Three CARBOHYDRATE loci did not appear in the GWAS Catalog. Aside from associations with obesity (and related anthropometric constructs and diseases, driven by the *RARB* locus and the *FTO* locus) and Alzheimer's disease and related brain-, blood-, and cognitive-phenotypes (as driven by the *APOE* association, which is also associated with body fat and diabetes), we find overlap at fibrinogen-, liver enzyme-, glucose-, C-reactive protein-, and lipid-levels (driven by the chromosome 8 inversion polymorphism locus), caffeine and coffee consumption and metabolism, and with Parkinson's disease, intracranial volume, male pattern baldness, ovarian cancer, bone density, and blood cell phenotypes (driven by the chromosome 17 inversion polymorphism locus). As mentioned earlier, we also find overlap with the two SNPs previously identified for dietary macronutrient intake.

5.5.2 Discussion

This widespread overlap with diet composition and other (sometimes seemingly unrelated) traits could be caused by different scenarios. One scenario is pleiotropy, which means that a gene can be involved in different biological processes, causing the gene to be associated with different traits. Pleiotropic effects can depend, for instance, on the tissue in which the gene is expressed. It could also be caused by linkage between genes with disparate functions, where this linkage is merely causing a statistical (but not biologically causal) association between a trait and different genes in a genomic locus. Two traits may also overlap via mediation, with genetic variants affecting a trait (e.g., obesity susceptibility) having phenotypic downstream effects on another trait (e.g., cardiovascular disease). Finally, the overlap between two traits may be spurious, caused by a third, unmeasured trait that is associated with two overlapping traits. Note that genetic overlap at a small number of loci does not have to imply shared overall genetic architecture. This phenomenon was exemplified by two different autoimmune disorders that share several genome-wide significantly associated loci but a non-significant overall genetic correlation[115]. In this sense, the genetic correlations reported in **Supplementary Table 10.2** may be more informative in assessing potential for shared etiology between traits. However, the trait associations for the individual loci can still be helpful in understanding potential individual gene functions.

6 Biological annotation of GWAS findings

In order to gain insight into the biological architecture of diet composition, we employ several different strategies. First, we annotate the "genome-wide" architecture of diet composition by

partitioning the genetic signal from our summary statistics into several different types of functional categories. This is achieved with stratified LD Score regression, which assesses from which functional categories the polygenic GWAS signal emerges. We assess three broad types of functional categories: 1) different genomic annotations from the "baseline model"; 2) tissue types as ascertained with chromatin (histone-mark) data; 3) tissue types as ascertained with specifically expressed genes in GTEx data.

To annotate the top GWAS findings (both in terms of SNPs and in terms of genes), we perform MAGMA gene-based analysis, a hypothesis-free test that uses GWAS summary statistics to assess which protein-coding genes reach genome-wide significance. To understand the biological functions of the genes that were significant in any of the four diet composition phenotypes, we then query them in the Gene Network database, which assigns genes' predicted functions on the basis of publicly available co-expression information.

To annotate the lead GWAS SNPs, we query whether they (or any of their LD partners) are associated with gene expression in relevant GTEx tissues, or whether they are in LD with any SNPs that have protein-altering consequences.

6.1 LD Score partitioning of heritability

6.1.1 General method

We examine the enrichment of functional genomic annotations of our GWAS results by partitioning our phenotypes' polygenic signals into different functional categories. These annotations describe in which tissue or cell type diet composition SNPs are likely to be expressed, or in which genomic classes the diet composition SNPs are likely to fall (e.g., exonic or intronic, promoter or enhancer regions, evolutionarily conserved regions, etc.). We do so using stratified LD Score regression[116], which is based on the following moment condition:

$$E[\chi_j^2] = N \sum_{c=1}^{C} \tau_c l(j,c) + Na + 1,$$

where χ_j^2 is the chi-square statistic for SNP j from a GWAS, N is the sample size of the GWAS, c indexes functional categories that are not necessarily disjoint, $l(j,c)$ is the stratified LD Score of SNP j with respect to functional category c, τ_c is the average contribution to heritability of a SNP due to its membership in category c, and a is a term that measures confounding biases such as population stratification.

Finucane et al.[116] proved that under a set of assumptions, the explained proportion of heritability per functional category can be estimated with a regression analogous to the equation above. Enrichment for a particular functional category is then defined as the fraction of heritability due to that category, divided by the fraction of SNPs in the category. However, the heritability due to SNPs in the category is simply the sum of their squared effects. Since SNPs may belong to more than one functional category, enrichment for a particular category may be driven by overlapping enrichment at another category. In this analysis, we therefore rely more strongly on the statistical significance of the regression coefficients (τ_c) from the stratified regression analysis, as these

coefficients are corrected for the "baseline model" described below. Note that the tissue categories (both those based on chromatin data and GTEx gene expression data) are also only corrected for the baseline model, meaning that significance of the regression coefficients can therefore still be driven by signal coming from overlapping tissues.

We conduct separate analyses, using three difference sets of reference data. First, we used the Finucane et al.[116] "baseline model" that involves 24 main functional annotations of interest, plus a number of ancillary annotations formed by extending 500-kb windows around SNPs that qualify for the main annotations. These windows are included to control for spurious association caused by SNPs flanking the functional annotations. This results in a total of 52 tissue annotations. The baseline model includes annotations such as different histone modifications; promotor or enhancer regions; transcription start sites; repressed regions; 3' and 5' UTR regions; DNAse hypersensitive sites; protein coding regions; intronic regions; evolutionarily conserved regions (which captures genomic regions that are observed in 29 different mammal species[117]); and more. Several of these annotations are defined more than once by means of different reference data sources.

We then perform two separate analyses, which used tissue-level annotations that were based, respectively, on i) histone-mark demarcations (henceforth referred to as "chromatin data")[116] and ii) gene expression data from the Genotype-Tissue-Expression (GTEx) Project[88,118]. The chromatin annotations from Finucane et al.[116] are based on ChIP-seq data, which ascertains whether SNPs are located in or near histone marks that are associated with increased gene expression. Finucane et al.[116] grouped several tissues and cell types into 10 broad tissue types: Adrenal/Pancreas, Cardiovascular, Central Nervous System, Connective/Bone, Gastrointestinal, Immune, Kidney, Liver, Skeletal Muscle, and Other.

The GTEx-based annotations provided by Finucane et al.[118] span 53 tissues. Finucane et al. named this new implementation of partitioned LD Score regression "LDSC-SEG", short for "LD Score regression applied to specifically-expressed genes." To this end, they identified sets of genes that are specifically expressed in the individual tissues. SNPs located in one of these genes or within 100 kb of either endpoint were assigned the tissue-level annotation. These tissue annotations based on GTEx are far more specific than the annotations based on chromatin data since, for example, they split the brain into 15 different tissue types, whereas the chromatin data only designates a broad "Central Nervous System" term.

For both the chromatin and GTEx tissue analyses, we also include the baseline annotations as control covariates. As a reference panel for calculating LD scores, we use European-ancestry samples in the 1000 Genomes Project and conducted the analysis on HapMap3 SNPs. We address the autocorrelation and heteroscedasticity issues inherent in running regressions using LD Scores by weighting the regression with LD Scores. We follow the recommendation of Finucane et al. to exclude HapMap3 SNPs in the HLA region.

6.1.2 Results

LD Score partitioning of heritability according to functional genomic categories

The results are displayed in **Supplementary Table 6.1**). For all of our four phenotypes, the baseline annotation "Conserved" is the only annotation that has τ_c that is distinguishable from zero after Bonferroni correction, with a corresponding 25.5-fold (*SE* = 4.4) enrichment factor for FAT, 19.8-fold (*SE* = 4.4) enrichment factor for PROTEIN (*SE* = 4.4), 26.3-fold (*SE* = 3.7) enrichment factor for SUGAR and 25.2-fold (*SE* = 3.4) enrichment factor for CARBOHYDRATE. All these enrichment factors are distinguishable from zero. They correspond to explaining 66.5% (*SE* = 11.5%) of heritability for FAT, 51.6% (*SE* = 11.6%) for PROTEIN, 38.7% (*SE* = 9.7%) for SUGAR and 65.7% (*SE* = 8.8%) for CARBOHYDRATE.

LD Score partitioning of heritability according to 10 chromatin-demarcated tissues

For all our phenotypes, the coefficient for "Central Nervous System" category is distinguishable from zero, with enrichment factors of 2.97 (*SE* = 0.53) for FAT, 3.67 (*SE* = 0.51) for PROTEIN, 2.96 (*SE* = 0.46) for SUGAR, and 3.21 (*SE* = 0.39) for CARBOHYDRATE (**Supplementary Table 6.2**). This corresponds to explaining 44.1% (*SE* = 7.9%) of the heritability for FAT, 54.6% (*SE* = 7.5%) for protein, 44.1% (*SE* = 6.8%) for SUGAR, and 47.7% (5.8%) for CARBOHYDRATE. Only one other tissue-level partition is enriched in any of our four phenotypes: for FAT, "Adrenal/Pancreas" has a significant regression coefficient, with a 3.98 (*SE* = 0.71) enrichment factor, representing 37.3% (*SE* = 6.7%) of explained heritability for FAT.

LD Score partitioning of heritability according to 53 GTEx tissues

All functional categories with regression coefficients that are distinguishable from zero in the LDSC-SEG analyses are brain regions, with FAT having the most enriched regions in 10 brain regions, while CARBOHYDRATE had the fewest at zero, whereas brain tissues only reached marginal significance after Bonferroni correction (**Supplementary Table 6.3**). For FAT we find significance for the amygdala, anterior cingulate cortex, caudate, cortex, frontal cortex, hippocampus, hypothalamus, nucleus accumbens, putamen (basal ganglia), and substantia nigra. Interestingly, PROTEIN also shows significance for the hypothalamus and substantia nigra but is significant in two regions where FAT is not: cerebellum and cerebellar hemisphere. SUGAR shows significance for the amygdala, anterior cingulate cortex, cortex and frontal cortex.

6.1.3 Discussion

First, we find that evolutionarily "conserved regions" (across 29 mammal species[117]) is the only significant genomic annotation in the baseline model. While this could hint at evolutionary selection for dietary behavior, we note that the "conserved" category was the most significantly enriched annotation across a range of health and behavioral phenotypes in Finucane et al.[116], indicating that mutations in evolutionarily conserved regions affect a wide variety of phenotypes.

As for the tissue categories, we find the most consistent evidence for involvement of the brain, as seen in both the chromatin and GTEx analyses. While the chromatin data also indicated a role for Adrenal/Pancreas for FAT, this was not replicated with the GTEx data. This could mean that the Adrenal/Pancreas tissues are too broadly defined in the chromatin data (or overlap too much with

SNPs annotated to other regions), or that the GTEx data are underpowered to capture specifically expressed genes in adrenal or pancreatic regions.

With regards to the brain tissues, we find that each diet composition phenotype seems to have its own pattern of associated brain regions. The exception is CARBOHYDRATE, for which we find no specific brain region enrichment after Bonferroni correction, although the chromatin data do indicate a role for the central nervous system. We anticipate that specific brain tissues will start reaching significance with larger GWAS sample sizes. For FAT, PROTEIN and SUGAR, the confidence intervals of the effect sizes largely overlap, hampering conclusions on the specificity of involved brain regions. The brain-region-specific results are also hard to interpret due to probable overlap in gene expression across brain regions. For this reason, LDSC-SEG also offers reference files for the 14 brain regions, where sets of specifically-expressed genes were ascertained by comparing brain regions to each other – as opposed to comparing brain regions to the rest of the bodily tissues. However, we are underpowered to perform these analyses. GTEx does not cover all parts of the brain (with regions in the parietal, occipital and temporal lobes largely missing) and is based on small donor sample sizes, stressing the need for replication of these findings in independent samples. Finally, we stress that the LDSC-SEG analyses only found significant involvement of brain regions and no other bodily tissues (even before Bonferroni correction). Together, these analyses highlight the importance of the brain for macronutrient intake.

6.2 Annotation of genome-wide significant genes and loci

To gain insight into the biological architecture of the genome-wide significant loci, we query the lead SNPs and their LD partners in several databases: 1) in Haploreg v4[119,120], which displays dbSNP information for SNPs' protein-coding status; 2) in the GTEx eQTL database v6p[88,121], which records whether SNPs are associated with expression of any measured gene transcript in 53 different tissues. We also query the lead SNPs and their LD partners in the NHGRI GWAS Catalog, as described in **Supplementary Information 5.5**. Finally, we apply MAGMA gene-based analysis, which tests the GWAS summary statistics for significance of 18,224 protein-coding genes, greatly reducing the multiple-testing burden compared to GWAS of individual SNPs. We then query the significant "MAGMA-genes" from that analysis in the Gene Network[122] database, which predicts gene functions on the basis of co-expression patterns and summarize the most frequently occurring predicted functions.

6.2.1 Definition of LD partners

In accordance with previous SSGAC projects[63,64,91], we create a list of "LD partners" for each phenotype's lead SNPs. It is important to consider these LD partners and not just the lead SNPs themselves, because the lead SNPs are not necessarily the causal variant in the locus, and the lead SNPs may not be present in the GTEx database or studies included in the GWAS Catalog (while some of their LD partners might be). Thus, the SNPs in high LD with the lead SNP may tag or represent biologically relevant effects.

Here, we define LD partners as SNPs that are in *high* LD with the lead SNP. This contrasts with the locus definition we used for the determination of independent lead SNPs in **Supplementary**

Information 3.3.4, which uses a relatively low r^2 threshold ($r^2 > 0.1$). This is necessary, as SNPs weakly correlated with the lead SNP may only be significant because of this correlation and not because they tag an independent biological effect. This might especially be an issue in larger GWAS sample sizes. Thus, for the annotation of the biological functions of lead loci, we do not consider such weakly correlated SNPs, as they are unlikely to capture biological effects that are relevant for diet composition. We therefore define the lead SNPs' LD partners as being in at least moderate LD with the lead SNP ($r^2 > 0.6$) and located less than 250 kb from the lead SNP (both upstream and downstream, representing a 500-kb window around lead SNP). We perform these LD calculations in our main reference panel described in **Supplementary Information 3.3.1.**

6.2.2 dbSNP protein-altering status

We query all diet composition lead SNPs and their LD partners in HaploReg v4[119,120] (downloaded June 7, 2016), a database that displays dbSNP protein-coding status of SNPs. Protein-altering ("nonsynonymous") SNPs are SNPs that change the amino acid (and therefore, protein) composition of a gene product. HaploReg defines four different protein-altering categories: "missense," representing a mutation which results in a different amino acid being coded; "nonsense," representing a mutation which results in a stop-codon being coded, marking an abrupt end in the gene product (these variants are also known as "truncating" variants); "frameshift," representing a mutation that changes the reading frame of a gene, resulting in the codons being translated in a completely different way; and "splice site donors" and "splice site acceptors," representing mutations that change the way a gene is spliced. The latter three variant classes are sometimes referred to as "loss of function" variants[123], as they can have severe consequences on gene functionality.

The results are displayed in **Supplementary Table 6.4**. We find 34 unique protein-altering variants in LD with the lead SNPs (in two cases, the lead SNP itself is the protein-altering variant). Among these, 32 variants were missense, and 2 variants re nonsense. Notable variants are (as described earlier in **Supplementary Information 5.2**) the well-known missense mutations in *APOE, ADH1B, MAPT,* and *GCKR.* In the sweet- taste preference locus on Chr 19: 49 Mb, we find that the lead SNPs for all four phenotypes are in LD with a missense mutation in *RASIP1* (a gene involved in vasculo- and angiogenesis according to Gene Ontology)*,* while PROTEIN, SUGAR and FAT are in LD with a missense variant in *IZUMO1* (an essential gene for sperm-egg fusion[124]), and FAT is in LD with a nonsense and a missense variant in *FUT2* (a gene involved in sugar metabolism that protects against viral infection[125–127] and is associated with gut microbiome composition[128]).

On Chr. 17: 43 Mb, the lead SNP for CARBOHYDRATE is in LD with missense variants in *MAPT* (a gene associated with Alzheimer's disease, where seven distinct missense variants are in LD with the lead SNP) as well as with *STH*, *KANSL1* (a gene involved in the regulation of gene expression), *SPPL2C* (a gene almost exclusively expressed in the testis), and a nonsense variant in *CRHR1* (i.e., corticotropin releasing hormone receptor 1, an essential regulator for cortisol homeostasis; among other physiological effects, cortisol is a hormone that is essential in the stress-induced "fight or flight" system, where it enhances glucose availability in the bloodstream).Finally, we find two missense mutations in LD with the lead SNP for

CARBOHYDRATE. Both are in *CCDC171*, a gene involved in regulation of gene expression according to Gene Ontology.

6.2.3 GTEx gene expression eQTLs

In this section, we report whether any of our lead SNPs (or their LD partners) are associated with gene (transcript) expression in the GTEx (Genotype-Tissue expression) portal[88]. A SNP that is associated with gene (transcript) expression is called an "expression quantitative trait locus" (eQTL). We do this to gain insight into the potential functional consequences of these SNPs. Gene expression is important since the vast majority of GWAS lead SNPs for human complex traits lie in non-coding regions[129]. Thus, most SNPs important for human health and behavior are likely to exert their effect via altered gene expression rather than altered protein composition.

The publicly available data from the GTEx project are based on RNA-sequencing results from 53 different human tissues and cell types from postmortem donors. By using RNA-sequencing, GTEx could measure RNA levels of protein-coding genes, as well as RNA levels that are only involved in *regulation* of gene expression (e.g., RNA transcripts encoded by long noncoding RNA genes). In order to establish statistically significant eQTLs (i.e., expression quantitative trait loci, which are SNPs statistically associated with transcript expression) in these tissues, GTEx performed an elaborate two-step false discovery rate correction[88]. Here, we report only eQTLs (for the lead SNPs and their LD partners) that were significant at $P < 0.05$ after this false discovery correction. We downloaded the GTEx v6 eQTL data on December 13, 2016.

Prior to examining our GWAS results, we selected a number of tissues we deemed of *a priori* relevance for diet composition. These are: all adipose tissue, adrenal gland, all nervous (i.e., brain, pituitary, and tibial) tissue, all colon and intestinal tissue, liver, skeletal muscle, pancreas, stomach, thyroid, and whole blood. The donor sample sizes for these tissues ranged from $N = 72$ (for anterior cingulate) to $N = 361$ (for skeletal muscle). Note that these sample sizes are rather small, implying relatively low statistical power for discovery of all relevant eQTLs. After performing the stratified LD Score regression for our GWAS results, however, which relied on epigenetic histone mark data, we only find strong *ex post* evidence for enrichment of brain- and possibly adrenal/pancreas tissue (**Supplementary Information 6.1)**, so we mainly focus our written summary of eQTL findings here on the pancreas tissue and the tissues of the central nervous system. The eQTL findings for the other tissues described above (e.g., skeletal muscle, adipose, etc.) can still be found in **Supplementary Table 6.5**.

The results are displayed in **Supplementary Table 6.5**. We found a total of 62 unique "eQTL genes" across the four phenotypes and across all included tissues. These "eQTL genes" are unique transcripts that were associated with our lead SNPs or SNPs in strong LD with them. These included 37 eQTL genes that could be mapped to genes with existing gene symbols, indicating that these are genes with known gene products. If we consider the same eQTL gene (i.e., gene-SNP pairing) occurring in two different tissues as two separate eQTLs genes, we find 381 eQTL genes, of which 244 have defined gene symbols.

FAT. Across all tissues, we find a total of 20 eQTLs for FAT, representing nine unique eQTL genes, with six of those having gene symbols. Two of the eQTLs are in brain tissues, and one

eQTL is in adrenal tissue. The eQTLs are driven by three loci: the diabetes locus on Chr. 7: 1 Mb, the cholesterol locus on Chr. 8: 9 Mb, and the sweet taste preference locus on Chr. 19: 49 Mb.

PROTEIN. Across all included tissues, we find a total of 48 eQTLs for PROTEIN, representing 19 unique eQTL genes, with 12 of those having gene symbols. Three eQTLs were in adrenal tissue, 3 in pancreas tissue, and five in brain/pituitary tissue. Three of the brain eQTLs are in cerebellar tissues (all for gene transcripts with undefined gene symbols), while one is in hypothalamic tissue – these tissues were the only significantly enriched brain tissues for PROTEIN in the stratified LD Score regression analysis according to GTEx tissues. The eQTLs are driven by three loci: the diabetes locus on Chr. 2: 27 Mb, the alcohol consumption locus on Chr. 4: 100 Mb, and the sweet-taste preference locus on Chr. 19: 49 Mb.

SUGAR. Across all tissues, we find a total of 24 eQTLs for SUGAR, representing eight unique eQTL genes, with six of those having gene symbols. Out of these 24 eQTLs, 22 are driven by SNPs in the Chr. 19: 49 Mb locus. Interestingly, *FGF21* (the putative causal gene in this sweet taste preference locus[84]) itself was *not* an eQTL gene, while other genes in this area *IZUMO1, RASIP1, NTN5, SEC1P, MAMSTR* are, as well as several other gene transcripts with undefined gene symbols. These findings underscore that, as is well known, eQTL data cannot be used as conclusive evidence for causality, but also leaves the possibility that other genes in this area (in addition to *FGF21*) may also have causal effects. The other two eQTLs are the lead SNP in the Chr. 8: 9 Mb locus; one was a pseudogene, while the other one is a novel, unstudied gene with an undefined gene symbol.

CARBOHYDRATE. Across all genes, we find a total of 352 eQTLs for CARBOHYDRATE, representing 49 unique eQTL genes, with 30 of those having gene symbols. The eQTLs are mainly driven by the lead SNP rs36123991 and its LD partners, which has eQTLs in every single queried tissue; we find 306 eQTLs for these SNPs across tissues, accounting for 34 unique eQTL genes. This makes it difficult to pinpoint a single putative causal gene in this locus, but it indicates that the associated SNPs have widespread effects on gene expression.

6.2.4 Predicted functions of MAGMA genes in Gene Network

To gain insight into the functional architecture of the 81 significant MAGMA-genes identified across the four macronutrients (**Supplementary Information 5.3**), we query them in Gene Network (download date March 14, 2018). This database assigns genes with predicted functions on the basis of co-expression patterns. Across domains, we then count which functions surface most frequently. Gene Network uses information from expert-curated gene sets, and assess co-expression between all measured genes and these gene sets. If a gene is co-expressed with the genes in the gene set, the gene is assigned the predicted function that belongs to that gene set. (These co-expression data are also used as input in the DEPICT[130] software.) Note that we use this query of predicted gene functions as a way to gain rough insight into the putative biological functions of the MAGMA genes; this is not a formal test for statistical association. Moreover, it is important to keep in mind that the predicted functions are not independent, as MAGMA does not correct for LD between genes. The query therefore included several different genes from the

same locus, where it is possible that in every locus, only one of these queried genes was the actual causal one.

In a nutshell, Gene Network predicts gene functions on the basis of co-expression data, as described in detail in Fehrmann et al.[122] and Pers et al.[130], and in previous SSGAC projects[63,64,91]. Gene Network was updated in late 2017 with new gene expression and annotation data.

We record all Reactome pathways that were predicted to be relevant for the MAGMA genes at statistical significance (*FDR*-corrected *P* value < 0.05). We only record pathways that were predicted to be relevant in a positive direction (i.e., upregulated). All genes were available in Gene Network, with the exception of *DCDC5,* where more recent gene definitions from NCBI showed that this gene was merged with *DCDC1,* which was queried instead of *DCDC5.*

The results for the top ten most frequently occurring terms are displayed in **Supplementary Table 6.6**. Here, we briefly summarize the results. The results show a mix of basic pathways involved in signal transduction (needed for cell growth, division and communication, and thus essential for viability), the immune system, lipid metabolism, and neuronal development. The top most predicted Reactome pathway is a tie between three pathways, which were predicted for 38 (out of 81) genes: "Downstream signal transduction", "Signaling by EGFR", and "Signaling by PDGF", where the latter pathway is actually the mother pathway to "Downstream signal transduction." PDGF is short for platelet-derived growth factor, a basic growth factor needed for cell growth and division. EGFR is short for epidermal-derived growth factor which plays a role in various types of cancer. Several insulin-related pathways also occur: 31 genes have predicted functions for "IGF1R signaling cascade", "Insulin receptor signaling cascade", "IRS-related events triggered by IGF1R", and "Signaling by type 1 insulin-like growth factor 1 receptor IGF1R." With regards to neuronal development, we find that several genes have predicted functions for "Axon guidance" (31 genes) and several of its sub-pathways (31 genes for "L1CAM interactions" and 27 genes for "Semaphorin interactions"). With regards to metabolism, we find that several genes have predicted functions for pathways associated with lipid and energy metabolism (29 genes for "Fatty acid triacylglycerol and ketone body metabolism", 28 genes for "Integration of energy metabolism", 27 genes for "Glycerophospholipid biosynthesis", and 27 genes for "Phospholipid metabolism").

In conclusion, we find no distinct biological pattern for these 81 MAGMA-genes in the Gene Network database of predicted gene functions. This stands in contrast to the LD Score partitioned regression findings, which point strongly to a role for the brain. Here, it seems that the top GWAS genes do not have a distinct neural function, which might have been predicted on the basis of the GWAS Catalog findings in **Supplementary Information 5.5**, where we seem to find more overlaps with metabolic traits at these loci than with psychological or psychiatric traits.

6.3 Discussion of bioannotation results

In our bioannotation of the genome-wide biological architecture and individual SNPs and genes, two major patterns emerged. First, we find widespread enrichment of the brain for diet

composition. Second, we find some evidence for enrichment in the adrenal/pancreas (for FAT) in the tissue demarcations that were based on chromatin data, although this was not replicated with the GTEx tissue demarcations. We currently do not know if the adrenal/pancreas enrichment is a spurious finding or is not replicated due to lack of statistical power in the GTEx data.

For the GWAS SNPs and genes, we identify some interesting candidate loci and genes, as summarized in **Supplementary Information 5.2**, which might be valuable for future functional and fine mapping analyses. When the loci had been previously associated with another trait, this tended to be in GWAS of BMI, metabolic syndrome (e.g., dyslipidemia, fasting glucose and insulin) and diabetes. Most loci harbor a number of genes that were in LD with each other, making it difficult to point out candidate causal genes. Some loci re in large inversion polymorphisms associated with neurodegenerative disorders. Although many of the loci overlapped across the four phenotypes, some loci were (currently) only associated with one phenotype. Although our Gene Network query of MAGMA-genes is likely underpowered at this stage (with only 81 significant MAGMA genes to query), the MAGMA-genes did not clearly point to "brain genes", with many predicted functions for signal transduction (especially for insulin) and lipid metabolism in addition to pathways related to neuronal development. The GWAS for BMI showed a similar pattern, as the genome-wide biological architecture points to the brain[94,116], while the top gene locus (containing *FTO* and *IRX3*) seems to have a direct metabolic effect[100].

7 Genetic correlations between the phenotypes and sexes

In this section, we examine the genetic and phenotypic correlations between the four macronutrients, as based on the results from the full meta-analysis. We also report the genetic correlations between sexes.

7.1 Genetic correlations between macronutrients

The results are summarized in **Main Table 2**. The only positive genetic correlation is between SUGAR and CARBOHYDRATE ($\hat{r}_g$= 0.73, SE = 0.02), which is highly statistically distinguishable from both zero and unity. We find that all phenotype pairs have a negative genetic correlation that is statistically distinguishable from zero at the 0.1% level, except for FAT and PROTEIN ($\hat{r}_g$= -0.02, SE = 0.07) whose genetic correlation is negative but close to zero.

In terms of the magnitudes of the correlations, the largest negative genetic correlation is between FAT and CARBOHYDRATE ($\hat{r}_g$= -0.61, SE = 0.03), and FAT and SUGAR ($\hat{r}_g$= -0.51, SE = 0.04). They are followed by PROTEIN and SUGAR ($\hat{r}_g$= -0.31, SE = 0.06) and PROTEIN and CARBOHYDRATE ($\hat{r}_g$= -0.23, SE = 0.05).

Finally, we examine genetic correlations between the four macronutrients and SATURATED FAT (relative intake of saturated fat in the UKB), reported in **Supplementary Table 7.2**. SATURATED FAT has significant genetic correlations with all four macronutrients, including PROTEIN ($\hat{r}_g$= -0.121, P = 0.046). The latter is barely statistically distinguishable from zero at the 5% level. If

this genetic correlation is truly negative, it is surprising in light of the shared animal origin of protein and saturated fat (e.g., [131]). The genetic correlations between SATURATED FAT and the other macronutrients also all statistically distinguishable from 1 or -1 ($P < 4 \times 10^{-20}$), indicating that SATURATED FAT has a genetic architecture that is partially unique from relative consumption of other macronutrients, including all fats ($\hat{r}_g$= 0.643, SE = 0.039).In **Supplementary Information 10**, we also report genetic correlations between the four macronutrients and absolute alcohol consumption (number of drinks weekly, not corrected for total energy intake). We find negative genetic correlations between the diet composition phenotypes and absolute alcohol intake that are statistically distinguishable from zero. A slight exception to this is FAT, which was also genetically negatively correlated with alcohol, while its P value did not surpass the Bonferroni-corrected significance threshold.

7.1.1 Comparison to phenotypic correlations

We compare the genetic correlations to the phenotypic correlations in four different cohorts (UKB, HRS, FHS and WHI). The genetic correlations outlined above generally mirror their phenotypic correlations. The results (with their 95% confidence intervals) are reported in **Supplementary Table 7.3**.

Since sugars are a subset of carbohydrates, SUGAR and CARBOHYDRATE have a strong positive phenotypic correlation that is statistically significantly different from both zero (but also from one) in all cohorts (range: $\hat{r}_p$= [0.69, 0.79]).

All other phenotypic correlations are negative and statistically significant in all cohorts. In increasing order these range from CARBOHYDRATE-FAT (range: $\hat{r}_p$= [-0.79, -0.54]), SUGAR-FAT (range: $\hat{r}_p$= [-0.62, -0.41]), CARBOHYDRATE-PROTEIN (range: $\hat{r}_p$= [-0.39, -0.09]), SUGAR-PROTEIN (range: $\hat{r}_p$= [-0.30, -0.08]). When we compare these phenotypic correlations with genetic correlations from the previous section, the only genetic correlation that does not fall within its corresponding phenotype interval for these four cohorts is SUGAR-PROTEIN ($\hat{r}_g$ = -0.31).

7.2 Genetic correlations between sexes

Finally, we assess the genetic correlations between sexes for the four macronutrients in the UKB. The results are summarized in **Supplementary Table 7.4.** None of the point estimates are distinguishable from 1 (smallest r_g = 0.751, SE = 0.163 for PROTEIN). Hence, we currently do not find evidence that relative macronutrient intake has a sex-specific genetic architecture.

8 Estimation of SNP-based heritability

Previously, twin studies have shown moderate heritability for macronutrient ratios (h^2 = 27-70%)[132–134]. In this section, we employ GREML (implemented by GCTA[56]) and LD Score regression[67] to obtain empirical estimates of SNP-based heritability of diet composition, as represented by the four macronutrients. Previous SNP-based estimates of heritability for relative intake of fat, carbohydrates, and protein (% of total energy) have varied between 3.2 and 8%[12,69].

8.1 Methods

8.1.1 The GCTA method

The GCTA[56] method for estimating SNP-based heritability is based on restricted maximum-likelihood estimation and uses the genetic relatedness matrix (GRM) to estimate the SNP heritability. Under certain assumptions[56], the method yields unbiased estimates of SNP-based heritability. However, it is necessary to limit the number of SNPs and individuals in the analysis in order for it to be computationally tractable. To this end, we follow the procedure described in Karlsson Linnér et al.[51]. That is, we restrict the GCTA analysis to a random subset of 30,000 individuals in the UKB. To then obtain a set of unrelated individuals, we thereafter drop one individual in each pair of individuals with a cryptic relatedness exceeding 0.025, resulting in N = 28,635. The results are summarized in **Supplementary Table 8.1**. In total, 633,335 directly genotyped SNPs with MAF > 0.01 were included in the GCTA heritability estimation.

8.1.2 The LD Score regression method

Another method for obtaining SNP-based heritability estimates is LD Score regression[67]. The method is based on assumptions that imply that a SNP's GWAS χ^2-statistic is linearly related to its LD Score, with SNPs with higher LD Scores having a higher likelihood of association in a GWAS. A SNP's LD Score simply is the sum of the squared correlation coefficients between a SNP and all the other SNPs it is in LD with (as measured in a particular reference panel). The slope of the LD Score regression (i.e., of the SNPs' GWAS χ^2-statistics on their LD Scores) can then be rescaled to obtain an estimate of SNP-based heritability. In practice, LD Score regression is commonly viewed as giving a lower bound of heritability from common SNPs[135]. An advantage of LD Score regression is that it is calculated from the GWAS summary statistics and thus uses data from all individuals included in the GWAS.

The LD Scores used in this analysis were computed by Finucane et al.[116] with genotypes from the European-ancestry samples in the 1000 Genomes Project (the "eur_w_ld_chr" files). Subsequently, only HapMap3 SNPs with MAF > 0.01 are included in the LD Score regression, as these ~1.2 million SNPs are well-known to be imputed properly across individual cohorts. Of note, we do not apply GC to the summary statistics prior to estimating the LD Score regressions.

8.2 Results and discussion

The results of the heritability estimations are reported in **Supplementary Table 8.1** and displayed in **Extended Data Figure 7**. The GCTA-estimated heritability estimates range from h^2_{GCTA} = 0.021 (SE = 0.017) for PROTEIN to h^2_{GCTA} = 0.079 (SE = 0.018) for CARBOHYDRATE. The LDSC-estimated heritability estimates range from h^2_{LDSC} = 0.028 (SE = 0.002) for PROTEIN to h^2_{LDSC} = 0.047 (SE = 0.004) for SUGAR. These estimates are more similar in magnitude to behavioral traits such as educational attainment[64,66], subjective wellbeing[63] and risk preferences[91], than BMI and obesity[94,136]. While the estimates are small, they imply a non-ignorable biological component that affects dietary patterns.

It is important to note that the heritability estimates for diet composition may be affected by measurement error, caused by for instance the random noise induced by the 24-hour dietary recall measurement in UKB (**Supplementary Information 2.5.3**) and imperfect measurement (e.g., recall error, imperfect stability of dietary habits) of dietary intake in general. Second, there are generic reasons for the missing heritability we observe. Twin estimates of narrow-sense heritability might be overestimated for various reasons, including genetic nurture[137] and only partially capturing non-linear genetic effects[138]. Moreover, our estimates only capture variation due to measured genetic markers, while unobserved genetic markers (e.g., very rare variants or structural variants that are not well tagged by measured SNPs) might also contribute to the heritability of eating behaviors. Similar studies for traits like height and BMI suggest that this "still missing heritability" tends to disappear once fully sequenced genetic data is used[139]. However, we are limited to studying the data that is currently available.

Genetic analysis of a phenotype can be of interest even if the true heritability of a phenotype is low, for a variety of reasons. Perhaps most importantly, a genetic analysis may shed light on biological mechanisms. This is valuable in itself but could also provide knowledge that ultimately leads to new treatments. Even if naturally occurring variation in genes explain little of the variance in the phenotype, the biological pathway through which they operate could matter if intervened upon. Notable examples are hypercholesteremia and schizophrenia, where drugs that target specific genes have large therapeutic benefits, while the common genetic variants that lie in these genes only have small effect sizes in GWAS[71,72]. Dietary intake might be affected pharmacologically by drugs that target *FGF21*, which are currently on trial for diabetes and obesity[140].

9 Out-of-sample prediction

In this section, we assess the out-of-sample predictive power of polygenic scores (PGS) for the four diet composition phenotypes. We use European-ancestry individuals, as based on both self-reported and PC-based identification, in two of our cohorts as holdout samples: The Health and Retirement Study (HRS, $N = 2{,}344$) and the Rotterdam Study I (RSI, $N = 3{,}585$). While HRS and RSI were included in the full meta-analyses, we perform two additional meta-analyses which excluded HRS and RSI respectively, which is necessary to prevent overfitting. Here, we first describe the methods used to generate the polygenic scores and elaborate on how we measure prediction accuracy. Next, we report the predictive power for each phenotype and prediction cohort.

9.1 Method

A polygenic score for an individual, denoted $\widehat{PGS_i}$, can be constructed as a weighted sum of his or her genotypes at J loci:

$$\widehat{PGS_i} = \sum_{j=1}^{J} w_j g_{ij},$$

where w_j is the weight applied to SNP j and g_{ij} is the genotype of individual i at SNP j. Methodologies for PGS construction are determined by (i) how the weights w_j are generated, and (ii) which J loci are included in the calculation.

In order to generate the weights w_j, we used LDpred estimation, a Bayesian approach that weights each SNP by its conditional effect, given those of other SNPs[141]. In general, LDpred has been found to perform better than other, "cruder" approaches (e.g., pruning and thresholding)[141]. We performed LDpred estimation with a Gaussian prior for the distribution of effect sizes set to 1. We used cohort-specific genotype data as the reference sample for LD estimation after excluding related individuals and ancestry outliers.

We construct the scores based on only those SNPs in the HapMap consortium phase 3 release[142] that meet certain quality-control thresholds. For HRS, we only retain those variants with a call rate greater than 98% and without any problematic features from a known list[e]. For RSI, we filter out the SNPs with imputation quality (INFO) < 0.7, MAF < 0.05, and HWE P value < 0.001. Applying these thresholds to genotypic data of our prediction cohorts yields 1,144,445 and 1,137,088 SNPs for HRS and RSI, respectively.

We calculate the LD-adjusted weights with the LDpred software in our HRC reference panel (**Supplementary Information 3.3.1**) and obtain the final scores with PLINK by multiplying the genotype at each SNP by the corresponding LDpred-calculated weights and then summing over all included SNPs.

9.2 Prediction model and prediction accuracy

Our prediction analyses consist of running two ordinary least squares (OLS) regressions for each phenotype. First, we regress a phenotype on the following set of baseline covariates: sex, year of birth, year-of-birth squared, sex × year of birth and sex × year-of-birth squared, and the first 10 principal components (PCs) of the cohort-specific genotype matrix. Next, we run the same regression but with the polygenic scores included as one of the independent variables. Our measure of interest that captures the prediction accuracy is the incremental adjusted R^2, defined as the difference between the adjusted R^2 of these two regressions. We bootstrapped 95% confidence intervals for the incremental adjusted R^2 with the bootstrap percentile method and 1,000 iterations. Since the incremental adjusted R^2 can be negative, its confidence interval may also have a negative lower bound.

9.3 Results

Our prediction results are summarized in **Supplementary Table 9.1** and **Extended Figure 7**. The table reports some summary statistics for the regression sample, the estimated incremental adjusted R^2, and P value of the estimated coefficient on the score for each phenotype and

---

[e] This list can be downloaded from http://hrsonline.isr.umich.edu/sitedocs/genetics/candidategene/HRS1-2_dbGaPUserInfo_v3.pdf.

prediction cohort. Overall our polygenic scores explain 0.08% to 0.71% of the variation in dietary composition.

FAT. The incremental adjusted R^2 of the scores is 0.27% and 0.20% for the HRS and RSI cohorts, respectively. The estimated regression coefficients for the scores are distinguishable from zero ($P < 0.01$) for both cohorts.

PROTEIN. In the HRS, the incremental adjusted R^2 of the scores is 0.08%. In the RSI, we estimate a relatively greater predictive power, with incremental R^2 of 0.31%. While the estimated regression coefficient on the scores is not distinguishable from zero for HRS ($P = 0.088$), it is for RSI ($P = 0.0005$).

SUGAR. In the HRS, the incremental adjusted R^2 of the scores is 0.64%. In the RSI, we estimate an incremental R^2 of 0.62%. The estimated regression coefficients for both scores are highly distinguishable from zero ($P < 0.0005$).

CARBOHYDRATE. In the HRS, the incremental adjusted R^2 of the polygenic scores is 0.71%. In the RSI, we estimated a relatively smaller predictive power, with incremental R^2 of 0.38%. The estimated regression coefficients for both scores are distinguishable from zero ($P < 0.0005$).

9.4 Discussion

Overall, the difference in predictive power between phenotypes follows the difference in SNP-based heritability between phenotypes. That is, PROTEIN has the lowest estimates of SNP-based heritability, followed by FAT, CARBOHYDRATE and SUGAR. These analyses indicate that PROTEIN needs a larger sample size for genetic discovery to reach the same out-of-sample predictive power as the other macronutrients. For all macronutrients, larger GWAS discovery samples are needed for the polygenic scores to reach the the SNP-based heritability (which is the upper bound of variance that could be explained by a polygenic score).

Furthermore, we note that the predictive accuracy of the polygenic scores might be attenuated by the discrepancy of methods between the meta-analysis cohorts and holdout cohorts. That is, our meta-analyses consisted mainly of the UKB, which used a 24HDR method, while the holdout cohorts HRS and RS both used FFQs (**Supplementary Table 1.2**). This discrepancy in methods could downwardly bias genetic correlations between the meta-analysis and holdout cohorts, which would attenuate the predictive accuracy of the polygenic score[143]. Another source of imperfect genetic correlation between meta-analysis and holdout cohorts could be driven by cultural differences in eating habits. This could happen if cultural differences interact with genetic background, such that genetic factors have culture-specific influences on eating habits. However, we do note that the confidence intervals for the explained variance largely overlap between HRS and RS (see **Extended Data Figure 8** and **Supplementary Table 9.1**). Since the differences in point estimates may not be meaningful, we refrain from further speculation.

10 Genetic correlations with health and behavior

In this section, we estimate the extent of genetic overlap between our main phenotypes and a total of 33 relevant (mental) health and behavior measures. These span anthropometric, glycemic, cardiometabolic, lipid, socioeconomic, lifestyle, and neuropsychiatric domains. The rationale behind inclusion of each trait is summarized in **Supplementary Table 10.1.** We pre-registered a selection of 19 traits of interest in our publicly available analysis plan on Open Science Framework (July 2017). The five traits that were added later, and thus not pre-registered were: Alzheimer's disease (included because of our finding of an *APOE* association), physical activity level (included because it is clearly relevant for diet, and 23andMe provided this data as part of our collaboration), alcohol consumption (included because of our finding of an *ADH1B* association), Townsend Deprivation Index (informed by UKB data availability, and included because of the relationship between built environments and food intake[144]), and Anorexia Nervosa (informed by Psychiatric Genomic Consortium data availability, included because it is an eating disorder with both metabolic and behavioral causes that are relevant to dietary intake).

Later, we added an additional nine psychiatric traits, in order to further explore the genetic overlap between diet composition and psychiatric health (ADHD[145], alcohol dependence[146], autism spectrum disorder[147], bipolar disorder[148], depressive symptoms[63], depression[149], PGC cross-disorder analysis[150], schizophrenia[72], and subjective wellbeing[63]). The 23andMe physical activity findings were also updated in early 2019, as the GWAS sample size increased from N = 123,983 to N = 269,189.

Because we examine our four diet composition phenotypes and 33 other variables, we are at risk of finding spurious associations due to multiple hypothesis testing. To correct for this, we make a Bonferroni correction for 33 traits to the P value of each genetic correlation, and we only discuss results that are statistically significant after this correction. Since many of the phenotypes of interest are themselves genetically correlated, Bonferroni is over-conservative. While this ensures we are unlikely to find spurious results, it also means we may overlook some true correlations that do not meet Bonferroni significance.

The summary statistics for the majority of relevant health traits and behaviors are from LD hub[136], as indicated in **Supplementary Table 10.2**. The other traits are analyzed in accordance with the LD hub protocol to establish comparability (i.e., excluding the HLA region and any SNPs with $\chi^2 > 80$).

Physical activity level was measured in a sample of N = 269,189 participants from 23andMe (unpublished data). The 23andMe physical activity phenotype is based on a calculation of MET-minutes per week for various activities as a continuous trait, as described in the guidelines for data processing for the International Physical Activity Questionnaire (IPAQ[f]). The short form of the IPAQ was deployed on the 23andMe website to 23andMe research participants. Participants were given the option to skip any question, and skipped questions were treated as missing values

[f] https://sites.google.com/site/theipaq/

in calculating MET-minutes. MET-minutes were first log-transformed. Then, for each sex separately, we regressed out age, age-squared, age-cubed, the first ten ancestry principal components, and the platform used to genotype participants, and quantile-normalized the residuals. The results were combined across sexes, and the GWAS was ran on this phenotype, controlling for age, age-squared, age-cubed, sex, sex × age, sex × age-squared, sex × age-cubed, the first ten principal components, and the platform used to genotype participants

Townsend Deprivation Index and alcohol consumption (number of drinks weekly) are based on GWAS on the first release of the UKB, as described in Karlsson Linnér et al.[51]. Townsend Deprivation Index is a measure of the average socioeconomic status of the participant's housing area. It was calculated by the UKB on the basis of area unemployment rates, non-car ownership, non-house ownership, and overcrowding[151]. Higher scores indicate higher deprivation. Scores were assigned by UKB on the basis of the postal code of the participant's household address.

College completion[64] and height[152] are based on the most recent and publicly available GWAS summary statistics from 2016 and 2014, respectively, as LD hub only contained older summary statistics for these two traits.

10.1 Method

We estimate the genetic correlation, r_g, between two sets of summary statistics with the estimator

$$\hat{r}_g = \frac{\hat{\sigma}_g}{\sqrt{\hat{h}^2_{g_1}\hat{h}^2_{g_2}}}$$

where $\hat{\sigma}_g$ is the genetic covariance between traits g_1 and g_2 as estimated using bivariate LD Score regression[67,153], and $\hat{h}^2_{g_i}$ is the heritability of trait g_i as estimated using univariate LD Score regression for each trait individually.

Under a set of assumptions, bivariate LD Score regression produces unbiased estimates of genetic covariance, σ_g, using GWAS summary statistics for the two traits of interest and an LD Score reference panel. The LD Score reference panel captures the level of genetic variation that is tagged by a single SNP. We use LD Scores based on the European-ancestry individuals in the 1000 Genomes Project, which were computed by Finucane et al.[153]. We restrict our analysis to HapMap3 SNPs (with MAF > 0.01), which guarantees that all analyses are performed using a set of SNPs that are imputed with reasonable accuracy across all cohorts. We estimate standard errors using the LDSC software that uses a block jackknife over the SNPs.

10.2 Association with psychiatric traits

Our study is relevant to the newly emerging field of "nutritional psychiatry"[154,155], which takes a holistic approach to mental health. The central aim of nutritional psychiatry is to improve mental health in ways other than pharmacological intervention[155]. Specifically, it studies the effects of

nutrition on mental health, as mediated by nutritional effects on brain health, systemic inflammation, and the gut microbiome[156,157].

To provide empirical evidence relevant for nutritional psychiatry, we include additional genetic correlation analyses between the four macronutrients and nine psychiatric traits. These analyses take advantage of the fact that genetic correlations between macronutrient intake and mental health can now be estimated without the need to observe psychiatric diagnoses and macronutrient intake in the same samples[67], thus enabling new discoveries about relationships between diet and mental health.

In the literature, there is currently mixed evidence for the benefits of nutritional interventions in psychiatric illness. This may partly be caused by the fact that nutritional interventions are difficult to blind and subject to large placebo effects[158]. There is some evidence that fish oils (polyunsaturated omega-3 or 6 oils) have beneficial effects on depression[159], although recent experimental trials of more comprehensive dietary interventions have produced mixed results[158,160]. Associations between nutrition and psychiatric disorders outside of the mood spectrum, such as ADHD, remain controversial[161,162]. Overall, there are few studies that examine the effect of relative macronutrient intake on mental health[163]. One recent randomized trail found evidence that low-fat diets might have mental health benefits, while a review of small-scale trials found that psychosocial benefits are usually dependent on weight loss and independent of macronutrient composition[163]. Hence, the results from our exploratory genetic correlation analyses could be used to put forward novel hypotheses for relationships between relative macronutrient intake and psychiatric conditions.

10.3 Results

The results are displayed in **Supplementary Table 10.2** and **Figure 3.** We briefly summarize them here.

10.3.1 Genetic correlations with health

CARBOHYDRATE and SUGAR. Since the patterns of genetic correlations for CARBOHYDRATE and SUGAR are similar, we jointly summarize them here. We find significant negative genetic correlations between carbohydrate and sugar consumption and several measures of body fat composition: waist circumference (CARBOHYDRATE: r_g = -0.142, SE = 0.033, SUGAR: r_g = -0.132, SE = 0.033) and waist-to-hip ratio (CARBOHYDRATE: r_g = -0.175, SE = 0.037, SUGAR: r_g = -0.147, SE = 0.037). We also find a weak but significant negative genetic correlation between CARBOHYDRATE (though not SUGAR) and HDL ("good") cholesterol (r_g = -0.125, SE = 0.040).

PROTEIN. We find statistically highly significant and positive genetic correlations between PROTEIN and several measures of obesity including childhood obesity (r_g = 0.304, SE = 0.062), BMI (r_g = 0.402, SE = 0.041), overweight (i.e., BMI > 25, r_g = 0.383, SE = 0.045), obesity class I (i.e., BMI > 29, r_g = 0.352, SE = 0.040), waist circumference (r_g = 0.369, SE = 0.041) and waist-to-hip ratio (r_g = 0.279, SE = 0.039). These correlations with obesity are accompanied by positive correlations with several related diseases: type 2 diabetes (r_g = 0.445, SE = 0.062),

fasting insulin (r_g = 0.411, *SE* = 0.08) and coronary artery disease (r_g = 0.155, *SE* = 0.04). There is also a negative correlation with HDL ("good") cholesterol (r_g = -0.250, *SE* = 0.05).

FAT. After Bonferroni correction, we do not find any significant genetic correlations between FAT and health-related outcomes.

10.3.2 Genetic correlations with socioeconomic status and lifestyle

In this section we explore genetic correlations of dietary intake with socioeconomic, lifestyle and neuropsychiatric outcomes. We find several genetic correlations that are statistically significant after Bonferroni correction.

CARBOHYDRATE and SUGAR. Both CARBOHYDRATE and SUGAR have negative genetic correlations with the Townsend deprivation index (CARBOHYDRATE: r_g = -0.298, *SE* = 0.06, SUGAR: r_g = -0.231, *SE* = 0.064). This suggests that living in a lower socioeconomic region is associated with consuming relatively less energy from carbohydrates and sugars. We also find positive genetic correlations between physical activity and SUGAR (r_g = 0.225, *SE* = 0.042) and CARBOHYDRATE (r_g = 0.13, *SE* = 0.04). Thus, individuals who are more genetically prone to being physically active tend to have higher proportions of sugar intake in their diet. (CARBOHYDRATE's weak positive genetic correlation with physical activity is not statistically significant after Bonferroni correction.) Both CARBOHYDRATE and SUGAR have negative genetic correlations with alcohol consumption (r_g = -0.605, *SE* = 0.027 for CARBOHYDRATE, and r_g = -0.399, *SE* = 0.032 for SUGAR).

PROTEIN. The only SES or lifestyle outcome that correlates genetically with PROTEIN is alcohol consumption (r_g = -0.158, *SE* = 0.040).

FAT. FAT is the only macronutrient that has significant genetic correlations with educational attainment. Both college completion and years of education show a negative genetic correlation (r_g = -0.131, *SE* = 0.036 for college completion, and r_g = -0.105, *SE* = 0.033 for years of education), implying that individuals with a lower genetic prediction for education consume relatively more FAT. We also find that there is a significant negative genetic correlation between FAT and physical activity (r_g = -0.321, *SE* = 0.04). This stands in contrast with the positive genetic correlation found between SUGAR and physical activity.

The genetic correlation between FAT and alcohol consumption is borderline significant (*P* = 0.055) after Bonferroni correction (r_g = -0.112, *SE* = 0.037), which makes FAT the weakest and least correlated trait with alcohol consumption.

SATURATED FAT. Relative intake of saturated fat has significant genetic correlations with BMI (r_g = -0.115, *SE* = 0.035) and weekly alcohol consumption (r_g = -0.112, *SE* = 0.037). None of the other body fat indicators were significantly genetically correlated with SATURATED FAT, however, making this isolated finding hard to interpret. Alcohol dependence was also not genetically correlated with SATURATED FAT. This pattern stands in partial contrast with the genetic correlation pattern of total FAT, which did not have a significant genetic correlation with BMI (r_g = -0.017, *SE* = 0.047). SATURATED FAT did not have genetic correlations with educational attainment that were statistically distinguishable from zero (r_g = -0.10, *SE* = 0.04 for EduYears),

although they were similar in magnitude to FAT's genetic correlations with educational attainment r_g = -0.13, *SE* =0.04 for EduYears), which were statistically distinguishable from zero.

10.3.3 Correlations with neuropsychiatric outcomes

Between neuropsychiatric phenotypes and macronutrient intake, the only significant genetic correlations after Bonferroni correction are between ADHD and CARBOHYDRATE (r_g = -0.189, *SE* = 0.044) and between schizophrenia and SATURATED FAT (r_g = -0.133, *SE* = 0.037).

Despite *APOE* associations with FAT, SUGAR, and CARBOHYDRATE, there are no detectable (genome-wide) genetic correlations with Alzheimer's disease. CARBOHYDRATE and FAT show significant genetic correlations (positively, and negatively, respectively) with Anorexia Nervosa that lose significance after Bonferroni correction. Subjective wellbeing has a positive genetic correlation with SUGAR, (r_g = 0.135), but this is not significant after Bonferroni correction ($P_{Bonferroni}$ = 0.13). Finally, we note that none of the macronutrients had significant genetic correlations with alcohol dependence. This might indicate that the negative genetic correlations with weekly alcohol consumption described above are indicative of substitution effects.

10.4 Discussion

We find the largest and most widespread positive genetic correlations with poor health for PROTEIN. PROTEIN correlates genetically with all included obesity indicators (BMI, overweight, obesity, waist circumference, and waist-hip ratio) and childhood obesity, as well as related diseases and disease states (type 2 diabetes, fasting glucose and insulin, insulin resistance, coronary artery disease, and HDL cholesterol).

As with any correlation, it is impossible to infer a causal mechanism. Possible explanations might be that protein intake causally predisposes to obesity and metabolic syndrome, but other plausible explanations might be that obesity-prone individuals have a higher protein need, that overweight individuals use high-protein diets as a weight-loss strategy, or that the correlation is due to confounding by an unmeasured third variable (which may be environmental or genetic). The effect may also be mediated by a combination of the other diet composition phenotypes, as they are correlated with each other (**Supplementary Information 7**).

However, a causal role for protein (and especially meat) intake in the development of metabolic syndrome has been postulated[164–167]. If true, this causal effect of protein intake would contravene the view that protein intake should be increased due to its desirable effects of reducing hunger and increasing satiety[168–170]. It also contravenes a "Paleolithic" view of optimal dietary intake, which argues that energy intake from animal protein should be increased at the expense of carbohydrates, with the average Westerner consuming about 15% of energy from protein, compared to 19% for the average contemporary hunter-gatherer[157].

In terms of the socioeconomic and lifestyle indicators, we find that FAT genetically correlates with lower educational attainment (with small, negative genetic correlations with college completion and years of education, r_g = -0.131, *SE* = 0.036 for college completion). The genetic correlation with Townsend deprivation index was not significant after Bonferroni correction (r_g =

0.193, $SE = 0.066$). CARBOHYDRATE and SUGAR genetically correlate with indicators of higher socioeconomic status (where both have a negative correlation with Townsend deprivation index) but not with educational attainment ($r_g < 0.05$). More research is needed to clarify the reasons for these relationships.

We could speculate that the genetic overlap between diet composition and socioeconomic variables could be caused by horizontal (or "biological") pleiotropy, for instance through involvement of shared neural mechanisms that independently affect both educational attainment and diet composition. For instance, an underlying genetic factor for self-control could independently affect educational attainment and dietary habits. However, it is also possible that educational attainment affects dietary choices (i.e., vertical pleiotropy). The genetic correlations between CARBOHYDRATE/SUGAR and neighborhood deprivation could also be caused by environmental channels, such that individuals who live in deprived neighborhoods have restricted access to fresh high-carbohydrate foods such as bread and fruit/vegetables[171] (another example of vertical pleiotropy). Of course, the genetic overlap we found could also be spurious, caused by a third, unmeasured variable that confounds the relationship. These three scenarios (horizontal pleiotropy, vertical pleiotropy, and confounding) have different causal biological interpretations. In the phenotypic correlation analyses reported in **Supplementary Information 11.2.2**, we find that FAT has a small but consistent association with lower socioeconomic status, while CARBOHYDRATE surprisingly is associated with less neighborhood deprivation. Future research with the necessary data could explore these associations further, for instance with models that assess the mediating effect of self-control, or with Mendelian Randomization designs that test the causal influence of education and neighborhood deprivation on diet composition.

The positive genetic correlations between SUGAR and CARBOHYDRATE with physical activity, and the negative genetic correlation between FAT and physical activity, could explain some of the other patterns we observe. For instance, it is possible that the "favorable" genetic correlations between SUGAR and body fat composition (and the lack of genetic correlations with obesity and related diseases) are explained by the association between physical activity level and SUGAR. We explore this explanation in the phenotypic analyses in the next section (**Supplementary Information 11**), where we adjust the phenotypic associations between the macronutrients and BMI for physical activity in the UKB. There, we find that the phenotypic associations between relative sugar and carbohydrate intake become smaller but remain significant.

In the neuropsychiatric domain, we find two significant genetic correlations (ADHD and CARBOHYDRATE, $r_g = -0.189$; schizophrenia and SATURATED FAT, $r_g = -0.133$). One randomized-controlled study found that a restricted "few foods" diet significantly improves ADHD symptoms in young children with ADHD[162], although this diet does not focus on carbohydrates, but on foods that trigger allergic reactions. The relationship may also be confounded by socioeconomic status, as CARBOHYDRATE also show a significant negative genetic correlation with neighborhood deprivation. The genetic correlation between saturated fat and schizophrenia is in the opposite direction of what could be expected, since the diets of schizophrenia patients are typically of poorer quality, with higher consumption of saturated fats[172].

Finally, we note that the genetic correlations should be interpreted with the heritability estimates of diet composition in mind. That is, genetic covariance between traits with low heritability may only explain a very small proportion of the total covariance of two traits.

10.4.1 Comparison to genetic correlations from Merino et al.

In their GWAS of relative intake of carbohydrate, fat, and protein (defined as percentage of total energy intake), Merino et al. (N = 123,659) also assessed genetic correlations with various physical and mental health phenotypes. Specifically, they assessed genetic correlations with BMI; type 2 diabetes; coronary artery disease; various circulating fatty acids; fasting insulin and glucose; inflammatory bowel syndrome; years of education; and the psychiatric disorders schizophrenia, depression, anorexia nervosa, and bipolar disorder. In their Supplementary Figure S6 they report their results. We compare our findings in **Supplementary Table 10.4**. P values and standard errors were not reported by Merino et al., but effect estimates were printed in bold if they surpassed their Bonferroni-corrected significance threshold for 51 tests. We can roughly estimate their standard errors by multiplying our standard errors by the ratio of maximum sample sizes: $SE_{Merino} = SE_{SSGAC} \times \sqrt{264{,}181/123{,}659} = SE_{SSGAC} \times \sqrt{2.14}$. We use these estimated standard errors to calculate 95% confidence intervals, and report whether our confidence intervals overlap with those of Merino et al.

After a conservative Bonferroni correction for 51 tests, the only significant genetic correlations reported by Merino et al. are between BMI and protein (r_g = 0.23, compared to our r_g = 0.40), and fat and years of education (r_g = -0.24, compared to our r_g = -0.11). Other genetic correlations with protein intake are in the same direction, but differ in magnitude, with ours being larger in magnitude compared to those found by Merino et al. However, the confidence intervals of all reported genetic correlations, with one exception (relative carbohydrate intake and depression), overlap. Hence, we conclude that our genetic correlation estimates do not statistically differ from those reported by Merino et al.

11 Phenotypic associations between diet composition and BMI, SES, and alcohol abstinence

We examine the phenotypic associations between relative macronutrient intake and BMI in four large, independent cohorts from the UK and US (UKB, HRS, FHS, and WHI, with combined N = 173,165; **Supplementary Table 11.1**). In the HRS, FHS, and WHI, we are also able to distinguish animal vs. plant protein, natural vs. added sugars (only available in a subset for FHS), and saturated vs. unsaturated fat. In the UKB, only the distinction between saturated vs. unsaturated fat is available.

We focus on BMI, as it is readily available in all cohorts, and it is the most important proxy for metabolic syndrome, which is characterized by central obesity, high blood pressure, dyslipidemia, glycemia and insulin resistance, and consequentially cardiovascular disease and type 2 diabetes. Since the validity of BMI as a measure of adiposity is sometimes questioned[173],

we also report the associations between BMI and DEXA-scan measures of body fat in UKB, which is considered the "gold standard" measurement of adiposity.

Finally, we report associations between diet composition and indicators of socioeconomic status: completed years of education, household income, and the Townsend deprivation index.

11.1 Method

We estimate the standardized regression coefficients obtained from a 1) a simple regression of BMI on the focal macronutrient, and 2) a multiple regression of BMI on the focal macronutrient, sex, age, educational attainment, household income (available for all cohorts except FHS), and the number of measurements (for FHS, UKB, and WHI). In the UKB, we perform an additional regression that included a measure of overall physical activity. To ensure comparability, we restrict the simple regression in (1) to the set of individuals who had information on all covariates included in (2), and we only include individuals that were also included in the GWAS.

We always utilize the best available measures of dietary intake and BMI, which ideally would be balanced (i.e., measured at the same time). If several simultaneous BMI and dietary intake measurements were available (i.e., in FHS and WHI), we use their averages. In the HRS, BMI (last measured in 2010) was not measured during the dietary intake measurement (only measured in 2013), so we take the most recent available BMI report from 2010. In the UKB, a similar situation arises, as the additional online dietary intake questionnaires took place after the BMI measurement at baseline in the assessment center. Despite these differences in measurement, the pattern and magnitude of associations are strikingly similar across cohorts.

Making use of Fisher's Z-transformation, we performed fixed-effects, inverse-variance weighted meta-analysis of the standardized regression coefficients. Fisher's Z-transformation is also used to obtain 95% confidence intervals. **Figure 4** summarizes the estimates from the adjusted model in forest plots.

11.1.1 Framingham Heart Study

In FHS, we average the dietary intakes across several measurement waves (exam 20-22 for the original cohort, exam 5-8 for the offspring cohort, and exam 1-2 for the generation 3 cohort), and perform the log-log regressions with these average intakes. The measure of BMI used in this analysis is the average BMI across the same measurement waves. For the adjusted model, we use the following covariates: educational attainment (years of education), which was taken from the Neuropsychological Battery; the total number of dietary intake measurements; and sex and birth year.

11.1.2 Health and Retirement Study

One dietary intake measurement was available in HRS, which was part of the 2013 Health Care and Nutrition Study (HCNS). We use the most recent available measures of BMI and household income (wave 10, 2010). Educational attainment is measured by total years of schooling.

11.1.3 UK Biobank

In UKB, we average the total and macronutrient intakes across the five measurement waves (one at the assessment center and four additional online measurements), corrected for the day of the week the intake was reported (**Supplementary Information 2.5.2**). The assessment center measurements took place in 2009 and 2010, and the online measurements took place in 2011 and 2012. BMI was measured at the assessment center. Educational attainment is measured as total years of schooling. Household income was self-reported in four categories (coded as dummies in the regression) at the assessment center visit. For the adjusted model, we use the following covariates: educational attainment (years of education); household income; Townsend deprivation index; the total number of dietary intake measurements; and sex and birth year.

In addition to corrections for the demographic and socioeconomic variables described above, we estimate a third model in the UKB that includes covariates for physical activity (**Supplementary Table 11.1**) and self-reported walking pace. The physical activity measure is available for virtually all participants with dietary data in the UKB, as it was measured with three questions that were appended to the dietary intake questionnaire. Specifically, participants were asked how many minutes of light, moderate, and vigorous physical activity they performed the day before. We convert these answers into metabolic units in accordance with Anderson et al.[174] and then summed and log-transformed.

11.1.4 Women's Health Initiative

For WHI, we average the total macronutrient and energy intakes across years 0 through 10, and we used these average intakes in the phenotype construction. The BMI measure we use is the average BMI across years 0 through 10. For educational attainment, we use dummies for the "highest grade finished in school" variable. For household income, we use the most recent value of family income available (from year 6, year 3, or baseline, where the variables of year 3 and baseline were re-mapped to the year 6 categories). Additional covariates in the adjusted model are birth year, the number of dietary intake measurements, the number of BMI/dietary intake measurements, and dummies for the year from which family income was derived.

11.1.5 Associations between BMI and objective measures of adiposity in the UKB

In these analyses we focus on BMI, which is measured in almost all population cohorts and is related to a myriad of health outcomes. BMI is designed to be a measure of weight that is unrelated to height in order to derive an estimate of adiposity (the component of the variance in weight that is unrelated to body height and must therefore be due to variation in body mass).

As we reported earlier in **Supplementary Information 2.6**, we find that height squared is the correct empirical adjustment factor for height in the UKB, as a log-log regression of weight on height yielded a coefficient of $\hat{\beta} = 1.9995$. However, BMI is not a perfect measure of adiposity: while it has a high level of specificity (0.90), it has a relatively a low level of sensitivity (0.50)[173]. This means that some individuals at the extreme right tail of the distribution are misclassified as "obese" when they actually carry a relatively large amount of lean body mass. The opposite occurs far less often, with individuals at the extreme left tail of the distribution almost always being correctly identified as non-obese. However, BMI is used ubiquitously

because it is easily measured and has clearly defined clinical thresholds (e.g., underweight, morbidly obese) that are robustly associated with mortality and cardiometabolic outcomes[173]. By contrast, the gold standard for measuring body composition, dual energy x ray absorptiometry (DEXA), is a lengthy and extremely costly procedure, and its measures of body fat have not been translated to clinical thresholds[173].

For a subset of individuals (N = 5,107 European ancestry individuals that were included in our GWAS), UKB performed DEXA scans. BMI and impedance measures of body fat were available for almost the entire cohort. Below, we compare BMI, DEXA measures of body fat, and impedance measures of body fat.

11.2 Results

11.2.1 BMI

The results are reported in **Supplementary Table 10.2** and **Figure 4**. Here, we briefly summarize the range of findings for the regression coefficients from the adjusted model. In all cohorts, we find that all regression coefficients (for each macronutrient, both in the full and adjusted models) are statistically significant at $P < 0.001$ and always positive for BMI-FAT (range: $\hat{\beta}_{adj}$ = [0.051 (UKB), 0.167 (FHS)]) and BMI-PROTEIN (range: $\widehat{\beta}_{adj}$ = [0.082 (UKB), 0.161 (FHS)]) and always negative for BMI-SUGAR (range: $\hat{\beta}_{adj}$ = [-0.111 (FHS), -0.054 (WHI)]) and BMI-CARBOHYDRATE (range: $\hat{\beta}_{adj}$= [-0.144 (FHS), -0.068 (WHI)]). In the UKB, the associations between BMI and CARBOHYDRATE and SUGAR become slightly smaller in magnitude after a covariate for physical activity is added, while the associations between BMI and FAT and PROTEIN remain virtually unchanged.

In FHS, HRS and WHI, we are able to distinguish between animal protein versus plant protein; added versus natural sugar; and saturated versus unsaturated fat (**Supplementary Table 11.2, Extended Data Figure 9)**. The latter distinction is also available in the UKB. We find that animal protein has a consistent positive correlation with BMI, with range $\hat{\beta}_{adj}$ = [0.144 (HRS), 0.177 (FHS)]). Plant protein has a consistent negative correlation with BMI, with range $\hat{\beta}_{adj}$ = [-0.069 (FHS), -0.059 (HRS)]). The coefficients for saturated and unsaturated fat and natural versus added sugar, however, are always in the same direction, with meta-analyzed point estimates $\hat{\beta}_{adj}$ = 0.071 for saturated fat, $\hat{\beta}_{adj}$ = 0.028 for unsaturated fat, $\hat{\beta}_{adj}$ = -0.035 for added sugar, and $\hat{\beta}_{adj}$ = -0.058 for natural sugar (all $P < 0.001$).

These results show that higher relative intake of sugar and carbohydrate is consistently associated with lower BMI, while the opposite holds for relative protein and fat intake. For PROTEIN, the PROTEIN-BMI association is clearly driven by protein of animal origin.

For PROTEIN, the estimated coefficient of the phenotypic association with BMI is larger in the adjusted model in all four cohorts. Hence, SES appears to partially mask the association between relative protein intake and BMI, as PROTEIN is positively correlated with household income and negatively correlated with neighborhood deprivation in the UKB (but also negatively correlated with educational attainment; **Supplementary Table 11.4**). Adding covariate for physical activity

also and walking pace does not change the association between PROTEIN and BMI in the UKB, and the genetic correlation between PROTEIN and physical activity was also positive (yet non-significant). Hence, it appears that the positive phenotypic association between PROTEIN and BMI is not likely the result of confounding by SES or physical activity.

In the UKB, we also find that BMI is a good proxy measure for total fat mass, especially in women, with a higher explained variance in BMI by DEXA-estimate for total fat mass for women compared to men ($R^2 = 0.72$ for women vs. $R^2 = 0.66$ for men **Supplementary Table 11.3**). BMI is less accurate in detecting body fat percentage and visceral fat mass, however (explained variances for body fat percentage: $R^2 = 0.53$ for women and $R^2 = 0.47$ for men; visceral fat mass: $R^2 = 0.49$ for women and $R^2 = 0.53$ for men).

Lastly, we note that BMI does not necessarily have ubiquitous negative health effects across the lifespan, as body fat may protect against frailty in elderly populations. This phenomenon is known as "the obesity paradox"[175,176]. Indeed, Levine et al.[177] found that the negative metabolic health effects of high protein consumption reverse with older age. Hence, it would be premature to conclude that (animal) protein consumption leads to poor health based on our findings – not only because our findings are correlational but also because of potential age-dependent effects.

11.2.2 SES

In **Supplementary Table 11.4,** we report results of multiple regressions of the four macronutrients on the SES indicators: completed years of education, household income, and Townsend deprivation index in the UKB. In these regressions, the SES indicators are added simultaneously, together with covariates for sex, age, and home location coordinates. In general, the associations between the macronutrients and SES were small but significant (largest standardized $\hat{\beta} = -0.103$, for CARBOHYDRATE and household income). FAT is the only macronutrient that shows a consistent pattern of negative correlations with SES (i.e., negative correlation with education, positive correlation with deprivation, and negative correlation with income).

11.2.3 Current drinkers vs. current non-drinkers

In **Supplementary Information 5.2,** we speculated that the association between *ADH1B* and fat might be driven by a cross-sectional fat-alcohol substitution effect, where current drinkers consume alcohol at the expense of fat compared to non-drinkers. In a sensitivity analysis including only non-drinkers, we found that the association between *ADH1B* and fat disappeared (**Supplementary Information 5.2**), which provided suggestive support for this scenario. In this analysis, we used individuals who consumed 0 kilocalories of alcohol according to their dietary report. Here, we also explore the possibility that the diets of *former* drinkers contain a different macronutrient composition compared to current or never drinkers. For instance, former drinkers may change their dietary composition to compensate for the health effects of past alcohol use, or they could attempt to substitute one addictive dietary substance (alcohol) for another (fat).

To assess these scenarios, we compare the diet composition of current drinkers and current non-drinkers. We identify these groups on the basis of dietary self-report from the 24HDR data. These self-reports of alcohol intake used here are not corrected for the weekday the self-report

was for, as we did in **Supplementary Information 2.5.2** for the other macronutrients, as this could classify non-drinkers as drinkers if their reports were for weekend days. Individuals who reported consuming more than 0 kilocalories from alcohol in their 24HDR (dietary report) were classified as current drinkers. Individuals who report being former or never drinkers in the lifestyle and environment questionnaire (data-field 1558), but who did report consuming more than 20 kilocalories from alcohol, were removed from the analyses (*N* = 264). Individuals who identified as current drinkers according to data-field 1558, but who consumed 0 kilocalories from alcohol according to their 24HDR report, were also removed (*N* = 48,761), as were the remainder of individuals who did not have 24HDR data at all (*N* = 260,722). We remove these individuals from these analyses because we are interested in comparing former drinkers to the same set of individuals that were implicitly classified as "current" drinkers in our GWAS estimation of *ADH1B*'s effects. After excluding individuals, we are left with *N* = 117,101 confirmed current drinkers and *N* = 4,781 former drinkers. The current drinkers consumed an average of 180 (*SD* = 156) kilocalories from alcohol, which corresponds to 8.6% (*SD* = 7.0) percent of total energy intake for these individuals.

Because the effect size of the macronutrient density difference is easy to interpret, we use macronutrient densities in only these analyses. We address the correlation between macronutrient densities and total energy intake (**Supplementary Information 2.7**) by performing linear regressions that include a covariate for total energy intake. Since current drinkers might differ from non-drinkers with regards to demographic or lifestyle characteristics, we also add covariates for sex, birth year, educational attainment, household income, the number of dietary self-reports, height, weight, Townsend deprivation index, self-reported total physical activity (as defined in **Supplementary Information 11.1.3**), and walking pace.

We analyze percentage intake of macronutrients from total and non-alcoholic energy intake. We perform multiple regressions of the macronutrients (expressed in % intake from non-alcoholic energy) on a binary indicator for former drinking (1 for former drinkers, 0 for "confirmed" current drinkers) and the covariates described above.

The results are summarized in **Supplementary Table 11.5**. Comparing current and former drinkers, we find that former drinkers "swap" calories from alcohol for all other macronutrients, but especially for carbohydrates (+ 6.5%, *SE* = 0.1%) and sugar (+ 3.8%, *SE* = 0.1%), and less so for fat (+ 1.9%, *SE* = 0.1%) and protein (+ 0.4%, *SE* = 0.05%). These cross-sectional (i.e., inter-individual and not within-individual) substitution effects are estimated by including calories from alcohol in the total energy equation. However, when we exclude calories from alcohol from the total energy equation to make their non-alcoholic energy intakes comparable, we find that former drinkers consume relatively *less* fat (-1.5%, *SE* = 0.1%) and protein (-0.8%, *SE* = 0.1%) than current drinkers, and more carbohydrates (+ 2.3%, *SE* = 0.1%) and sugar (+1.9%, *SE* = 0.1%). Hence, it appears that former drinkers actually consume less fat and more carbohydrates than current drinkers when we focus on non-alcoholic calories.

We conclude that there are tangible differences in diet composition between former drinkers and current drinkers. However, it seems unlikely that an increased "health consciousness" of former drinkers drove the *ADH1B* association with FAT. In this (admittedly somewhat complex)

scenario, a subset of individuals with a genetic liability towards alcohol abuse (e.g., because of a genetic tendency towards fast alcohol metabolism[146]) consume relatively less fat after recovery from alcohol abuse. Since the GWAS phenotype we create is based on total energy estimates that include calories from alcohol, we actually find a discordant effect with respect to alcohol and fat intake. That is, the effect allele is associated with decreased alcohol intake is associated with *increased* fat intake. We also find this in our phenotypic association described above: being a former drinker is associated with a *higher* dietary fat percentage when alcohol is included in the total energy equation. When we remove alcohol from the total energy equation we do find that being a former drinker is associated with a lower dietary fat percentage, but the GWAS phenotype was agnostic to this effect.

We also do not find a significant overall genetic correlation with alcohol abuse and fat intake in **Supplementary Information 10.3.3**. Hence, a more straightforward explanation for the *ADH1B* association remains cross-sectional substitution of alcohol with fat, as a consequence of our phenotype construction. This scenario is supported by the fact that we do not find significant genetic correlations between diet composition and alcohol dependence. The only question that remains is why the *ADH1B* was only found for fat, when CARBOHYDRATE and SUGAR have much stronger phenotypic "substitution" effects for alcohol cross-sectionally.

12 Contributions

12.1 Author contributions

Carson Chow, Daniel Benjamin, Philipp Koellinger, and Fleur Meddens designed and oversaw the study. Ronald de Vlaming proposed the phenotype construction.

Fleur Meddens was the lead analyst, responsible for GWAS, quality control, meta-analysis, summarizing the overlap across the results of the various GWAS, heritability analyses, genetic correlation analysis, phenotypic association analyses, out-of-sample prediction, and all bioinformatics analyses. Richard Karlsson Linnér assisted with GWAS in UKB, and Richard Karlsson Linnér and Aysu Okbay assisted with cohort-level quality control.

Sean Lee performed the replication analyses and was supervised by Patrick Turley. Casper Burik performed the impG imputation of DietGen summary statistics.

Fleur Meddens prepared the majority of figures with assistance from Richard Karlsson Linnér; Peter Bowers and Sean Lee also prepared some figures. James Lee provided helpful advice and feedback on various aspects of the study design. All authors contributed to and critically reviewed the manuscript. Carson Chow, Daniel Benjamin, Philipp Koellinger, and Fleur Meddens made especially major contributions to the writing and editing.

12.2 Cohort contributions

Cohort	Name	Study design & Mgmt.	Data collection	Geno-typing	Genotype preparation	Phenotype preparation	Data analysis
ALSPAC	Emma L Anderson					x	
ALSPAC	George Davey Smith	x					
ALSPAC	Pauline M Emmett	x				x	
ALSPAC	George McMahon				x		x
ALSPAC	Susan Ring	x	x	x	x		
ALSPAC	Kaitlin H Wade					x	x
Fenland and EPIC-InterAct	Nita G Forouhi	x	x				
Fenland and EPIC-InterAct	Fumiaki Imamura					x	x
Fenland and EPIC-InterAct	Claudia Langenberg	x	x	x	x		
Fenland and EPIC-InterAct	Jian'an Luan	x			x		x
Fenland and EPIC-InterAct	Nick J Wareham	x	x	x	x		
Framingham Heart Study	Philipp D Koellinger		x				
Framingham Heart Study	S Fleur W Meddens				x	x	x
Health and Retirement Study	Philipp D Koellinger		x				
Health and Retirement Study	S Fleur W Meddens					x	
Health and Retirement Study	C A Rietveld				x		x
Lifelines	Jana V van Vliet-Ostaptchouk	x				x	x
Lifelines	Harold Snieder		x				
Lifelines	Peter J van der Most						x
Lifelines	Bruce HR Wolffenbuttel		x				
Rotterdam Study I	Oscar H Franco	x	x				
Rotterdam Study I	S Fleur W Meddens					x	
Rotterdam Study I	Taulant Muka		x			x	
Rotterdam Study I	Trudy Voortman		x			x	x
Rotterdam Study II	Mohsen Ghanbari		x		x		x

Rotterdam Study II	Jessica C Kiefte-de Jong		x			x	x
Rotterdam Study II	S Fleur W Meddens					x	
Rotterdam Study II	Andre G Uitterlinden	x	x	x			
Rotterdam Study II	Frank van Rooij	x	x		x	x	x
Rotterdam Study III	Kim VE Braun					x	
Rotterdam Study III	M Arfan Ikram	x	x				
Rotterdam Study III	S Fleur W Meddens					x	
Rotterdam Study III	C A Rietveld				x		x
Rotterdam Study III	Fernando Rivadeneira	x	x	x			
UK Biobank	Richard Karlsson Linnér				x		
UK Biobank	Philipp D Koellinger		x				
UK Biobank	S Fleur W Meddens					x	x
Women's Health Initiative	Philipp D Koellinger		x				
Women's Health Initiative	S Fleur W Meddens				x	x	x

13 Additional acknowledgements

13.1 Author acknowledgements

Carson C Chow was supported by the Intramural Research Program of the NIH, NIDDK.

Pauline M Emmett acknowledges funding from Nestlé Nutrition.

Tõnu Esko is supported by the Estonian Research Council Grant IUT20-60 and PUT1660, EU H2020 grant 692145, and European Union through the European Regional Development Fund (Project No. 2014-2020.4.01.15-0012) GENTRANSMED.

Juan R Gonzalez is supported by Spanish Ministry of Economy and Competitiveness (MTM2015-68140-R).

Philipp D Koellinger acknowledges financial support from the European Research Council (consolidator grant 647648).

Fleur Meddens gratefully acknowledges funding from NORFACE-DIAL (Grant number: 462-16-100).

Niels Rietveld gratefully acknowledges funding from the Netherlands Organization for Scientific Research (NWO Veni grant 016.165.004).

Kaitlin Wade is supported by the Elizabeth Blackwell Institute for Health Research, University of Bristol and the Wellcome Trust Institutional Strategic Support Fund [204813/Z/16/Z].

Nick J Wareham and Nita G Forouhi acknowledge support from the National Institute for Health Research Cambridge Biomedical Research Centre [IS-BRC-1215-20014] and NJW is an NIHR Senior Investigator.

13.2 Extended acknowledgements

23andMe, Inc. – We gratefully acknowledge the contributions of members of 23andMe's Research Team, whose names are listed below:

Michelle Agee, Babak Alipanahi, Adam Auton, Robert K. Bell, Katarzyna Bryc, Sarah L. Elson, Pierre Fontanillas, Nicholas A. Furlotte, David A. Hinds, Karen E. Huber, Aaron Kleinman, Nadia K. Litterman, Jennifer C. McCreight, Matthew H. McIntyre, Joanna L. Mountain, Carrie A.M. Northover, Steven J. Pitts, J. Fah Sathirapongsasuti, Olga V. Sazonova, Janie F. Shelton, Suyash Shringarpure, Chao Tian, Joyce Y. Tung, Vladimir Vacic, and Catherine H. Wilson.

LifeLines – Behrooz Z Alizadeh, H Marike Boezen, Lude Franke, Pim van der Harst, Gerjan Navis, Marianne Rots, Harold Snieder, Morris Swertz, Bruce HR Wolffenbuttel, Cisca Wijmenga.

13.3 Cohort acknowledgments

23andMe, Inc. – We would like to thank the research participants and employees of 23andMe for making this work possible. 23andMe research participants provided informed consent and participated in the research online, under a protocol approved by the AAHRPP-accredited institutional review board, Ethical and Independent Review Services (E&I Review). Participant data are shared according to community standards that have been developed to protect against breaches of privacy. Currently, these standards allow for the sharing of summary statistics for at most 10,000 SNPs. The full set of summary statistics can be made available to qualified investigators who enter into an agreement with 23andMe that protects participant confidentiality. Interested investigators should email dataset-request@23andme.com for more information.

ALSPAC – The MRC IEU is supported by the Medical Research Council and the University of Bristol (grant code MC_UU_12013/1-9, MC_UU_00011/1-7). The authors are extremely grateful to all the families who took part in the ALSPAC study, the midwives for their help in recruiting them, and the whole ALSPAC team, which includes interviewers, computer and laboratory technicians, clerical workers, research scientists, volunteers, managers, receptionists, and nurses. The UK Medical Research Council and the Wellcome Trust (Grant ref: 102215/2/13/2) and the University of Bristol provide core support for ALSPAC. ALSPAC GWAS data was generated by Sample Logistics and Genotyping Facilities at the Wellcome Trust Sanger Institute and LabCorp (Laboratory Corporation of America) supported by 23andMe. ALSPAC summary data will be published on the data repository at www.data.bris.ac.uk. Please note that the study website contains details of all the data that is available through a fully searchable data dictionary (http://www.bristol.ac.uk/alspac/researchers/our-data/). Ethical approval for the study was obtained from the ALSPAC Ethics and Law Committee and the Local Research Ethics Committees.

EPIC-InterAct – The EPIC-InterAct Study is funded by EU-FP6 programme (LSHM_CT_2006_037197). Support is acknowledged from Medical Research Council Epidemiology Unit (MC_UU_12015/1, MC_UU_12015/). We thank N. Kerrison, S. Dawson, M. Sims, and all staff from the Technical, and Data Management Teams of the Medical Research Council (MRC) Epidemiology Unit, University of Cambridge, Cambridge, United Kingdom.

DietGen – DietGen thanks the participants and the staff of the Health Professionals' Follow-up Study (HPFS), Nurses' Health Study (NHS) and Women's Genome Health Study (WGHS) for their continued contributions. The HPFS and NHS were supported by grants DK091718, HL71981, HL073168, CA87969, CA49449, HL34594, HL088521, U01HG004399, DK080140, DK58845 and DK46200 from the National Institutes of Health. The WGHS is supported by HL043851, HL080467 and CA058988 from the National Institutes of Health with collaborative scientific support and funding for genotyping provided by Amgen. This manuscript was not prepared in collaboration with investigators of DietGen and does not necessarily reflect the opinions or views of the DietGen consortium, CHARGE consortium, Nurses' Health Study, Women's Genome Health Study, or Health Professionals' Follow-up Study.

Fenland – The Fenland Study (10.22025/2017.10.101.00001) is funded by the Medical Research Council (MC_UU_12015/1). We are grateful to all the volunteers and to the General Practitioners and practice staff for assistance with recruitment. We thank the Fenland Study Investigators, Fenland Study Co-ordination team and the Epidemiology Field, Data and Laboratory teams. We further acknowledge support for genomics from the Medical Research Council (MC_PC_13046).

Framingham Heart Study (FramHS) – The Framingham Heart Study is conducted and supported by the National Heart, Lung, and Blood Institute (NHLBI) in collaboration with Boston University (Contract No. N01-HC-25195 and HHSN268201500001I). Funding for SHARe Affymetrix genotyping was provided by NHLBI Contract N02-HL64278. SHARe Illumina genotyping was provided under an agreement between Illumina and Boston University. Funding for Affymetrix genotyping of the FHS Omni cohorts was provided by Intramural NHLBI funds from Andrew D. Johnson and Christopher J. O'Donnell. Funding support for the Framingham Food Frequency Questionnaire dataset was provided by ARS Contract #53- 3k06-5-10, ARS Agreement #'s 58-1950-9-001, 58-1950-4-401 and 58-1950-7-707. This manuscript was not prepared in collaboration with investigators of the Framingham Heart Study and does not necessarily reflect the opinions or views of the Framingham Heart Study, Boston University, or NHLBI. The datasets used for the analyses described in this manuscript were obtained from dbGaP at http://www.ncbi.nlm.nih.gov/sites/entrez?db=gap through dbGaP accession phs000007.v29.p10.

Health and Retirement Study (HRS) – HRS is supported by the National Institute on Aging (NIA U01AG009740). The genotyping was funded separately by the National Institute on Aging (RC2 AG036495, RC4 AG039029). Genotyping was conducted by the NIH Center for Inherited Disease Research (CIDR) at Johns Hopkins University. Genotyping quality control and final preparation of the data were performed by the Genetics Coordinating Center at the University of Washington. Genotype data can be accessed via the database of Genotypes and Phenotypes

(dbGaP, http://www.ncbi.nlm.nih.gov/gap, accession number phs000428.v1.p1). Researchers who wish to link genetic data with other HRS measures that are not in dbGaP, such as educational attainment, must apply for access from HRS. See the HRS website (http://hrsonline.isr.umich.edu/gwas) for details.

Lifelines – The Lifelines Cohort Study, and generation and management of GWAS genotype data for the Lifelines Cohort Study is supported by the Netherlands Organization of Scientific Research NWO (grant 175.010.2007.006), the Economic Structure Enhancing Fund (FES) of the Dutch government, the Ministry of Economic Affairs, the Ministry of Education, Culture and Science, the Ministry for Health, Welfare and Sports, the Northern Netherlands Collaboration of Provinces (SNN), the Province of Groningen, University Medical Center Groningen, the University of Groningen, Dutch Kidney Foundation and Dutch Diabetes Research Foundation. The authors wish to acknowledge the services of the Lifelines Cohort Study, the contributing research centers delivering data to Lifelines, and all the study participants.
Researchers can apply for data, samples or an add-on study by filling in the application form for research and submitting the completed form through our data catalogue, together with a selection of the requested data. Please contact Lifelines Research office by emailing to LLscience@umcg.nl or calling to +31 50 3615803; when you may need more specific information.

Rotterdam Study (RS) – The generation and management of GWAS genotype data for the Rotterdam Study is supported by the Netherlands Organisation of Scientific Research NWO Investments (nr. 175.010.2005.011, 911-03-012). This study is funded by the Research Institute for Diseases in the Elderly (014-93-015; RIDE2), the Netherlands Genomics Initiative (NGI)/Netherlands Organisation for Scientific Research (NWO) project nr. 050-060-810. We thank Pascal Arp, Mila Jhamai, Marijn Verkerk, Lizbeth Herrera and Marjolein Peters for their help in creating the GWAS database, Karol Estrada and Maksim V. Struchalin for their support in creation and analysis of imputed data, and Jeanne de Vries, Saskia Meyboom en Karin Borgonjen from the Department of Nutrition, Wageningen University for their assistance in dietary data processing of The Rotterdam Study. The Rotterdam Study is funded by Erasmus Medical Center and Erasmus University, Rotterdam, Netherlands Organization for the Health Research and Development (ZonMw), the Research Institute for Diseases in the Elderly (RIDE), the Ministry of Education, Culture and Science, the Ministry for Health, Welfare and Sports, the European Commission (DG XII), and the Municipality of Rotterdam. The authors are grateful to the study participants, the staff of the Rotterdam Study and the participating general practitioners and pharmacists.

UK Biobank (UKB) – This research has been conducted using the UK Biobank Resource. Informed consent was obtained from UK Biobank subjects.

Women's Health Initiative (WHI) – The WHI program is funded by the National Heart, Lung, and Blood Institute, National Institutes of Health, U.S. Department of Health and Human Services through contracts HHSN268201600018C, HHSN268201600001C, HHSN268201600002C, HHSN268201600003C, and HHSN268201600004C. Funding support for WHI GARNET was provided through the NHGRI Genomics and Randomized Trials Network (GARNET) (Grant Number U01 HG005152). Assistance with phenotype

harmonization and genotype cleaning, as well as with general study coordination, was provided by the GARNET Coordinating Center (U01 HG005157). Assistance with data cleaning was provided by the National Center for Biotechnology Information. Funding support for genotyping, which was performed at the Broad Institute of MIT and Harvard, was provided by the NIH Genes, Environment and Health Initiative [GEI] (U01 HG004424). The WHI Sight Exam and the Memory Study was funded in part by Wyeth Pharmaceuticals, Inc, St. Davids, PA.
This manuscript was not prepared in collaboration with investigators of the WHI, has not been reviewed and/or approved by the Women's Health Initiative (WHI), and does not necessarily reflect the opinions of the WHI investigators or the NHLBI. The datasets used for the analyses described in this manuscript were obtained from dbGaP at through dbGaP accession phs000200.v11.p3).

14 References

1. Hewitt, J., Walters, M., Padmanabhan, S. & Dawson, J. Cohort profile of the UK Biobank: diagnosis and characteristics of cerebrovascular disease. *BMJ Open* **6**, e009161 (2016).
2. Scholtens, S. *et al.* Cohort Profile: LifeLines, a three-generation cohort study and biobank. *Int. J. Epidemiol.* **44**, 1172–1180 (2015).
3. Ikram, M. A. *et al.* The Rotterdam Study: 2018 update on objectives, design and main results. *Eur. J. Epidemiol.* **32**, 807–850 (2017).
4. Boyd, A. *et al.* Cohort Profile: The ' Children of the 90s '— the index offspring of the Avon Longitudinal Study of Parents and Children. *Int. J. Epidemiol.* 111–127 (2013). doi:10.1093/ije/dys064
5. Fraser, A. *et al.* Cohort Profile: The Avon Longitudinal Study of Parents and Children: ALSPAC mothers cohort. *Int. J. Epidemiol.* **42**, 97–110 (2013).
6. Rolfe, E. D. L. *et al.* Association between birth weight and visceral fat in adults. *Am. J. Clin. Nutr.* **92**, 347–352 (2010).
7. Tsao, C. W. & Vasan, R. S. Cohort Profile: The Framingham Heart Study (FHS): overview of milestones in cardiovascular epidemiology. *Int. J. Epidemiol.* **44**, 1800–1813 (2015).
8. Sonnega, A. *et al.* Cohort Profile: the Health and Retirement Study (HRS). *Int. J. Epidemiol.* **43**, 576–85 (2014).
9. The Women's Health Initiative Study Group. Design of the Women's Health Initiative Clinical Trial and Observational Study. *Control. Clin. Trials* **19**, 61–109 (1998).
10. InterAct Consortium *et al.* The InterAct Project: An Examination of the Interaction of Genetic and Lifestyle Factors on the Incidence of Type 2 Diabetes in the EPIC Study. *Diabetologia* **54**, 2272–2282 (2014).
11. Tanaka, T. *et al.* Genome-wide meta-analysis of observational studies shows common genetic variants associated with macronutrient intake. *Am. J. Clin. Nutr.* **97**, 1395–1402 (2013).
12. Chu, A. Y. *et al.* Novel locus including FGF21 is associated with dietary macronutrient intake. *Hum. Mol. Genet.* **22**, 1895–1902 (2013).
13. Pasaniuc, B. *et al.* Fast and accurate imputation of summary statistics enhances evidence of functional enrichment. *Bioinformatics* **30**, 2906–14 (2014).
14. Dhurandhar, N. V *et al.* Energy balance measurement: when something is not better than nothing. *Int. J. Obes.* **39**, 1109–1113 (2015).

15. Poslusna, K., Ruprich, J., De Vries, J. H. M., Jakubikova, M. & Van 't Veer, P. Misreporting of energy and micronutrient intake estimated by food records and 24 hour recalls, control and adjustment methods in practice. *Br. J. Nutr.* **101**, S73–S85 (2009).
16. Hall, K. D. & Guo, J. Obesity energetics: body weight regulation and the effects of diet composition. *Gastroenterology* **152**, 1718–1727 (2017).
17. Mifflin, M. D. *et al.* A new predictive equation for resting energy expenditure in healthy individuals. *Am. J. Clin. Nutr.* **51**, 241–247 (1990).
18. Willett, W. Nutritional epidemiology. in *Nutritional Epidemiology* (Oxford University Press, 2013). doi:10.1093/acprof:oso/9780199754038.001.0001
19. Slimani, N. *et al.* The standardized computerized 24-h dietary recall method EPIC-Soft adapted for pan-European dietary monitoring. *Eur. J. Clin. Nutr.* **65**, S5–S15 (2011).
20. Slimani, N. *et al.* The EPIC nutrient database project (ENDB): a first attempt to standardize nutrient databases across the 10 European countries participating in the EPIC study. *Eur. J. Clin. Nutr.* **61**, 1037–56 (2007).
21. Margetts, B. M. & Pietinen, P. European Prospective Investigation into Cancer and Nutrition: validity studies on dietary assessment methods. *Int. J. Epidemiol.* **26**, S1-5 (1997).
22. Rhee, J. J. *et al.* Comparison of methods to account for implausible reporting of energy intake in epidemiologic studies. *Am. J. Epidemiol.* **181**, 225–233 (2015).
23. Winkler. The fundamental flaw in obesity research. *Obes. Rev.* **6**, 199–202 (2005).
24. Willett, W. C., Howe, G. & Kushi, L. Adjustment for total energy. *Am. J. Clin. Nutr.* 120S-128S (1997).
25. Naska, A., Lagiou, A. & Lagiou, P. Dietary assessment methods in epidemiological research: current state of the art and future prospects. *F1000Research* **6**, 926 (2017).
26. Poppitt, S. D., Swann, D., Black, A. E. & Prentice, A. M. Assessment of selective under-reporting of food intake by both obese and non-obese women in a metabolic facility. *Int. J. Obes.* **22**, 303–311 (1998).
27. Robinson, E., Hardman, C. A., Halford, J. C. G. & Jones, A. Eating under observation: A systematic review and meta-analysis of the effect that heightened awareness of observation has on laboratory measured energy intake. *Am. J. Clin. Nutr.* **102**, 324–337 (2015).
28. O'Loughlin, G. *et al.* Using a wearable camera to increase the accuracy of dietary analysis. *Am. J. Prev. Med.* **44**, 297–301 (2013).
29. Heitmann, B. L. & Lissner, L. Dietary underreporting by obese individuals--is it specific or non-specific? *BMJ* **311**, 986–9 (1995).
30. Lissner, L., Heitmann, B. L. & Lindroos, A. K. Measuring intake in free-living human subjects: a question of bias. *Proc. Nutr. Soc.* **57**, 333–339 (1998).
31. Freedman, L. S. *et al.* Pooled results from 5 validation studies of dietary self-report instruments using recovery biomarkers for potassium and sodium intake. *Am. J. Epidemiol.* **181**, 473–487 (2015).
32. Heitmann, B. L. & Lissner, L. Dietary underreporting by obese individuals-is it specific or non-specific? *BMJ* **311**, 986–989 (1995).
33. Ankarfeldt, M. Z. *et al.* Dietary protein and urinary nitrogen in relation to 6-year changes in fat mass and fat-free mass. *Int. J. Obes.* **39**, 162–168 (2015).
34. Black, A. E. Critical evaluation of energy intake using the Goldberg cut-off for energy intake:basal metabolic rate. A practical guide to its calculation, use and limitations. *Int. J.*

Obes. **24**, 1119–1130 (2000).
35. Vainik, U. *et al.* Diet misreporting can be corrected: confirmation of the association between energy intake and fat-free mass in adolescents. *Br. J. Nutr.* **116**, 1425–1436 (2016).
36. Liu, B. *et al.* Development and evaluation of the Oxford WebQ, a low-cost, web-based method for assessment of previous 24 h dietary intakes in large-scale prospective studies. *Public Health Nutr.* **14**, (2011).
37. Galante, J. *et al.* The acceptability of repeat Internet-based hybrid diet assessment of previous 24-h dietary intake: administration of the Oxford WebQ in UK Biobank. *Br. J. Nutr.* **115**, 681–686 (2016).
38. Holland, B., Welch, A. & Unwin, I. *McCance and Widdowson's the Composition of Foods*. (Royal Society of Chemistry, 1991). doi:10.1039/9781849737562
39. Borys, J. *et al.* Does energy intake underreporting involve all kinds of food or only specific food items? Results from the Fleurbaix Laventie Ville Santé (FLVS) study. *Int. J. Obes.* **24**, 1500–1506 (2002).
40. Ballard-Barbash, R. *et al.* Low energy reporters vs others: a comparison of reported food intakes. *Eur. J. Clin. Nutr.* **54**, 281–287 (2002).
41. Cade, J. E., Burley, V. J., Warm, D. L., Thompson, R. L. & Margetts, B. M. Food-frequency questionnaires: a review of their design, validation and utilisation. *Nutr. Res. Rev.* **17**, 5–22 (2004).
42. Thompson, F. E. *et al.* Weekend-weekday differences in reported dietary intake: The nationwide food consumption survey, 1977–78. *Nutr. Res.* **6**, 647–662 (1986).
43. Yang, P. H. W., Black, J. L., Barr, S. I. & Vatanparast, H. Examining differences in nutrient intake and dietary quality on weekdays versus weekend days in Canada. *Appl. Physiol. Nutr. Metab.* **39**, 1413–1417 (2014).
44. Carroll, R. J. *et al.* Taking advantage of the strengths of 2 different dietary assessment instruments to improve intake estimates for nutritional epidemiology. *Am. J. Epidemiol.* **175**, 340–7 (2012).
45. Kronmal, R. A. Spurious correlation and the fallacy of the ratio standard revisited. *Journal of the Royal Statistical Society: Series A (Statistics in Society)* **156**, 379–392 (1993).
46. Freedman, L. S. *et al.* Evaluation of the 24-hour recall as a reference instrument for calibrating other self-report instruments in nutritional cohort studies: Evidence from the validation studies pooling project. *Am. J. Epidemiol.* **186**, 73–82 (2017).
47. Willett, W. C. Commentary: Dietary diaries versus food frequency questionnaires - A case of undigestible data. *Int. J. Epidemiol.* **30**, 317–319 (2001).
48. Talukdar, S. *et al.* FGF21 regulates sweet and alcohol preference. *Cell Metab.* **23**, 344–349 (2016).
49. Von Holstein-Rathlou, S. *et al.* FGF21 mediates endocrine control of simple sugar intake and sweet taste preference by the liver. *Cell Metab.* **23**, 335–343 (2016).
50. Laeger, T. *et al.* FGF21 is an endocrine signal of protein restriction. *J. Clin. Invest.* **124**, 3913–3922 (2014).
51. Karlsson Linnér, R. *et al.* Genome-wide association analyses of risk tolerance and risky behaviors in over 1 million individuals identify hundreds of loci and shared genetic influences. *Nat. Genet.* **51**, 245–257 (2019).
52. Genotyping and quality control of UK Biobank, a large-scale, extensively phenotyped prospective resource. 1–25 (2015). Available at:

https://biobank.ctsu.ox.ac.uk/crystal/docs/genotyping_qc.pdf. (Accessed: 2nd August 2016)
53. Loh, P.-R. *et al.* Efficient Bayesian mixed-model analysis increases association power in large cohorts. *Nat. Genet.* **47**, 284–290 (2015).
54. Bycroft, C. *et al.* Genome-wide genetic data on ~500,000 UK Biobank participants. *bioRxiv* https://doi.org/10.1101/166298 (2017). doi:10.1101/166298
55. Galinsky, K. J. *et al.* Fast principal-component analysis reveals convergent evolution of ADH1B in Europe and East Asia. *Am. J. Hum. Genet.* **98**, 456–472 (2016).
56. Yang, J., Lee, S. H., Goddard, M. E. & Visscher, P. M. GCTA: a tool for genome-wide complex trait analysis. *Am. J. Hum. Genet.* **88**, 76–82 (2011).
57. Yang, J., Zaitlen, N. A., Goddard, M. E., Visscher, P. M. & Price, A. L. Advantages and pitfalls in the application of mixed-model association methods. *Nat. Genet.* **46**, 100–6 (2014).
58. McCarthy, S. *et al.* A reference panel of 64,976 haplotypes for genotype imputation. *Nat. Genet.* **48**, 1279–1283 (2016).
59. Walter, K. *et al.* The UK10K project identifies rare variants in health and disease. *Nature* **526**, 82–90 (2015).
60. Chang, C. C. *et al.* Second-generation PLINK: rising to the challenge of larger and richer datasets. *Gigascience* **4**, 7 (2015).
61. Purcell, S. *et al.* PLINK: a tool set for whole-genome association and population-based linkage analyses. *Am. J. Hum. Genet.* **81**, 559–75 (2007).
62. Winkler, T. W. *et al.* Quality control and conduct of genome-wide association meta-analyses. *Nat. Protoc.* **9**, 1192–1212 (2014).
63. Okbay, A. *et al.* Genetic variants associated with subjective well-being, depressive symptoms, and neuroticism identified through genome-wide analyses. *Nat. Genet.* **48**, 624–633 (2016).
64. Okbay, A. *et al.* Genome-wide association study identifies 74 loci associated with educational attainment. *Nature* **533**, 539–542 (2016).
65. Willer, C. J., Li, Y. & Abecasis, G. R. METAL: fast and efficient meta-analysis of genomewide association scans. *Bioinformatics* **26**, 2190–1 (2010).
66. Rietveld, C. A. *et al.* GWAS of 126,559 individuals identifies genetic variants associated with educational attainment. *Science (80-.).* **340**, 1467–1471 (2013).
67. Bulik-Sullivan, B. K. *et al.* LD Score regression distinguishes confounding from polygenicity in genome-wide association studies. *Nat. Genet.* **47**, 291–295 (2015).
68. Turley, P. *et al.* Multi-trait analysis of genome-wide association summary statistics using MTAG. *Nat. Genet.* **50**, 229–237 (2018).
69. Merino, J. *et al.* Genome-wide meta-analysis of macronutrient intake of 91,114 European ancestry participants from the cohorts for heart and aging research in genomic epidemiology consortium. *Mol. Psychiatry* [Epub ahead of print] (2018). doi:10.1038/s41380-018-0079-4
70. Hamer, D. & Sirota, L. Beware the chopsticks gene. *Mol. Psychiatry* **5**, 11–13 (2000).
71. Price, A. L., Spencer, C. C. A. & Donnelly, P. Progress and promise in understanding the genetic basis of common diseases. *Proc. R. Soc. B Biol. Sci.* **282**, 20151684 (2015).
72. Schizophrenia Working Group of the Psychiatric Genomics Consortium, S. W. G. of the P. G. *et al.* Biological insights from 108 schizophrenia-associated genetic loci. *Nature* **511**, 421–7 (2014).

73. Dittmar, E. L., Oakley, C. G., Conner, J. K., Gould, B. A. & Schemske, D. W. Factors influencing the effect size distribution of adaptive substitutions. *Proc. R. Soc. B Biol. Sci.* **283**, (2016).
74. So, W. Y. & Leung, P. S. Fibroblast Growth Factor 21 as an emerging therapeutic target for type 2 diabetes mellitus. *Med. Res. Rev.* **36**, 705–748 (2016).
75. Hayes, M. G. *et al.* Identification of HKDC1 and BACE2 as genes influencing glycemic traits during pregnancy through genome-wide association studies. *Diabetes* **62**, 3282–91 (2013).
76. Saxena, R. *et al.* Genetic variation in GIPR influences the glucose and insulin responses to an oral glucose challenge. *Nat. Genet.* **42**, 142–8 (2010).
77. Johansen, C. T. *et al.* Excess of rare variants in genes identified by genome-wide association study of hypertriglyceridemia. *Nat. Genet.* **42**, 684–7 (2010).
78. Kathiresan, S. *et al.* Common variants at 30 loci contribute to polygenic dyslipidemia. *Nat. Genet.* **41**, 56–65 (2009).
79. Spracklen, C. N. *et al.* Association analyses of East Asian individuals and trans-ancestry analyses with European individuals reveal new loci associated with cholesterol and triglyceride levels. *Hum. Mol. Genet.* **26**, 1770–1784 (2017).
80. Surakka, I. *et al.* The impact of low-frequency and rare variants on lipid levels. *Nat. Genet.* **47**, 589–97 (2015).
81. Willer, C. J. *et al.* Discovery and refinement of loci associated with lipid levels. *Nat. Genet.* **45**, 1274–1283 (2013).
82. Weissglas-Volkov, D. *et al.* Genomic study in Mexicans identifies a new locus for triglycerides and refines European lipid loci. *J. Med. Genet.* **50**, 298–308 (2013).
83. Vaxillaire, M. *et al.* The common P446L polymorphism in GCKR inversely modulates fasting glucose and triglyceride levels and reduces type 2 diabetes risk in the DESIR prospective general French population. *Diabetes* **57**, 2253–2257 (2008).
84. Adams, A. C. & Gimeno, R. E. The sweetest thing: Regulation of macronutrient preference by FGF21. *Cell Metab.* **23**, 227–228 (2016).
85. Ogawa, Y. *et al.* betaKlotho is required for metabolic activity of fibroblast growth factor 21. *Proc. Natl. Acad. Sci.* **104**, 7432–7437 (2007).
86. Kurosu, H. *et al.* Tissue-specific expression of βklotho and Fibroblast Growth Factor (FGF) receptor isoforms determines metabolic activity of FGF19 and FGF21. *J. Biol. Chem.* **282**, 26687–26695 (2007).
87. Schumann, G. *et al.* KLB is associated with alcohol drinking, and its gene product β-Klotho is necessary for FGF21 regulation of alcohol preference. *Proc. Natl. Acad. Sci. U. S. A.* **113**, 14372–14377 (2016).
88. Lonsdale, J. *et al.* The Genotype-Tissue Expression (GTEx) project. *Nat. Genet.* **45**, 580–585 (2013).
89. Cornelis, M. C. *et al.* Genome-wide meta-analysis identifies regions on 7p21 (AHR) and 15q24 (CYP1A2) as determinants of habitual caffeine consumption. *PLoS Genet.* **7**, e1002033 (2011).
90. Coffee and Caffeine Genetics Consortium, C. and C. G. *et al.* Genome-wide meta-analysis identifies six novel loci associated with habitual coffee consumption. *Mol. Psychiatry* **20**, 647–656 (2015).
91. Linnér, R. K. *et al.* Genome-wide study identifies 611 loci associated with risk tolerance and risky behaviors. *bioRxiv* 261081 (2018). doi:10.1101/261081

92. Nagy, R. *et al.* Exploration of haplotype research consortium imputation for genome-wide association studies in 20,032 Generation Scotland participants. *Genome Med.* **9**, 23 (2017).
93. Sudmant, P. H. *et al.* An integrated map of structural variation in 2,504 human genomes. *Nature* **526**, 75–81 (2015).
94. Locke, A. E. *et al.* Genetic studies of body mass index yield new insights for obesity biology. *Nature* **518**, 197–206 (2015).
95. Shungin, D. *et al.* New genetic loci link adipose and insulin biology to body fat distribution. *Nature* **518**, 187–196 (2015).
96. Scott, R. A. *et al.* An expanded genome-wide association study of type 2 diabetes in Europeans. *Diabetes* **66**, 2888–2902 (2017).
97. Elks, C. E. *et al.* Thirty new loci for age at menarche identified by a meta-analysis of genome-wide association studies. *Nat. Genet.* **42**, 1077–85 (2010).
98. Couch, F. J. *et al.* Identification of four novel susceptibility loci for oestrogen receptor negative breast cancer. *Nat. Commun.* **7**, 11375 (2016).
99. Smemo, S. *et al.* Obesity-associated variants within FTO form long-range functional connections with IRX3. *Nature* **507**, 371–5 (2014).
100. Claussnitzer, M. *et al.* FTO Obesity Variant Circuitry and Adipocyte Browning in Humans. *N. Engl. J. Med.* **373**, 895–907 (2015).
101. Stefansson, H. *et al.* A common inversion under selection in Europeans. *Nat. Genet.* **37**, 129–137 (2005).
102. Chang, D. *et al.* A meta-analysis of genome-wide association studies identifies 17 new Parkinson's disease risk loci. *Nat. Genet.* **49**, 1511–1516 (2017).
103. Iqbal, K. *et al.* Tau pathology in Alzheimer disease and other tauopathies. *Biochim. Biophys. Acta - Mol. Basis Dis.* **1739**, 198–210 (2005).
104. Clavaguera, F. *et al.* Transmission and spreading of tauopathy in transgenic mouse brain. *Nat. Cell Biol.* **11**, 909–913 (2009).
105. Lee, V. M.-Y., Goedert, M. & Trojanowski, J. Q. Neurodegenerative Tauopathies. *Annu. Rev. Neurosci.* **24**, 1121–1159 (2001).
106. McGeachie, M. J. *et al.* Predicting inhaled corticosteroid response in asthma with two associated SNPs. *Pharmacogenomics J.* **13**, 306–11 (2013).
107. Tantisira, K. G. *et al.* Corticosteroid pharmacogenetics: association of sequence variants in CRHR1 with improved lung function in asthmatics treated with inhaled corticosteroids. *Hum. Mol. Genet.* **13**, 1353–1359 (2004).
108. Puglielli, L., Tanzi, R. E. & Kovacs, D. M. Alzheimer's disease: the cholesterol connection. *Nat. Neurosci.* **6**, 345–351 (2003).
109. Lambert, J. C. *et al.* Meta-analysis of 74,046 individuals identifies 11 new susceptibility loci for Alzheimer's disease. *Nat. Genet.* **45**, 1452–8 (2013).
110. Herold, C. *et al.* Family-based association analyses of imputed genotypes reveal genome-wide significant association of Alzheimer's disease with OSBPL6, PTPRG, and PDCL3. *Mol. Psychiatry* **21**, 1608–1612 (2016).
111. Beecham, G. W. *et al.* Genome-wide association meta-analysis of neuropathologic features of Alzheimer's disease and related dementias. *PLoS Genet.* **10**, e1004606 (2014).
112. Brown, M. A method for combining non-independent, one-sided tests of significance. *Biometrics* **31**, 987–992 (1975).
113. Tian, C. *et al.* Analysis and application of European genetic substructure using 300 K SNP

information. *PLoS Genet.* **4**, 0029–0039 (2008).
114. Welter, D. *et al.* The NHGRI GWAS Catalog, a curated resource of SNP-trait associations. *Nucleic Acids Res.* **42**, D1001-6 (2014).
115. Bulik-Sullivan, B. K. *et al.* An atlas of genetic correlations across human diseases and traits. *Nat. Genet.* **47**, 1236–1241 (2015).
116. Finucane, H. K. *et al.* Partitioning heritability by functional annotation using genome-wide association summary statistics. *Nat. Genet.* **47**, 1228–1235 (2015).
117. Lindblad-Toh, K. *et al.* A high-resolution map of human evolutionary constraint using 29 mammals. *Nature* **478**, 476–482 (2011).
118. Finucane, H. K. *et al.* Heritability enrichment of specifically expressed genes identifies disease-relevant tissues and cell types. *Nat. Genet.* **50**, 621–629 (2018).
119. Ward, L. D. & Kellis, M. HaploReg: a resource for exploring chromatin states, conservation, and regulatory motif alterations within sets of genetically linked variants. *Nucleic Acids Res.* **40**, D930-4 (2012).
120. Ward, L. D. & Kellis, M. HaploReg v4: systematic mining of putative causal variants, cell types, regulators and target genes for human complex traits and disease. *Nucleic Acids Res.* **44**, D877–D881 (2016).
121. Aguet, F. *et al.* Genetic effects on gene expression across human tissues. *Nature* **550**, 204–213 (2017).
122. Fehrmann, R. S. N. *et al.* Gene expression analysis identifies global gene dosage sensitivity in cancer. *Nat. Genet.* **47**, 115–25 (2015).
123. MacArthur, D. G. & Tyler-Smith, C. Loss-of-function variants in the genomes of healthy humans. *Hum. Mol. Genet.* **19**, R125-30 (2010).
124. Inoue, N., Ikawa, M., Isotani, A. & Okabe, M. The immunoglobulin superfamily protein Izumo is required for sperm to fuse with eggs. *Nature* **434**, 234–238 (2005).
125. Kindberg, E. *et al.* A nonsense mutation (428G→A) in the fucosyltransferase FUT2 gene affects the progression of HIV-1 infection. *AIDS* **20**, 685–689 (2006).
126. Carlsson, B. *et al.* The G428A Nonsense Mutation in FUT2 Provides Strong but Not Absolute Protection against Symptomatic GII.4 Norovirus Infection. *PLoS One* **4**, e5593 (2009).
127. Thorven, M. *et al.* A homozygous nonsense mutation (428G->A) in the human secretor (FUT2) gene provides resistance to symptomatic norovirus (GGII) infections. *J. Virol.* **79**, 15351–5 (2005).
128. Wacklin, P. *et al.* Secretor genotype (FUT2 gene) is strongly associated with the composition of Bifidobacteria in the human intestine. *PLoS One* **6**, e20113 (2011).
129. Edwards, S. L., Beesley, J., French, J. D. & Dunning, M. Beyond GWASs: Illuminating the dark road from association to function. *Am. J. Hum. Genet.* **93**, 779–797 (2013).
130. Pers, T. H. *et al.* Biological interpretation of genome-wide association studies using predicted gene functions. *Nat. Commun.* **6**, 5890 (2015).
131. Campbell, T. C. A plant-based diet and animal protein: questioning dietary fat and considering animal protein as the main cause of heart disease. *J. Geriatr. Cardiol.* **14**, 331–337 (2017).
132. Wade, J., Milner, J. & Krondl, M. Evidence for a physiological regulation of food selection and nutrient intake in twins. *Am. J. Clin. Nutr.* **34**, 143–7 (1981).
133. Hasselbalch, A. L., Heitmann, B. L., Kyvik, K. O. & Sørensen, T. I. A. Studies of twins indicate that genetics influence dietary intake. *J. Nutr.* **138**, 2406–12 (2008).

134. De Castro, J. M. Heritability of diurnal changes in food intake in free-living humans. *Nutrition* **17**, 713–720 (2001).
135. Evans, L. M. *et al.* Comparison of methods that use whole genome data to estimate the heritability and genetic architecture of complex traits. *Nat. Genet.* **50**, 737–745 (2018).
136. Zheng, J. *et al.* LD Hub: a centralized database and web interface to perform LD score regression that maximizes the potential of summary level GWAS data for SNP heritability and genetic correlation analysis. *Bioinformatics* **33**, 272–279 (2017).
137. Young, A. I. *et al.* Relatedness disequilibrium regression estimates heritability without environmental bias. *Nat. Genet.* **50**, 1304–1310 (2018).
138. Zuk, O., Hechter, E., Sunyaev, S. R. & Lander, E. S. The mystery of missing heritability: Genetic interactions create phantom heritability. *Proc. Natl. Acad. Sci.* **109**, 1193–1198 (2012).
139. Wainschtein, P., Jain, D. P., Yengo, L. & Zheng, Z. Recovery of trait heritability from whole genome sequence data. 1–23 (2019).
140. Hui, Q., Jin, Z., Li, X., Liu, C. & Wang, X. FGF family: From drug development to clinical application. *Int. J. Mol. Sci.* **19**, 1–15 (2018).
141. Vilhjálmsson, B. J. *et al.* Modeling linkage disequilibrium increases accuracy of polygenic risk scores. *Am. J. Hum. Genet.* **97**, 576–592 (2015).
142. Altshuler, D. M. *et al.* Integrating common and rare genetic variation in diverse human populations. *Nature* **467**, 52–58 (2010).
143. de Vlaming, R. *et al.* Meta-GWAS Accuracy and Power (MetaGAP) calculator shows that hiding heritability is partially due to imperfect genetic correlations across studies. *PLoS Genet.* **13**, e1006495 (2017).
144. Walker, R. E., Keane, C. R. & Burke, J. G. Disparities and access to healthy food in the United States: A review of food deserts literature. *Heal. Place* **16**, 876–884 (2010).
145. Demontis, D. *et al.* Discovery of the first genome-wide significant risk loci for attention deficit/hyperactivity disorder. *Nat. Genet.* **51**, 63–75 (2019).
146. Walters, R. K. *et al.* Trans-ancestral GWAS of alcohol dependence reveals common genetic 1 underpinnings with psychiatric disorders 2 3. *Stefanie Heilmann-Heimbach* **10**, 26
147. Grove, J. *et al.* 17 Autism Spectrum Disorders Working Group of The Psychiatric Genomics Consortium, BUPGEN, Major Depressive Disorder Working Group of the Psychiatric Genomics Consortium. *bioRxiv* **33**, 42 (2017).
148. Stahl, E. *et al.* Genomewide association study identifies 30 loci associated with bipolar disorder. *bioRxiv* 173062 (2018). doi:10.1101/173062
149. Wray, N. R. *et al.* Genome-wide association analyses identify 44 risk variants and refine the genetic architecture of major depression. *Nat. Genet.* **50**, 668–681 (2018).
150. Smoller, J. W. *et al.* Identification of risk loci with shared effects on five major psychiatric disorders: A genome-wide analysis. *Lancet* **381**, 1371–1379 (2013).
151. Hill, W. D. *et al.* Molecular genetic contributions to social deprivation and household income in UK Biobank. *Curr. Biol.* **26**, 3083–3089 (2016).
152. Wood, A. R. *et al.* Defining the role of common variation in the genomic and biological architecture of adult human height. *Nat. Genet.* **46**, 1173–86 (2014).
153. Finucane, H. K. *et al.* Partitioning heritability by functional category using GWAS summary statistics. *Nat. Genet.* **47**, 1228–1235 (2015).
154. Logan, A. C. & Jacka, F. N. Nutritional psychiatry research: An emerging discipline and

its intersection with global urbanization, environmental challenges and the evolutionary mismatch. *J. Physiol. Anthropol.* **33**, 1–16 (2014).

155. Sarris, J. *et al.* Nutritional medicine as mainstream in psychiatry. *The Lancet Psychiatry* **2**, 271–274 (2015).
156. Marx, W., Moseley, G., Berk, M. & Jacka, F. Nutritional psychiatry: the present state of the evidence. *Proc. Nutr. Soc.* **76**, 427–436 (2017).
157. Cordain, L. *et al.* Origins and evolution of the Western diet: health implications for the 21st century. *Am. J. Clin. Nutr.* **81**, 341–354 (2005).
158. Sarris, J. *et al.* Nutraceuticals for major depressive disorder- more is not merrier: An 8-week double-blind, randomised, controlled trial. *J. Affect. Disord.* **245**, 1007–1015 (2019).
159. Grosso, G. *et al.* Dietary n-3 PUFA, fish consumption and depression: A systematic review and meta-analysis of observational studies. *J. Affect. Disord.* **205**, 269–281 (2016).
160. Jacka, F. N. *et al.* A randomised controlled trial of dietary improvement for adults with major depression (the 'SMILES' trial). *BMC Med.* **15**, 1–13 (2017).
161. Ruxton, C. H. S., Gardner, E. J. & McNulty, H. M. Is Sugar Consumption Detrimental to Health? A Review of the Evidence 1995—2006. *Crit. Rev. Food Sci. Nutr.* **50**, 1–19 (2009).
162. Pelsser, L. M. *et al.* Effects of a restricted elimination diet on the behaviour of children with attention-deficit hyperactivity disorder (INCA study): A randomised controlled trial. *Lancet* **377**, 494–503 (2011).
163. El Ghoch, M., Calugi, S. & Dalle Grave, R. The effects of low-carbohydrate diets on psychosocial outcomes in obesity/overweight: A systematic review of randomized, controlled studies. *Nutrients* **8**, 1–13 (2016).
164. Tremblay, F., Lavigne, C., Jacques, H. & Marette, A. Role of Dietary Proteins and Amino Acids in the Pathogenesis of Insulin Resistance. *Annu. Rev. Nutr.* **27**, 293–310 (2007).
165. Yoon, M.-S. The Emerging Role of Branched-Chain Amino Acids in Insulin Resistance and Metabolism. *Nutrients* **8**, 405 (2016).
166. Fretts, A. M. *et al.* Consumption of meat is associated with higher fasting glucose and insulin concentrations regardless of glucose and insulin genetic risk scores: a meta-analysis of 50,345 Caucasians. *Am. J. Clin. Nutr.* **102**, 1266–78 (2015).
167. Song, M. *et al.* Association of animal and plant protein intake with all-cause and cause-specific mortality. *JAMA Intern. Med.* **176**, 1453–1463 (2016).
168. Westerterp-Plantenga, M. S. & Lejeune, M. P. G. M. Protein intake and body-weight regulation. *Appetite* **45**, 187–90 (2005).
169. Weigle, D. S. *et al.* A high-protein diet induces sustained reductions in appetite, ad libitum caloric intake, and body weight despite compensatory changes in diurnal plasma leptin and ghrelin concentrations. *Am. J. Clin. Nutr.* **82**, 41–8 (2005).
170. Ankarfeldt, M. Z. *et al.* Body composition, dietary protein and body weight regulation. Reconciling conflicting results from intervention and observational studies? *PLoS One* **9**, e101134 (2014).
171. Cummins, S. & Macintyre, S. 'Food deserts' - evidence and assumption in health policy making. *Br. Med. J.* **325**, 436–438 (2002).
172. Amani, R. Is dietary pattern of schizophrenia patients different from healthy subjects? *BMC Psychiatry* **5**, 3–7 (2007).
173. Adab, P., Pallan, M. & Whincup, P. H. Is BMI the best measure of obesity? *Br. Med. J.* **360**, k1274 (2018).

174. Anderson, J. J. *et al.* Adiposity among 132 479 UK Biobank participants; contribution of sugar intake vs other macronutrients. *Int. J. Epidemiol.* dyw173 (2016). doi:10.1093/ije/dyw173
175. Gruberg, L. *et al.* The impact of obesity on the short-term and long-term outcomes after percutaneous coronary intervention: The obesity paradox? *J. Am. Coll. Cardiol.* **39**, 578–584 (2002).
176. Hainer, V. & Aldhoon-Hainerová, I. Obesity paradox does exist. *Diabetes Care* **36 Suppl 2**, S276-81 (2013).
177. Levine, M. E. *et al.* Low protein intake is associated with a major reduction in IGF-1, cancer, and overall mortality in the 65 and younger but not older population. *Cell Metab.* **19**, 407–417 (2014).
