# Supplementary material for: Genomic analysis of diet composition finds novel loci and associations with health and lifestyle
Source: Mol Psychiatry. 2020 May 11;26(6):2056–69. doi: 10.1038/s41380-020-0697-5 (PMC7767645; doi:10.1038/s41380-020-0697-5)
Supplement: Supplementary file 2 — Supplementary figures [file 41380_2020_697_MOESM2_ESM.pdf]

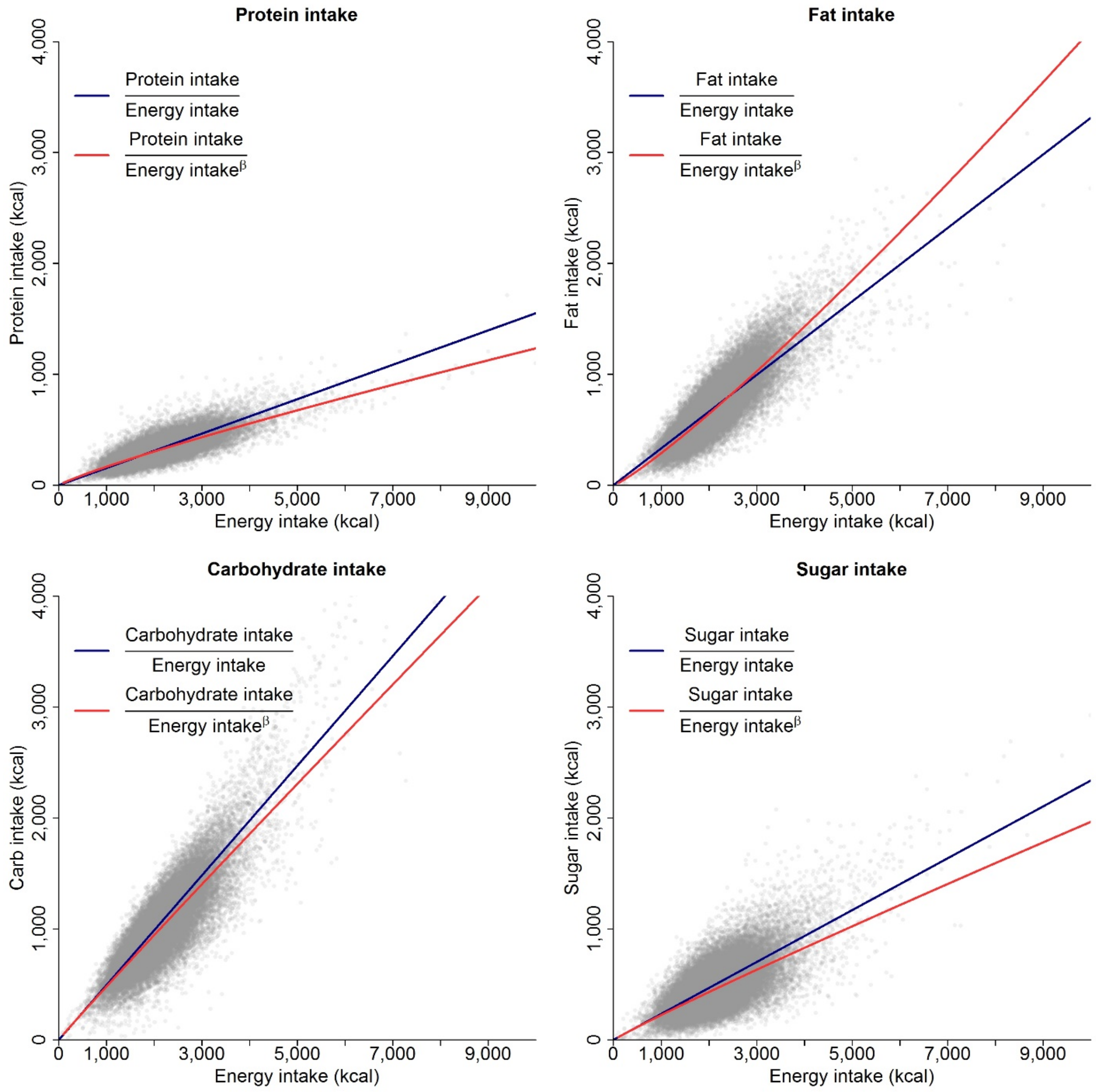

Extended Data Figure 1 | Non-linear phenotypic scaling between macronutrient intake and total energy intake. Scatterplots of macronutrient intake (y-axis) versus total energy intake (x-axis) in European-ancestry individuals from the first release of genetic data from the UK Biobank (N = 43,788). The blue and red lines denote the different lines of fit. The blue line represents the average "macronutrient density" (i.e., average energy intake from the focal macronutrient as observed in the full sample, across the different values of x_i). The red line represents the regression line of the multiplicative regression model of macronutrient intake on energy intake, where $y_i = \hat{\alpha} * x^{\hat{\beta}}$.

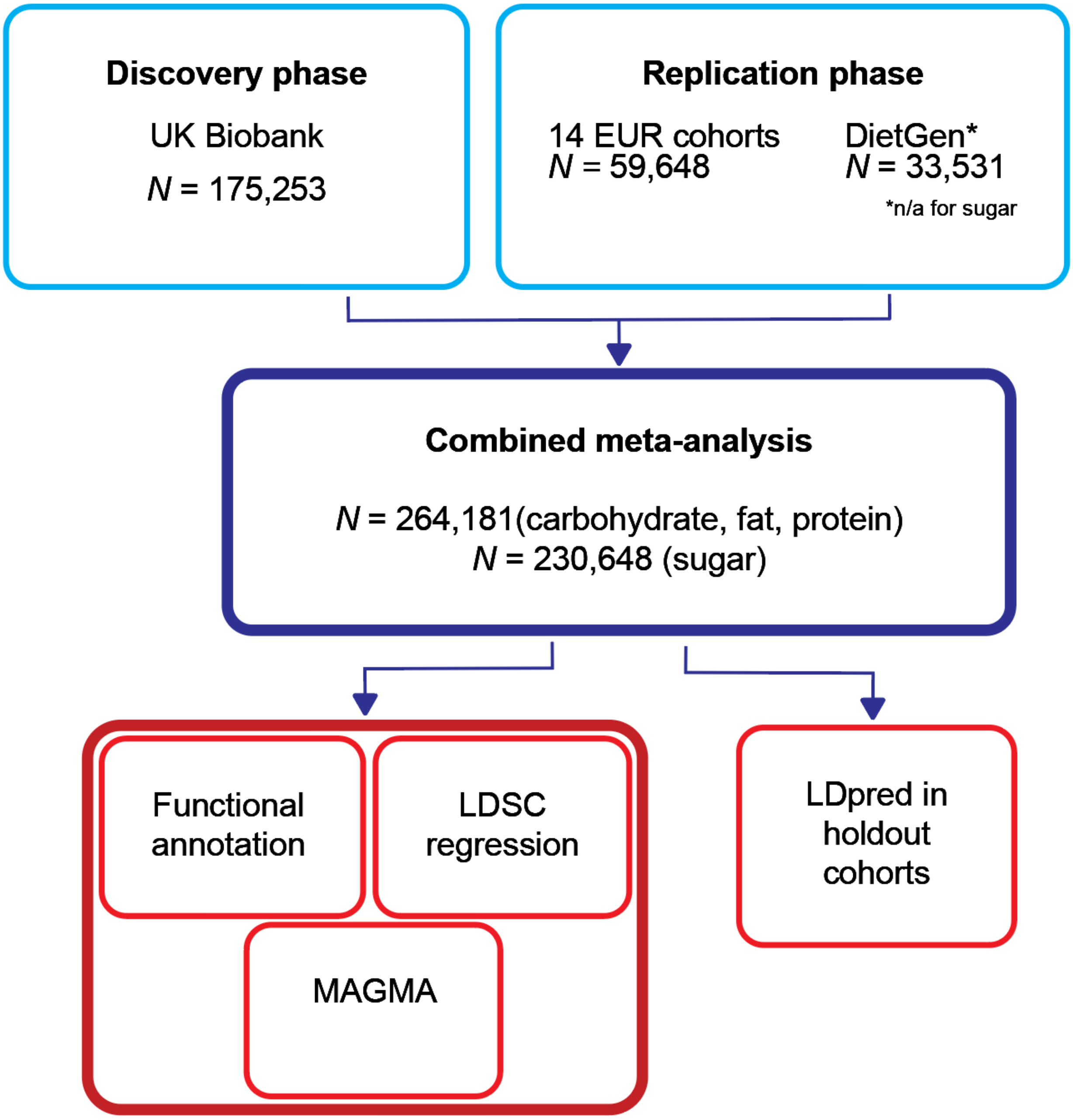

Extended Data Figure 2 | Flow chart. Schematic representation of the study design.

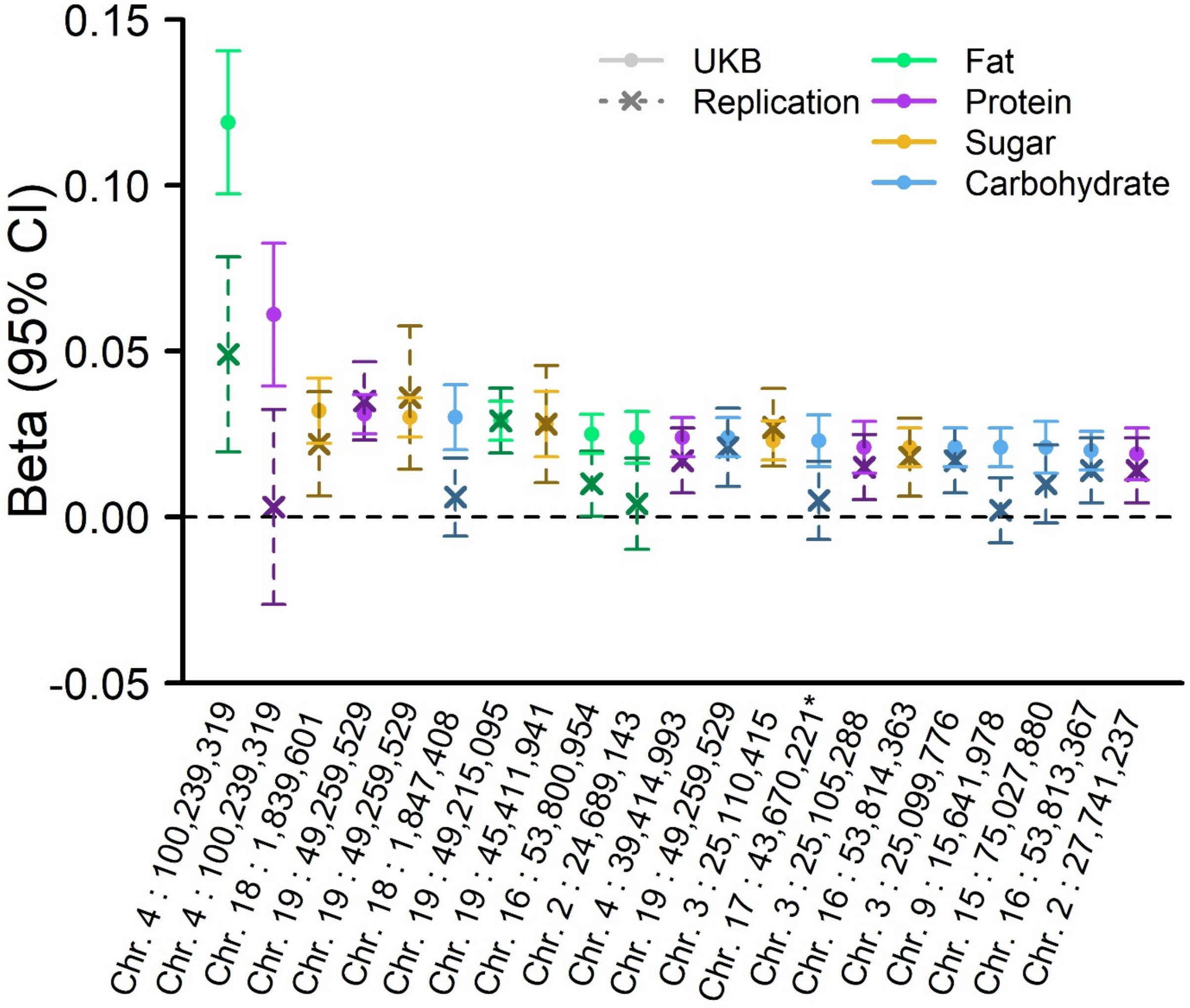

Extended Data Figure 3 | Replication of 21 lead SNPs from the discovery GWAS of diet composition. Estimated effect sizes (measured in standard deviations of the diet composition phenotype per effect allele) and 95% confidence intervals for 20 diet composition lead SNPs and 1 proxy-lead SNP (noted with an asterisk, *), in the discovery versus the combined replication phase. SNPs are listed from left to right in descending order of their beta in the discovery GWAS, and coded in such a way that the effect allele is always trait-increasing in the discovery phase. Of the 21 lead or proxy-lead SNPs, all have the anticipated sign in the replication phase, and 16 replicate at the 0.05, one-sided significance level. See **Supplementary Information section 4** for additional details.

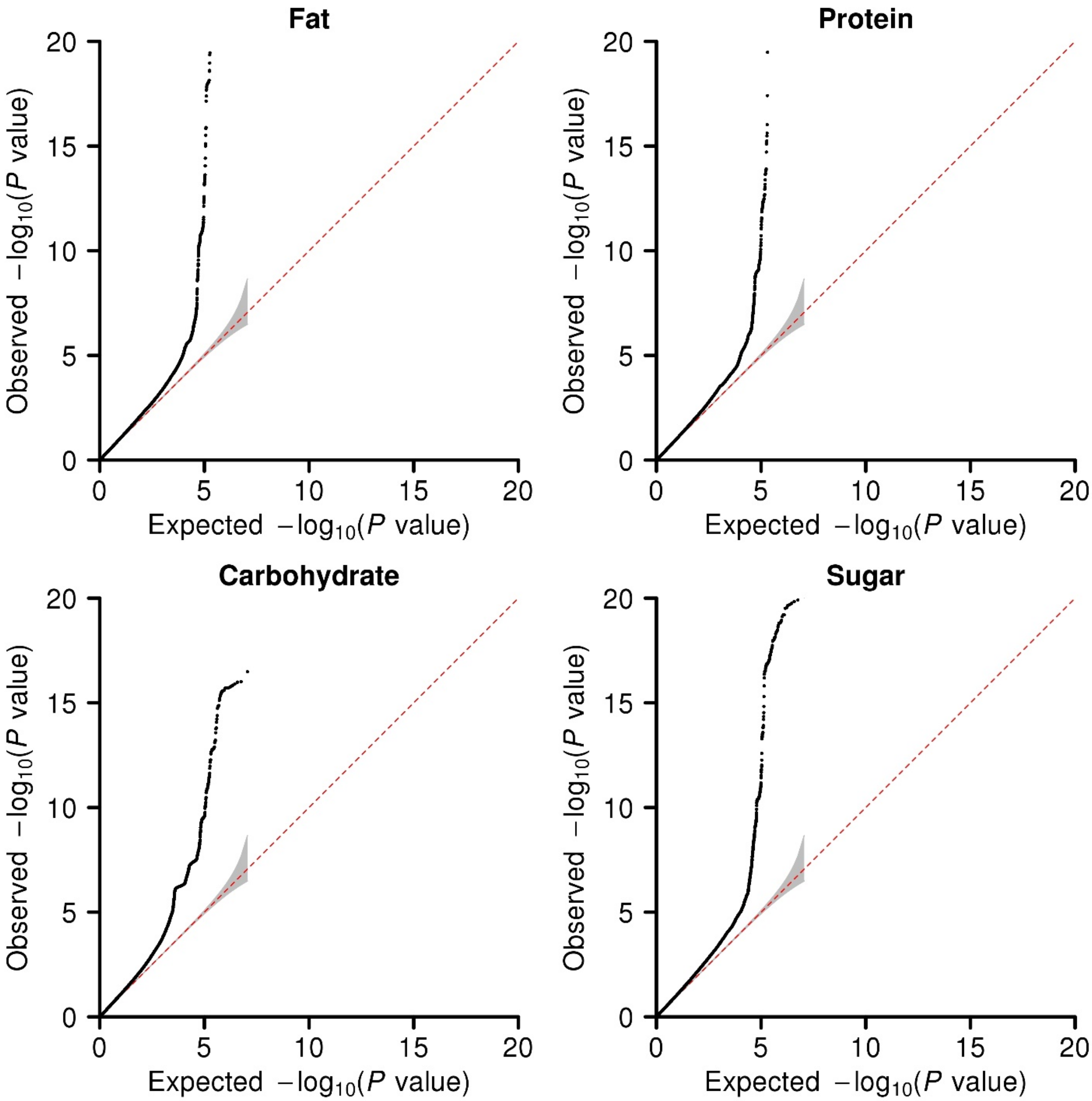

Extended Data Figure 4 | QQ-plot Quantile-Quantile (QQ) plot for the *P* values from the GWAS (for the full meta-analysis samples, inflated by the LDSC intercept) for the four different diet composition phenotypes, which compares the expected *P* values (based on the *P* value beta distribution) to the observed *P* values from the GWAS. Deviation from the expected values (represented with the red, slanted diagonal) represents polygenicity of the trait, and potentially bias due to (for instance) remnant population stratification. The shaded region represents the 95% confidence interval region.

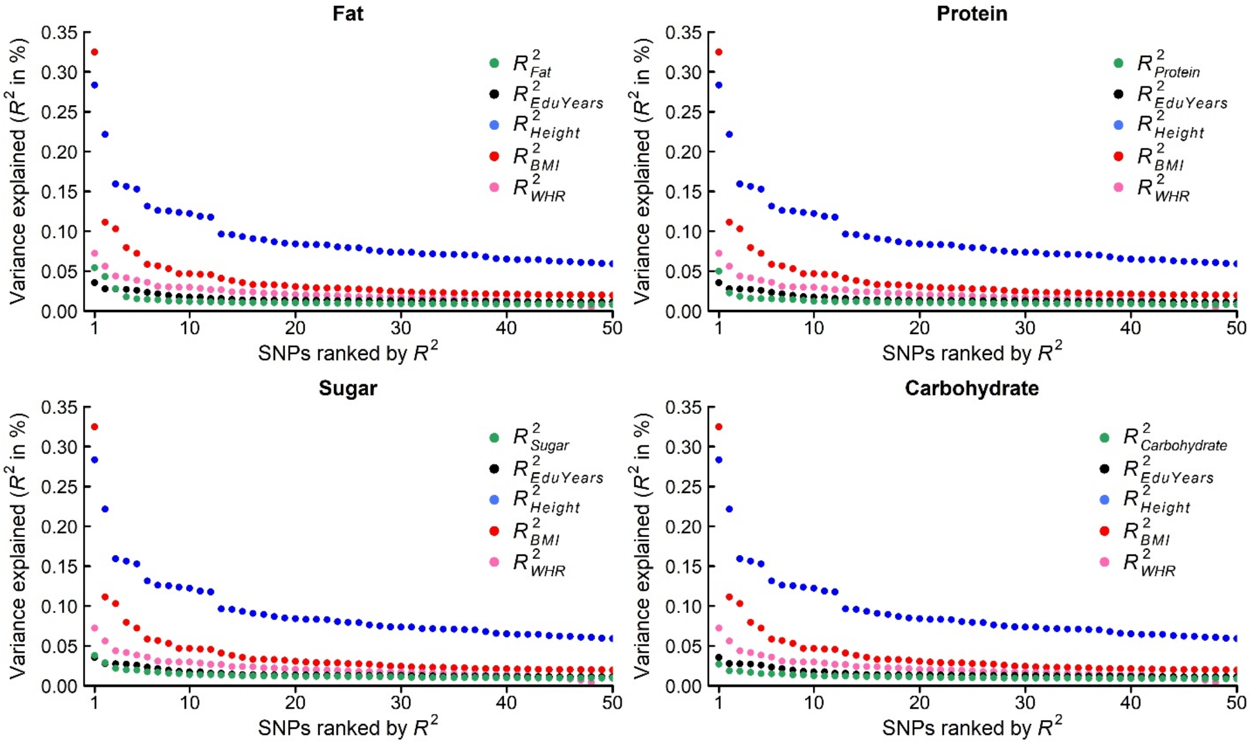

Extended Data Figure 5 | Distribution of effect sizes of the top 50 diet composition lead SNPs, compared with various phenotypes. Variance explained (R^2), with the SNPs ranked by their explained phenotypic variance (R^2). The diet composition effect sizes are compared against the top 50 previously reported for height and for body mass index (BMI), educational attainment (EduYears), and waist-to-hip ratio adjusted for BMI (WHR). The effect sizes for height, BMI, and WHR are based on the GIANT consortium's publicly available results for pooled analyses restricted to European-ancestry individuals; the effect sizes for EduYears are from Okbay *et al.*

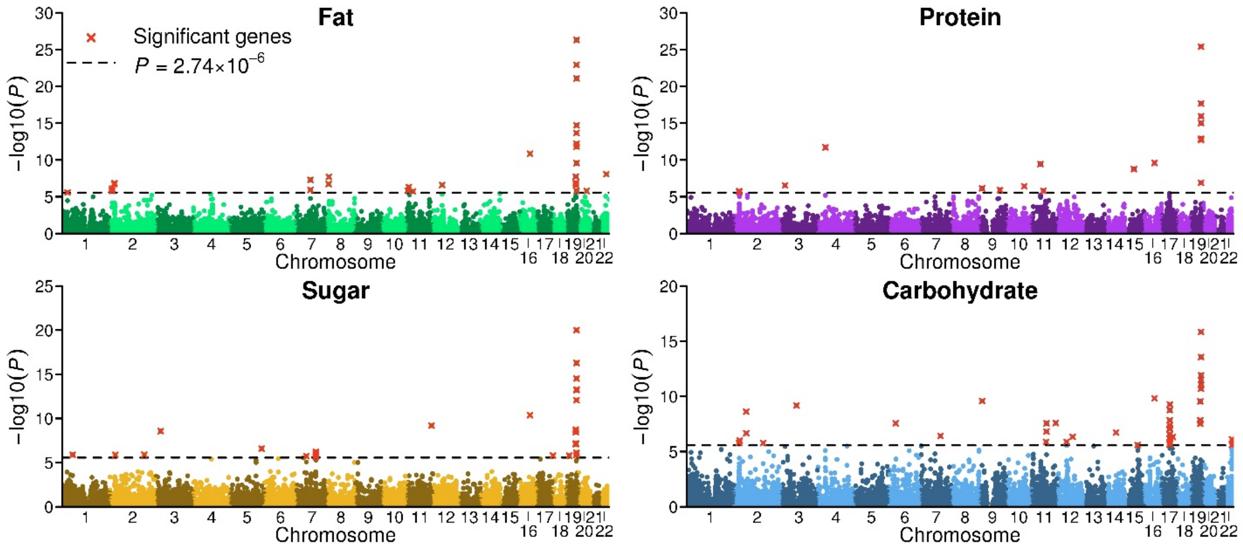

Extended Data Figure 6 | Manhattan plot of the MAGMA gene-based analysis of diet composition. The *x*-axis displays chromosomal position of the gene midpoint, and the *y*-axis displays $-\log_{10} P$ value of each gene. The dashed line marks the Bonferroni-corrected significance threshold, where the correction is for 18,224 genes. Significant genes are marked with a red cross.

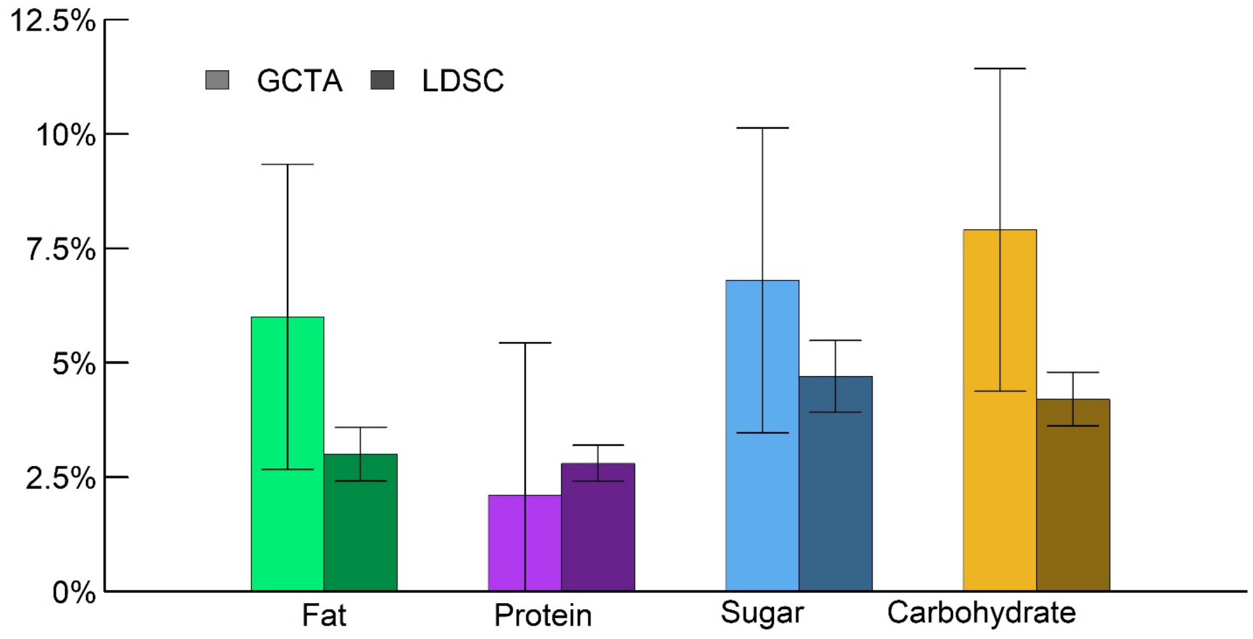

Extended Data Figure 7 | SNP-based heritability estimates of diet composition. SNP heritability was estimated with GCTA-GREML and LD Score regression. GREML was estimated using a random draw of 30,000 individuals from the UKB GWAS sample, from which we excluded cryptically related individuals, and using all genotyped SNPs with MAF > 0.01. LD Score heritability was estimated using HapMap3 SNPs with MAF > 0.01. See **Supplementary Information 7** and **Supplementary Table 7.1** for additional details.

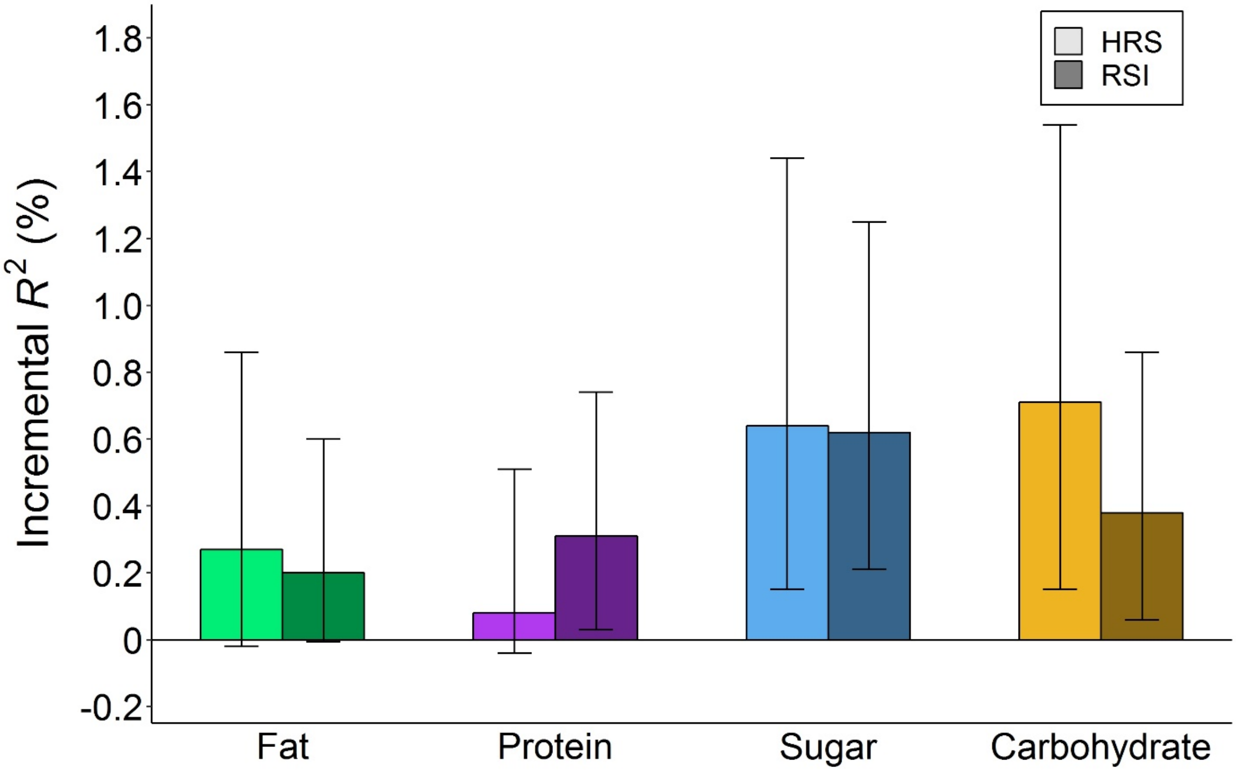

Extended Data Figure 8 | Out-of-sample prediction estimates of diet composition with polygenic scoring. Incremental R^2 is defined as the increase in R^2 from adding the polygenic score for the focal macronutrient to a regression of the macronutrient on control variables for sex, age, sex × age, and the top ten principal components of the genotype matrix. The polygenic scores were constructed using LDpred with Gaussian mixture weight of 1. The validation cohorts are the HRS and RSI cohorts. **Supplementary Information section 8** and **Supplementary Table 8.1** for additional details.

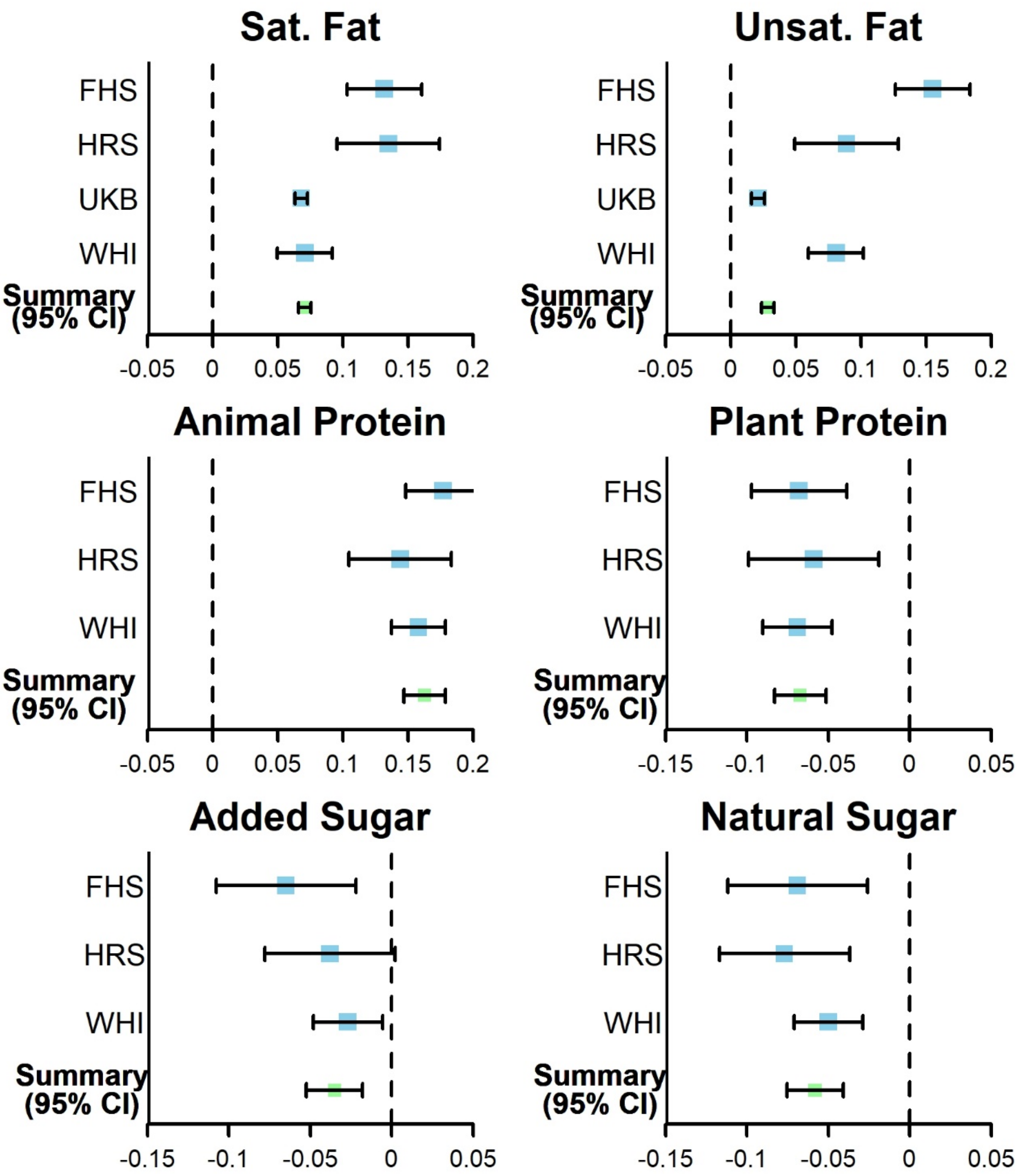

Extended Data Figure 9 | Phenotypic associations between macronutrient subtypes and Body Mass Index (BMI). Phenotypic associations are displayed in terms of standardized regression coefficients (with 95% confidence intervals). These coefficients were obtained from a regression of BMI on the focal macronutrient and several covariates (sex, age, educational attainment, and household income). FHS = Framingham Heart Study (*N* = 4,428), HRS = Health and Retirement Study (*N* = 2,385), UKB = UK Biobank (*N* = 157,852), WHI = Women's Health Initiative (*N* = 8,500). The summary estimate was based on fixed-effects, inverse-variance-weighted meta-analysis.
